# Markovian state models uncover casein kinase 1 dynamics that govern circadian period

**DOI:** 10.1016/j.bpj.2025.09.022

**Published:** 2025-09-18

**Authors:** Clarisse Gravina Ricci, Jonathan M. Philpott, Megan R. Torgrimson, Alfred M. Freeberg, Rajesh Narasimamurthy, Emilia Pécora de Barros, Rommie Amaro, David M. Virshup, J. Andrew McCammon, Carrie L. Partch

**Affiliations:** 1 Department of Chemistry and Biochemistry, University of California, San Diego, San Diego, California; 2 Department of Chemistry and Biochemistry, University of California, Santa Cruz, Santa Cruz, California; 3 Program in Cancer and Stem Cell Biology, Duke-NUS Medical School, Singapore, Singapore; 4 Department of Pediatrics, Duke University Medical Center, Durham, North Carolina; 5 Center for Circadian Biology, University of California, San Diego, San Diego, California; 6 Howard Hughes Medical Institute, University of California, Santa Cruz, Santa Cruz, California

## Abstract

Circadian rhythms in mammals are tightly regulated through phosphorylation of period (PER) proteins by casein kinase 1 (CK1, subtypes δ and ε). CK1 acts on at least two different regions of PER with opposing effects: phosphorylation of phosphodegron regions leads to PER degradation, whereas phosphorylation of the familial advanced sleep phase (FASP) region leads to PER stabilization. To investigate how substrate selectivity is encoded by the conformational dynamics of CK1, we performed a large set of independent molecular dynamics simulations of wild-type CK1 and the *tau* mutant (R178C) that biases kinase activity toward a phosphodegron. We used Markovian state models to integrate the simulations into a single model of the conformational landscape of CK1 and used Gaussian accelerated molecular dynamics to build the first molecular model of CK1 and the unphosphorylated FASP motif. These findings were biochemically validated using in vitro kinase assays and provide a mechanistic view of CK1, establishing how the activation loop acts as a key molecular switch to control substrate selectivity. We show that the wild-type CK1 prefers a “loop down” conformation that binds FASP, whereas the *tau* mutant favors an alternative conformation of the activation loop and significantly accelerates the dynamics of CK1. This reshapes the binding cleft in a way that impairs FASP binding and would ultimately lead to PER destabilization. Finally, we identified a potential binding pocket that could be targeted to influence the conformational state of this molecular switch and lead to predictable changes in circadian period. Our integrated approach offers a detailed model of CK1’s conformational landscape and its relevance to normal, mutant, and druggable circadian timekeeping.

## Significance

Disruption of circadian rhythms alters the temporal orchestration of vital cellular processes and increases the propensity for sleep disorders, metabolic disease, and cancer. Circadian rhythms are generated by a gene expression program controlled at the cellular level by a molecular clock composed of dedicated clock proteins. Among the essential protein characters is casein kinase 1 (CK1), which acts on multiple clock protein substrates. A delicate balance of CK1 activity on these substrates is crucial for proper circadian timekeeping, highlighting CK1 as a promising drug target to tune clock timing. This work describes the conformational landscape of CK1 that underlies its substrate specificity and provides molecular insight for pharmacologic development that could modulate CK1 function for those suffering from clock-related syndromes.

## Introduction

One of the greatest achievements of life on Earth is the ability of organisms to anticipate the terrestrial cycles of light and darkness. From prokaryotes to mammals, life forms take advantage of day and night to perform cellular tasks in a coherent way by following an internal clock (1,2,3,4,5). Although the biological clock synchronizes to the solar day by making use of external cues, its intrinsic “ticking” pace is dictated by an internal core oscillator present in nearly every cell and displaying a period of ∼24 h (6). In mammals, the core oscillator consists of an interlocked transcription/translation feedback loop that generates daily oscillations in gene expression (3,7). This results in a circadian (about a day) expression of proteins involved in behavior, development, metabolism, DNA repair, and more (6). Mutations causing the intrinsic period to be significantly different from ∼24 h can prevent organisms from successfully synchronizing their clocks to the solar day. This results in social jetlag and sleep disorders, which in the long run can interfere with our immune response (8,9) and trigger pathologies such as metabolic syndrome, diabetes, and cancer (6,10,11,12,13,14,15,16,17,18). In this scenario, understanding the molecular underpinnings of the clock could unlock new pharmacological targets to treat a wide range of diseases.

**Figure 1:**
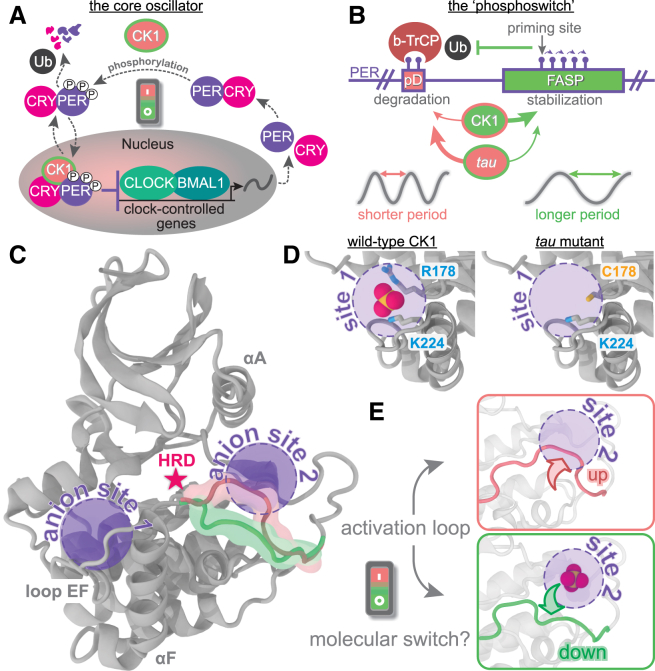
The CK1 activation loop acts as a molecular switch to control PER phosphorylation and circadian period. (*A*) Simple schematic view of the transcriptional-translational feedback loop at the core of the human clock. (*B*) The circadian period is finely regulated by a phosphoswitch mechanism controlling PER stability. (*C*) Apo structure of CK1δ (PDB: 1CKJ), highlighting the location of the catalytic HRD motif (*red star*), the two conserved anion binding sites (*purple circles*), and the alternative conformations of the activation loop (*pink and green*). (*D*) A sulfate anion is bound to the first anion site via two positively charged clamps (R178 and K224). The R178C mutation in *tau* impairs the ability of this site to bind anions. (*E*) Alternative conformations of the activation loop in *tau* (PDB: 6PXN), showing that the loop up conformation (*pink*) sterically blocks the second anion site.

In humans, the transcription/translation feedback loop is formed by a transcription activator complex, CLOCK:BMAL1, and a repressor complex formed by period proteins (PER1 and PER2), cryptochromes (CRY1 and CRY2), and the protein casein kinase 1 (subtypes δ and ε, hereafter jointly referred to as CK1) (Fig. 1 *A*) (19,20,21,22,23). The CLOCK:BMAL1 dimer activates the transcription of many circadian-controlled genes, including those of their repressors (PERs and CRYs), leading to daily oscillations in repressor expression. Because PER is the stoichiometric limiting factor in the assembly of the CK1:PER:CRY repressor complex (24), its abundance and stability are correlated with the duration of the intrinsic circadian period. The molecular mechanisms regulating the life span of PER proteins thus provide a direct link to circadian period. At the very center of this switch is CK1, which modulates PER stability through posttranslational modifications (25,26,27,28,29,30) and is thought to confer temperature insensitivity to circadian rhythms (31,32).

CK1 is a ubiquitously expressed serine/threonine kinase with activity against a broad variety of substrates in all cell types (33,34). Orthologs of CK1 have been implicated in timekeeping in a variety of eukaryotic organisms ranging from green algae to humans (35,36,37,38), denoting a well-conserved and far-reaching role across species. CK1 displays the typical two-lobed kinase architecture; however, it is not regulated by phosphorylation of its activation loop (Fig. 1 *C*)(33,39,40). Instead, the kinase domain of CK1 is constitutively active (34,41,42), and two highly conserved anion binding pockets play important regulatory roles, including substrate selectivity and recognition (40,43,44). CK1 preferentially acts on negatively charged or primed substrates, such as those with a D/E/pSxxS consensus motif (40,45). Recently, structures of CK1 show that it uses these anion binding sites to bind phosphorylated peptides, either in its own disordered tail (46) or its circadian substrate, PER2 (47). CK1 phosphorylates at least two different regions on PER with antagonist effects on its stability in a phosphoswitch mechanism (Fig. 1 *B*) (48,49,50). Interestingly, the CK1-dependent phosphorylation sites that form this phosphoswitch in PER do not display this consensus motif and are slow, rate-limiting steps for the circadian clock (25,48). Phosphorylation at phosphodegron (pD) regions recruits the E3 ubiquitin ligase, β-TrCP, and leads to PER degradation, with the ultimate effect of shortening the circadian period (51,52,53,54,55). On the other hand, phosphorylation of five consecutive serines in the FASP region (25) found within the CK1-binding domain of PER leads to its stabilization through feedback inhibition that attenuates kinase activity at the pD (47). Mutation of the first serine in this region causes abnormally short circadian periods and FASP (56,57). Other mutations in CK1/CK1 orthologs (36,38,43,58,59,60,61,62,63) or in its phosphorylation sites on PER/PER orthologs (49,59) can induce remarkable changes to the intrinsic circadian period, underscoring the central importance of the phosphoswitch for clock timing. Thus far, little is known about the conformational mechanism by which CK1 balances its activity between the pD and FASP regions.

Because its activity and selectivity influences clock periodicity, CK1 is gaining traction as an effective pharmacological target for dysfunctional clocks (27,64). ATP-competitive CK1 inhibitors capable of lengthening the circadian rhythm have been identified (27,65,66,67,68,69,70,71,72), but, unlike many mutations in the enzyme, no small molecules are capable of shortening the period (36,38,58,62). To develop highly specific circadian drugs that diversify our ability to modulate period length, more information is needed on the molecular factors dictating CK1 regulation and substrate selectivity.

The CK1-based circadian mutant *tau* (73) shortens the circadian period by approximately 4 h (61,63). In this mutant, an arginine in the first anion binding pocket is replaced by a cysteine (R178C) (Fig. 1 *D*), impairing the pocket’s ability to bind negatively charged groups in primed or acidic substrates (44,73). This site lies near loop EF (L-EF), a flexible region generally implicated in substrate binding by kinases (40,45) and, in the case of CK1, is specifically required for temperature-compensated activity (31). We showed that *tau* simultaneously reduces the activity on the FASP region and increases the activity on the pD site, inverting substrate selectivity on PER relative to the wild-type (WT) enzyme (Fig. 1 *B*) (73). This makes the *tau* mutant an ideal system to study the conformational mechanisms of how CK1 toggles the phosphoswitch. Supported by molecular dynamics (MD) simulations, the crystal structure of *tau* revealed two alternative conformations of the activation loop (“up” and “down,” Fig. 1 *E*), hinting at a two-state conformational switch at the core of CK1 substrate selectivity. For circadian substrates, these are the two predominant states of CK1, and they contain the structural features that generally define an active kinase: the DFG motif is positioned inward toward the ATP-binding site with specific dihedral angles between residues that allow the kinase to be catalytically primed, a salt bridge is formed between the helix αC and the N-terminal domain, and the activation loop is positioned correctly at both the C-terminal and N-terminal regions to bind ATP and substrates (74). Interestingly, the less frequent “loop up” conformation of the WT CK1 sterically abolishes the second anion binding pocket, which could be a determining factor for substrate binding and/or product inhibition (73,75).

To investigate how substrate selectivity is encoded in the conformational dynamics of CK1, here we performed a large set of unbiased MD simulations of WT CK1 and the *tau* mutant. These simulations were integrated into a Markov state model (MSM) (76,77,78,79) to describe the free energy landscape bridging “loop up” and “loop down” conformations in CK1. MSMs have been emerging as a powerful framework to uncover slow dynamics in complex biomolecular systems (80,81,82) and to quantify differences between protein ensembles (83,84,85), many times with significant implications for drug discovery and design (86,87,88,89,90). Here, we additionally used Gaussian accelerated MD (GaMD) (91,92,93,94) to boost the sampling of the CK1 conformational landscape and to build the first molecular model of the interaction of CK1 with the priming motif of FASP. Using in vitro kinase assays, we biochemically validated this binding model and identified essential residues necessary for proper CK1 phosphorylation of the stabilizing FASP region. Additionally, we found that FASP binds the “loop down” conformation, whereas the *tau* mutant accelerates CK1 dynamics, favors the “loop up” conformation of the activation loop, and reshapes the binding cleft in a way that hinders FASP binding and ultimately destabilizes PER. Our combined approach provides a comprehensive model of the conformational landscape of CK1 and its implications for circadian timekeeping by establishing the activation loop as a key molecular switch for substrate selectivity. Finally, FTMap identified a potential pocket that could be targeted to bias the “loop up” conformation and ultimately shorten the circadian period.

## Materials and methods

### Molecular dynamics simulations and related analyses

#### Initial structures

To simulate *tau* CK1δ, we used PDB: 6PXN (73), starting simulations from both the “loop up” conformation (chain A) and from the “loop down” conformation (chain B). To launch equivalent simulations with the WT CK1, we used PDB: 1CKJ (40), which has the activation loop crystallized both in “up” (chain A) and “down” (chain B) conformations. The structures from PDB: 1CKJ contained two (chain A) or three (chain B) WO_4_^−2^ anions, which were computationally replaced by SO_4_^2−^ anions at the same positions, to make the simulations of WT CK1δ comparable to the *tau* simulations.

#### Systems setup

Before simulations, the proteins were minimized using Maestro (Schrödinger), and the final protonation states were estimated using the H++ server (95). All systems were solvated in a preequilibrated cubic TIP3P (96) water box with at least 15 Å between the protein and the box boundaries. The systems net charge was neutralized with Na+ or Cl− counterions. Parameters for proteins and counterions were extracted from the ff14SB force field (97), whereas parameters for the SO_4_^2−^ anions were extracted from the Generalized Amber Force Field (GAFF) (98) and adjusted as proposed (99).

#### Systems equilibration

Minimization and equilibration were performed with AMBER 16 (100), using the following protocol: 1) 2000 steps of energy minimization with a 500 kcal mol^−1^ Å^−1^ position restraint on protein and SO_4_^2−^ anions; 2) 1000 steps of energy minimization with a 500 kcal mol^−1^Å^−1^ position restraint on protein atoms only; 3) 2000 steps of energy minimization without position restraints; 4) 50 ps of NVT simulation, with gradual heating to a final temperature of 300 K, with 10 kcal mol^−1^Å^−1^ position restraint on protein and SO_4_^2−^ anions; 5) 1 ns of NPT simulation to equilibrate the density (or final volume of the simulation box). Temperature was kept at 300 K using the Langevin thermostat and a collision frequency of 2 ps^−1^. After equilibrating the volume, we ran additional 100 ns of simulations in the NVT regime for each system, using a time step of 2 fs, and all bonds involving hydrogen atoms were restrained with SHAKE (101). The PME method (102) was used to calculate electrostatic interaction using periodic boundary conditions, and a 12-Å cutoff was used to truncate nonbonded short-range interactions.

#### Gaussian accelerated MD simulations

After equilibration, we ran GaMD simulations with AMBER 17 (103). To boost the exploration of the conformational space, additional acceleration parameters were used, as described previously (91). All systems had a threshold energy E = Vmax and were subjected to a dual boost acceleration of both the dihedral and the total potential energies. To optimize the acceleration parameters, we first ran 2 ns of MD simulations with no boost potential, during which the minimum, maximum, average, and standard deviation (Vmin, Vmax, Vav, σavg) of the total potential and dihedral energies were estimated and used to derive boost potentials as detailed previously (91). These potentials were used to start 50 ns of GaMD simulations, during which the boost statistics and boost potentials were updated until the maximum acceleration was achieved. The maximum acceleration was constrained setting the upper limit of the standard deviation of the total boost potential to be 6 kcal/mol. We ran five replicas of production GaMD simulations for each system with fixed acceleration parameters derived from the previous equilibration stage. Each replica started from the same initial conformation, but the atoms were given different initial velocities, consistent with a Maxwell-Boltzmann distribution at 300 K. Each production simulation ran for 500 ns, totalizing 2.5 μs of sampling for each system, and 15 μs in total.

#### Conventional MD simulations

To start the MD simulations that would be used to build the MSMs, we extracted conformations from GaMD. For each system (WT and *tau*), 10 different conformations were selected, as described in the supplemental materials and methods, Section A. For each initial structure, we launched three independent MD replicas (with different initial velocities) for 300 ns, totalizing 9 μs for each system (WT or *tau*). After these finished running, we randomly selected 20 more structures from the new trajectories and relaunched two independent MD replicas for 300 ns, adding 12 μs to the total time simulated for each system. Conventional MD simulations were performed with the same methods and parameters as described in the “systems equilibration” subsection.

#### Markov state models

We used PyEmma version 2.5.7 (104) to process the trajectories and to build, validate, and analyze the MSMs. More details on the construction of the MSM models, including input features, parameters, and validation tests, are described in the supplemental materials and methods, Section B. Jupyter notebooks for the MSM analyses of WT and *tau* CK1 are available at https://github.com/cpartch/CK1, and full trajectories (23 GB data) are available upon request from jmmccammon@ucsd.edu.

#### Model of CK1 bound to FASP peptide

Due to space limitations, methodological details on the simulations used to create this model can be found in the supplemental materials and methods, Section C.

#### Mapping of binding pockets on the CK1 surface

This analysis was performed by subjecting representative MD-derived conformations of WT and *tau* CK1 to FTMap (105), a fast computational approach that uses small organic probes to identify consensus sites (or pockets) that are likely to bind drug-like molecules. Due to space limitations, more details on how this analysis was performed are described in the supplemental materials and methods, Section E.

### Expression and purification of recombinant proteins

All plasmid purification was carried out in *Escherichia coli* DH5a cells. Proteins were expressed from a pET22-based vector in *Escherichia coli* BL21 (DE3) Rosetta2 cells (Sigma Aldrich) based on the Parallel vector series (106). All FASP peptides were expressed downstream of an N-terminal TEV-cleavable His-NusA tag. Human CK1δ catalytic domains (CK1, residues 1–317) were all expressed in BL21 (DE3) Rosetta2 cells (Sigma Aldrich) with a TEV-cleavable His-GST tag. Mutations were made using standard site-directed mutagenesis protocols and validated by sequencing. All proteins and peptides expressed from parallel vectors have an N-terminal vector artifact (GAMDPEF) remaining after TEV cleavage, and the peptides have a tryptophan and polybasic motif (WRKKK) following the vector artifact. Cells were grown in LB media (for natural abundance growths) or M9 minimal media with the appropriate stable isotopes (i.e., ^15^N/^13^C for NMR) as done before (25) at 37°C until the O.D._600_ reached ∼0.8; expression was induced with 0.5 mM IPTG, and cultures were grown for approximately 16–20 h more at 18°C.

For CK1 protein preps, cells were lysed in 50 mM Tris (pH 7.5), 300 mM NaCl, 1 mM TCEP, and 5% glycerol using a high-pressure extruder (Avestin) or sonicator (Fisher Scientific) on ice. His-GST-CK1 fusion proteins were purified using Glutathione Sepharose 4B resin (GE Healthcare) using standard approaches and eluted from the resin using phosphate buffered saline with 25 mM reduced glutathione. His-TEV protease was added to cleave the His-GST tag from CK1 at 4°C overnight. Cleaved CK1 was further purified away from His-GST and His-TEV using Ni-NTA resin (Qiagen) and subsequent size exclusion chromatography on a HiLoad 16/600 Superdex 75 prep grade column (GE Healthcare) in 50 mM Tris (pH 7.5), 200 mM NaCl, 5 mM BME, 1 mM EDTA, and 0.05% Tween 20. Purified CK1 proteins used for in vitro kinase assays were run on size-exclusion columns or buffer exchanged into storage buffer (50 mM Tris (pH 7.5), 100 mM NaCl, 1 mM TCEP, 1 mM EDTA, and 10% glycerol) using an Amicron Ultra centrifugal filter (Millipore) and frozen as small aliquots in liquid nitrogen for storage at −80°C.

For human PER2 FASP peptide preps, cells were lysed in a buffer containing 50 mM Tris (pH 7.5), 500 mM NaCl, 2 mM TCEP, 5% glycerol, and 25 mM imidazole using a high-pressure extruder (Avestin) or sonicator on ice (Fisher Scientific). His-NusA-FASP fusion proteins were purified using Ni-NTA resin using standard approaches and eluted from the resin using 50 mM Tris (pH 7.5), 500 mM NaCl, 2 mM TCEP, 5% glycerol, and 250 mM imidazole. His-TEV protease was added to cleave the His-NusA tag from the PER2 peptides at 4°C overnight. The cleavage reaction was subsequently concentrated and desalted into low imidazole lysis buffer using a HiPrep 26/10 Desalting column. Peptides were purified away from His-NusA and His-TEV using Ni-NTA resin with 50 mM Tris (pH 7.5), 500 mM NaCl, 2 mM TCEP, 5% glycerol, and 25 mM imidazole. Peptides were purified by size-exclusion chromatography on a HiLoad 16/600 Superdex 75 prep grade column, using NMR buffer (25 mM MES (pH 6.0), 50 mM NaCl, 2 mM TCEP, and 10 mM MgCl_2_) or 1× kinase buffer (25 mM Tris (pH 7.5), 100 mM NaCl, 10 mM MgCl_2_, and 2 mM TCEP) for NMR or ADP-Glo kinase assays, respectively.

### NMR kinase assays

NMR spectra were collected on a Varian INOVA 600 MHz or a Bruker 800 MHz spectrometer equipped with a ^1^H, ^13^C, ^15^N triple resonance z-axis pulsed-field-gradient cryoprobe. Spectra were processed using NMRPipe (107) and analyzed using CCPNmr Analysis (108). Backbone resonance assignments were determined previously (47). NMR kinase reactions were performed at 25°C with 150 μM ^15^N-human PER2 FASP, 2.5 mM ATP, and 2 μM CK1. SOFAST HMQC spectra (data acquisition = 5 min) were collected at the indicated timepoints, or HSQC spectra were collected on quenched samples (after addition of EDTA to final concentration of 20 mM) at the indicated timepoints, and the relative peak volumes were calculated and normalized as described previously (25).

### ADP-Glo kinase assays

Kinase reactions were performed on the indicated recombinant peptides (FASP WT or alanine mutants) using the ADP-Glo kinase assay kit (Promega) according to manufacturer’s instructions. All reactions were performed in 30 μL volumes using 1× kinase buffer (25 mM Tris (pH 7.5), 100 mM NaCl, 10 mM MgCl_2_, and 2 mM TCEP) supplemented with ATP and substrate peptides. To determine apparent kinetic parameters (K_M_), duplicate reactions with 100 μM ATP and 200 nM CK1 kinase were incubated in 1× kinase buffer at room temperature for 1 h with the indicated amount of substrate peptide (and repeated for *n* = 2 independent assays). Five-microliter aliquots were taken and quenched with ADP-Glo reagent after the 1 h incubation, and luminescence measurements were taken at room temperature with a SYNERGY2 microplate reader (BioTek) in 384-well microplates. Data analysis was performed using Excel (Microsoft) or Prism (GraphPad).

### Radioactive kinase assays

PER FASP region peptides were synthesized and purified to 95% or higher (SABio). Two independent reaction mixtures of 50 μL containing 200 μM of the FASP or in reaction buffer (25 mM Tris (pH 7.5), 7.5 mM MgCl_2_, 1 mM DTT, and 0.1 mg/mL BSA) were preincubated for 5 min with or without 20 nM CK1 (for primed FASP) or 200 nM CK1 (for unprimed FASP) and the reaction was started by addition of 750 μM of UltraPure ATP (Promega) containing 1–2 μCi of ɣ-^32^p ATP (PerkinElmer). After incubation of the reaction mix at 30°C, an 8-μL aliquot of the reaction mix was transferred to P81 phosphocellulose paper (Reaction Biology Corp) at the indicated timepoints. The P81 paper was washed three times with 75 mM of orthophosphoric acid and once with acetone. The air-dried P81 paper was counted for P_i_ incorporation using a scintillation counter (PerkinElmer) by Cherenkov counting. Results shown are from four independent assays.

**Table:** 

Peptide Name	Species	Peptide Sequence	Figure Shown
PER2 WT (FASP)	mouse	RKKKTEVSAHLSSLTLP GKAESVVSLTSQ	Fig. S15*B*
PER2 pS659 (primed FASP)	mouse	RKKKTEVSAHLSSLTL PGKAEpSVVSLTSQ	Fig. S15*C*
PER3 WT	mouse	RKKKPSTDIEGGAAR TLSTAALSVASGISQ	Fig. S15*E*
PER3 KAE	mouse	RKKKPSTDIEGGAART LSTKAESVASGISQ	Fig. S15*E*
PER3 WT (priming only)	mouse	RKKKPSTDIEGGAAR TLSTAALSVAAGIAQ	Fig. S15*F*
PER3 KAE (priming only)	mouse	RKKKPSTDIEGGAAR TLSTKAESVAAGIAQ	Fig. S15*F*

## Results

### Mapping the conformational landscape of CK1

#### The activation loop and loop EF are the slowest loops in CK1

To characterize the dynamics of WT CK1 and the *tau* mutant, we ran multiple all-atom MD simulations totaling 21 μs for each protein system (Fig. S1). We then employed the MSM framework to integrate these trajectories into a single model describing the conformational free energy landscape of CK1 in WT and *tau*. MSMs can derive long timescale dynamics from a large number of relatively short MD simulations by 1) dividing the conformational space into a large number of discrete microstates, 2) using the MD data to count transitions between states after a specified lag time, and finally, 3) estimating a transition matrix that describes the dynamics of the system in the discretized conformational space (76,109). An important step in model building thus consists in selecting relevant features to discretize the conformational space. For our model, we started by selecting pairwise distances involving functionally important regions of CK1 and applied time-lagged independent component analysis (tICA) (110) to reduce these features to a smaller number of collective variables representing the slowest modes of motions (TICs). We found that the slowest and dominant mode of motion in CK1 involves the activation loop (TIC 1) and that interconversion between the “loop up” and “loop down” conformations happens at long scales, elucidating an important regulatory role for this loop (Fig. S2). The second slowest motion (TIC 2) involves L-EF, near the first anion site, which has been previously implicated in substrate binding and temperature-compensation mechanisms (31,40,45). Based on these results, we selected five pairwise distances that were jointly combined by tICA to create the final MSMs. Further methodological details on construction of the models are provided in supplemental materials and methods (see section Markovian state models*;* Figs. S3–S5).

#### Tau accelerates and inverts the conformational equilibrium involving the activation loop

**Figure 2:**
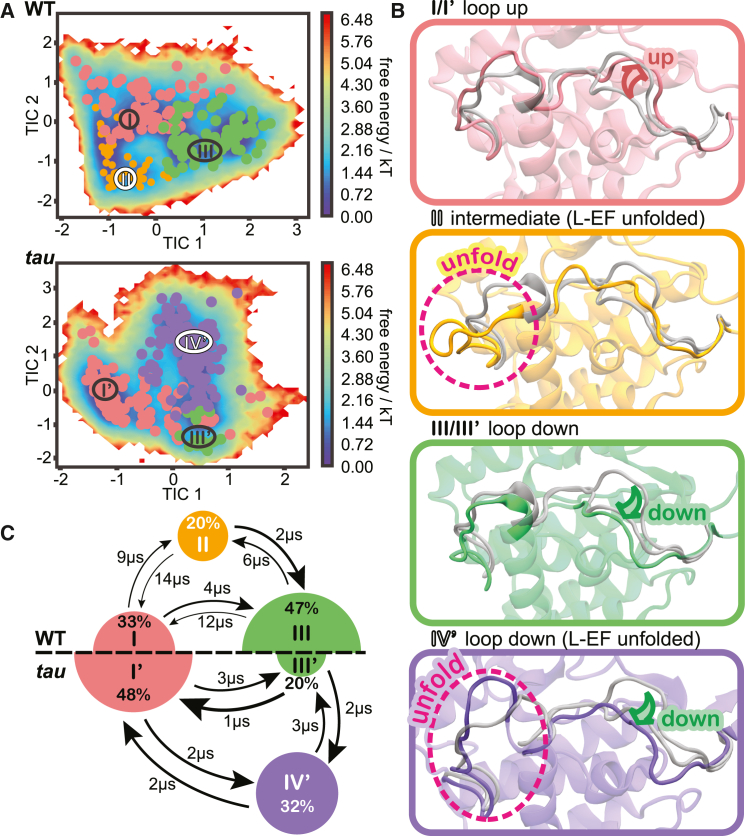
The equilibrium between three preferred conformational states is accelerated and inverted by the CK1 *tau* mutant. (*A*) Free energy landscapes of the WT CK1 (*top*) and *tau* mutant (*bottom*) in terms of the slowest tICA components, with microstates clustered into metastable states (*identified by roman numerals*). (*B*) Representative conformations of each metastable state. For comparison, x-ray conformations of the activation loop and L-EF are superimposed (*transparent gray*; loop down PDB: 1CKJ, loop up PDB: 6PXN). (*C*) Equilibrium populations of metastable states and MFPTs between states for the WT (*top*) and *tau* (*bottom*) protein systems. Radius of the circles is proportional to the equilibrium population percentage, and thickness of the arrows is proportional to transition rates between states. The numbers next to the arrows indicate MFPTs.

MSM-based conformational landscapes for WT CK1 and the *tau* mutant reveal that each system displays three preferred states (Fig. 2 *A*). Two of these states are roughly equivalent in WT and *tau* (states I/I’ and III/III’), whereas states II and IV’ are exclusive of WT and *tau*, respectively. Visual inspection supported by pairwise distances and RMSD (Fig. S6) reveal that the states differ mainly with respect to the conformation of the activation loop (up, down, or intermediate) and the conformational state of L-EF (folded or unfolded) (Fig. 2 *B*). States I/I’ and III/III’ correspond to “loop up” and “loop down” conformations of the activation loop, respectively, in relatively good agreement with corresponding crystallographic structures. The remaining states display the L-EF in an unfolded state, with the activation loop either adopting an intermediate conformation (state II, in WT) or the “loop down” conformation (state IV,’ in *tau*).

Populations derived from the MSM uncover a slow equilibrium between “loop up” (state I) and “loop down” (state III) conformations in the WT kinase, with a clear preference for the “loop down” conformation (Fig. 2 *C*, top). The observation of an additional metastable state (II) in which the activation loop adopts a “halfway” conformation suggests the existence of an intermediate state connecting the two main states (I and III) in the WT enzyme. This intermediate state also suggests that flipping of the activation loop requires disorganization of L-EF, which displays significantly more conformational freedom in state II (Fig. S6).

Interestingly, the *tau* mutation inverts the conformational equilibrium characteristic of WT, stabilizing the activation loop in the “up” conformation (state I’) and destabilizing the “down” conformation (Fig. 2 *C*, bottom), which is almost always accompanied by unfolding of L-EF (state IV’). In addition, flipping of the activation loop between up and down conformations does not involve a long-lived intermediate or “midway” conformation. Such inversion of the conformational landscape strongly supports the activation loop as a key molecular switch controlling the circadian period since the *tau* mutant is known for inverting CK1 selectivity for its circadian substrates. Our model also recapitulates previous findings that the *tau* mutation destabilizes the first anion site, facilitating the unfolding of L-EF, but “only” when the activation loop is down (state IV’) (73). When the activation loop is up, L-EF remains folded and displays WT-like dynamics (state I’), supporting that the activation loop exerts allosteric control over the dynamics of L-EF.

Apart from equilibrium populations, MSMs also provide transition rates between states, informing on the kinetics of the system. By comparing mean first passage times (MFPTs) between states (Fig. 2 *C*, arrows), we find that the *tau* mutation significantly accelerates the conformational transitions between states. Interconversion between loop up and down conformations in the *tau* mutant (I’ ⬄ IV’ ≈ 2 μs) is at least two times faster than in the WT enzyme (I ⬄ III ≈ 4–12 μs), indicating that the *tau* mutation significantly reduces the energy barriers associated with flipping of the activation loop. This agrees with the lack of intermediate “midway” conformations in the *tau* mutant. *Tau* also accelerates unfolding of L-EF, which in state IV’ adopts a wide range of conformations ranging from collapsed to fully extended (see Fig. S6 *B*).

#### The role of Gly^175^ in the conformational dynamics of the activation loop

**Figure 3:**
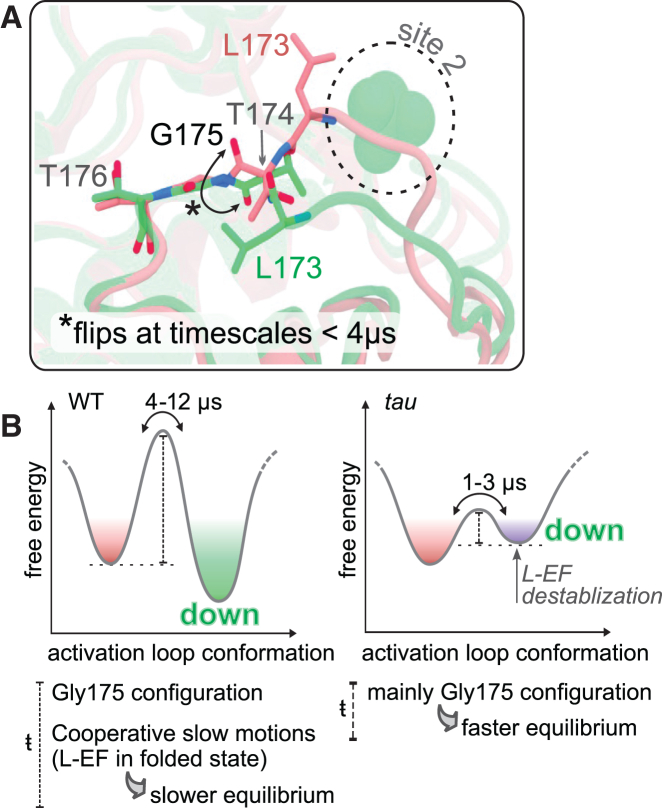
The role of Gly^175^ for the conformational dynamics of the activation loop. (*A*) View of x-ray structures highlighting the different configurations adopted by Gly^175^ in “loop up” and “loop down” conformations of the CK1 activation loop (PDB: 6PXN and 1CKJ, respectively). (*B*) Schematic representation of the conformational landscape involving the activation loop, based on MSM-derived kinetics and stability of the two most populated states in each system (WT CK1, *top*; and *tau*, *bottom*).

The activation loop in CK1 is preceded by a conserved glycine at position 175, distinct from the glycine found in the classic DFG motif (Gly 151) that flips between catalytically active and inactive conformations. In other serine/threonine kinases, a backbone flip of a glycine at this conserved position may be correlated with conformational changes of the activation loop, and therefore, it could link these loop conformations to the first anion binding pocket *tau* mutation site (R178C) (111). In CK1, x-ray structures suggested that Gly^175^ backbone could work as a “hinge” controlling the conformation of the activation loop (Fig. 3 *A*) (40,73). To investigate this hypothesis, we built MSMs based solely on the backbone angles of Gly^175^ (Figs. S7 and S8).

The resulting Gly^175^-based MSMs revealed no significant correlation between the configuration of Gly^175^ and the conformation of the activation loop in WT CK1 (Fig. S9). Thus, although the configuration of Gly^175^ might determine the most energetically stable conformation of the activation loop in low entropy scenarios (crystalline state), its importance appears to be overcome by other factors when the protein is in solution. Surprisingly, the *tau* mutant displays a moderate correlation between φ^Gly175^ and the activation loop (φ^Gly175^ < 0° favors “loop up” and φ^Gly175^ > 0° favors “loop down”), indicating that Gly^175^ backbone is more determinant of the conformation of the activation loop in the *tau* mutant than in the WT enzyme.

In part, this can be explained by the energetic barriers separating states in the conformational landscape of CK1. In the WT enzyme, a configurational torsion of Gly^175^ alone is not enough to overcome the high energy barriers associated with the flip of the activation loop. In the *tau* mutant, these barriers are decreased by disruption of the first anion binding site and unfolding of L-EF, allowing the conformational dynamics of the activation loop to be influenced more heavily by the backbone configuration of Gly^175^. This is in excellent agreement with MFPTs provided by the MSMs that show that torsional transitions of Gly^175^ happen at timescales faster than 4 μs (Fig. S8 *A*), the same timescale range at which the activation loop flips in the *tau* mutant (see Fig. 2 *C*). In WT CK1, loop transitions happen at much longer timescales (4–12 μs) (see Fig. 2 *C*), indicating that the conformational landscape in the WT enzyme is governed by slow cooperative motions, likely related to the conformational state of L-EF (Fig. 3 *B*).

### A molecular model of FASP interactions involved in priming

**Figure 4:**
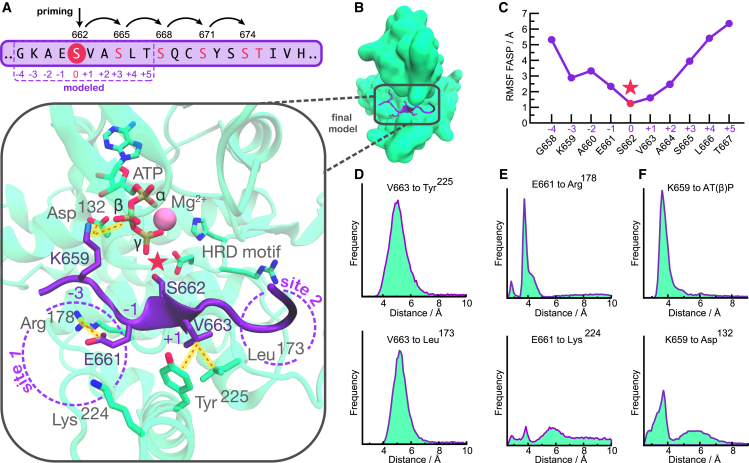
Molecular model of the interaction between CK1 and unphosphorylated FASP. (*A*) The FASP region of human PER2. Residue numbers with arched arrows represent serines that are sequentially phosphorylated by CK1. Positions listed below (−4 to +5) are relative to priming serine, S662. (*B*) Representative structure of the final model with the unphosphorylated FASP peptide (*purple*) and CK1 (*green*). For clarity, we use one-letter amino acid code for residues of the substrate (FASP) and three-letter code for residues of the kinase CK1. Pink star represents the priming event of S662. (*C*) Atomic fluctuations of the bound FASP based on accumulated molecular dynamics trajectories. (*D–F*) Distance-based interaction histograms involving V663 at position +1 (*D*), E661 at position −1 (*E*), and K659 at position −3 (*F*) with CK1 residues or ATP.

To gain a better understanding on how the conformational landscape of CK1 is linked to its activity on circadian substrates, we decided to model the interaction between CK1 and the FASP region of PER2 (Fig. 4 *A*). We based our initial model on a recently published crystallographic structure of CK1 bound to TAp63α (Fig. S10, PDB: 6RU7, chains A and C) (75). As with FASP, TAp63α also undergoes sequential phosphorylation by CK1, with the difference that the priming is achieved by another kinase, CDK2 (112). We refined our model with a combined set of 2 μs of accumulated GaMD simulations followed by additional 2 μs of accumulated cMD simulations (Fig. S11, details in supporting material).

#### FASP binds to the loop down conformation

Our final model consists of a FASP-bound conformation in which the priming serine (S662 in human PER2) is well positioned to accept the γ phosphate from ATP, with the activation loop in the “down” conformation (Figs. 4 *B* and S11 *B*). V663 (at position +1) appears to play a key role in anchoring the backbone of FASP into the active site (see Fig. 4 *B*), displaying low atomic fluctuations (Fig. 4 *C*) and engaging in persistent hydrophobic interactions with Tyr^225^ in helix αF and with Leu^173^ in the activation loop (Fig. 4 *D*).

#### Electrostatic interactions upstream of the priming site of FASP contribute to substrate binding

Although residues downstream of the priming site display high mobility freedom and a lack of persistent interactions with CK1, the upstream region of FASP is less dynamic and likely to contribute to substrate binding (Fig. 4 *C*). We noticed that the first anion binding site is partially occupied by E661 (at position −1), which displays persistent electrostatic interactions with Arg^178^ but not as much with Lys^224^ (Fig. 4 *E*). In the *tau* mutant, Arg^178^ is replaced with a cysteine, likely disrupting this interaction. We also found that K659 (at position −3) invariably forms a salt bridge with the β phosphate of ATP, oftentimes assisted by an additional salt bridge with an Asp^132^ (just downstream of the catalytic HRD motif) (Fig. 4 *F*).

### Biochemical validation of the PER FASP priming model

**Figure 5:**
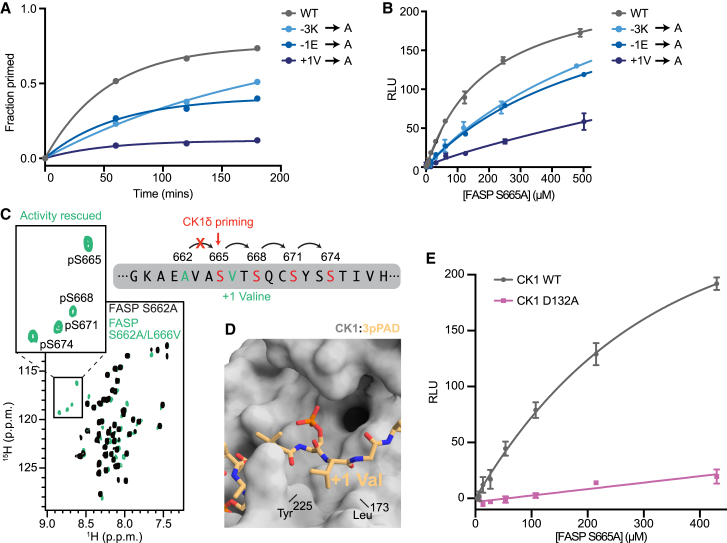
Biochemical validation of the model for nonconsensus FASP priming. (*A*) NMR-based time course kinase assay quantifying the increase in peak volume corresponding to the phosphopeak of the priming serine in human PER2 FASP (S662), taken from a series of ^15^N-^1^H HSQC spectra (Fig. S14). (*B*) Representative ADP-Glo titration assay comparing WT FASP and alanine mutants at positions −3, −1, and +1 in the “priming only” background (S665A mutation to halt sequential kinase activity; see Fig. S15*A*) from replicate titration experiments *n* = 2, mean ± standard deviation. (*C*) ^15^N-^1^H HSQC spectra comparing a “priming disrupted” FASP peptide (S662A, *black*) and a “priming rescued” FASP peptide (S662A/L666V, *teal*) in the presence of CK1. Zoom shows the region of the spectra where phosphoserines appear. (*D*) Zoom of co-crystal structure of CK1 and a triply phosphorylated Tap63α PAD peptide, 3pPAD, highlighting the active site and +1 hydrophobic pocket region (PDB: 6RU8). The +1 valine residue is inserted between the small hydrophobic pocket created between Tyr^225^ and Leu^173^ of CK1 when the activation loop is in the downward conformation. (*E*) ADP-Glo titration of the “priming only” (S665A) FASP peptide comparing CK1 WT (*black*) and D132A (*purple*) from replicate titration experiments *n* = 2, mean ± standard deviation.

To support our model of unprimed FASP bound to CK1, we performed a series of biochemical experiments to assess the relative contribution of residues near the FASP priming site as molecular determinants of CK1 priming activity. As we have done previously (25,47,73), we used an NMR-based kinase assay to measure priming activity within human PER2 FASP peptides with site-specific resolution. In agreement with our model, introducing alanine mutations to the −3, −1, or +1 positions of FASP led to a decrease in priming activity (Figs. 5 *A* and S14).

To get a further sense of how these mutations might contribute to the binding of unprimed FASP, we introduced an alanine mutation at the +4 Ser (S665) to limit kinase activity to just the priming site (Fig. S15 *A*). We then performed substrate titrations of FASP peptides with alanine mutations at the −3, −1, and +1 positions in this “priming only” (S665A) background by ADP-Glo assay. Similar to the NMR assay, the −3 and −1 alanine mutations led to an approximate 10-fold increase in the apparent Michaelis-Menten constant (K_M_app) relative to the WT substrate (K_M_app = 210.3 ± 0.3 μM); the K_M_app for +1 alanine mutant could not determined due to its low activity. Together, these values suggest significantly reduced affinity of the unprimed mutant FASP substrates for the kinase (Fig. 5 *B*). Mutation of the +1 valine in FASP had the largest decrease in kinase activity, in agreement with lower overall atomic fluctuations displayed by this residue in our MD model (Fig. 4 *C*). Moreover, we found that priming activity can be rescued at the downstream serine in a “priming deficient” S662A mutant by simply substituting a valine for the +1 leucine at the second phosphorylation site (i.e., FASP S662A/L666V, Fig. 5 *C*). Taken together, these results indicate that priming of FASP is highly sensitive to the presence of a valine at the +1 position. Interestingly, the hydrophobic pocket occupied by this valine is created between Tyr^225^ and Leu^173^ of CK1 only when the activation loop is down. The +1 hydrophobic pocket is also small and likely contributes to substrate selectivity based on steric occlusion of bulkier residues at the +1 position, considering that co-crystal structures of CK1 bound to phosphorylated Tap63α PAD and FASP peptides are composed entirely of backbone-backbone interactions between peptide and kinase in this region (Fig. 5 *D*).

We further tested our FASP binding model by introducing an alanine mutation to CK1 at Asp^132^ (D132A) because this residue frequently interacted with the −3 lysine of FASP over the course of the MD trajectories. Titration of the “priming only” FASP peptide (S665A) comparing CK1 WT and D132A showed a dramatic loss of kinase activity (Fig. 5 *E*). Since Asp^132^ is located directly under the nucleotide binding site and potentially in position to contact ATP, we also sought to test whether the D132A mutation would disrupt kinase activity on a primed FASP peptide, where CK1 activity is driven by the consensus recognition mechanism involving the first anion binding site. Although the D132A mutation showed a dramatic loss in activity on the unprimed FASP peptide, it had a more modest effect on the primed FASP peptide (Fig. S15 *B* and *C*), suggesting that this mutation primarily reduces activity on FASP via recognition of the unprimed substrate.

We showed that CK1 phosphorylates the nearly identical PER1 FASP sequence comparable to PER2, both in vitro and in cells (47). However, one PER homolog within the circadian system, PER3, does not appear to be a substrate of the kinase and lacks the critical residues that precede the priming serine (113). Here, we further tested our model by introducing a lysine at the −3 position and a glutamate at the −1 position of PER3 FASP-like peptides (A610K/L612E) to mimic the PER2 FASP priming region and rescue priming activity. These mutant peptides were used in a ^32^P-ATP time course kinase assay and showed an increase in CK1 activity for both the WT (Fig. S15 *E*) and the “priming only” (Fig. S15 *F*) substrates in the presence of a −3K and −1E, further demonstrating that these residues play an important role in CK1 recognition and activity on FASP substrates. An alignment comparing these CK1 motifs with other known CK1-targeted poly-SXXS motifs shows that the −3K, −1E, and +1V seem to be conserved just among the PER substrates (Fig. S16).

### Mapping of binding pockets on the CK1 surface

Our MSMs support that inversion of substrate selectivity in the *tau* mutant is achieved by inversion of the conformational equilibrium in the activation loop, with destabilization of the “loop down” conformation in favor of the alternative “loop up.” To understand how the activation loop reshapes the molecular surface of CK1 and to look for potential binding sites controlling substrate selectivity, we screened the CK1 surface using FTMap (105). For more details, see supplemental materials and methods, Section E.

#### The activation loop significantly reshapes the substrate binding cleft in CK1

**Figure 6:**
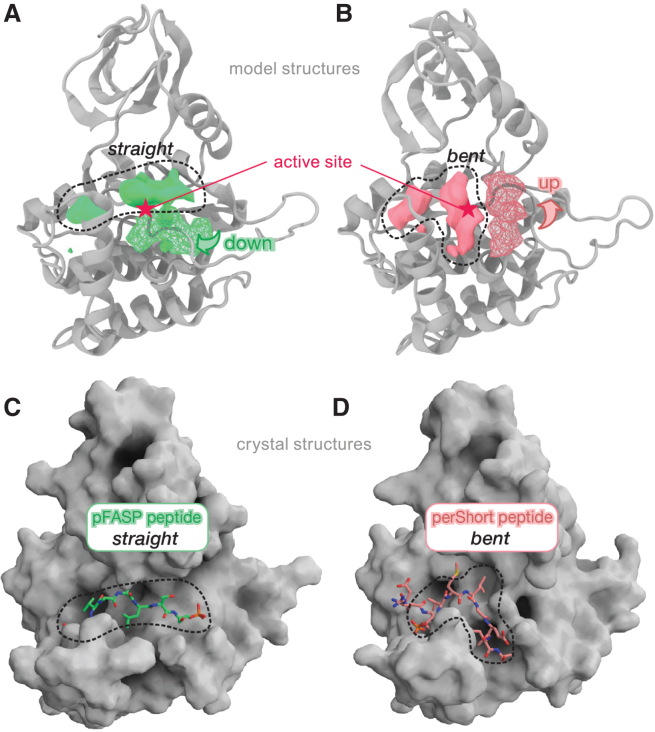
The conformation of the activation loop shapes the substrate binding cleft. (*A*) The “loop down” conformation of the activation loop creates a straight binding cleft, whereas (*B*) the “loop up” conformation creates a bent binding cleft, opening a subpocket right under the active site. The solid blobs represent density maps computed based on organic probes from FTMap, whereas the meshed blobs represent occupancy maps computed for residues Lys^171^, Asn^172^, and Leu^173^ along the simulations. (*C* and *D*) Co-crystal structure of human PER2 pFASP bound to CK1 (PDB: 8D7M) in the straight substrate binding cleft (*C*), whereas the dPER perShort peptide binds to CK1 (PDB: 8D7P) following a bent substrate binding cleft (*D*).

For both the WT and *tau* mutant, the top-ranked pockets identified by FTMap correspond to the ATP-binding site and the Mg^2+^ pocket near the catalytic site (Tables S7–S10). Interestingly, we observed a fragmentation of the active site and substrate binding cleft in the *tau* mutant, which break down into three separate subpockets not as well ranked as the large contiguous pocket detected in the WT enzyme (Figs. S16 and S17). Comparison of these pockets when CK1 is in “up” or “down” conformation reveal how dramatically the activation loop conformation reshapes the substrate binding cleft (Fig. 6). The “loop down” conformation promotes a straight binding cleft running contiguously from the first to the second anion binding site, above the activation loop (Fig. 6 *A*). The “loop up” conformation, however, appears to promote a bent binding cleft, with part of the substrate channel in the space between helices αD and αF, below the activation loop (Fig. 6 *B*). Considering that the WT CK1 has a higher preference for FASP, we hypothesized that the straight binding cleft produced by the more common “loop down” conformation is well suited to bind the FASP substrate, in agreement with our recent co-crystal structures of WT CK1 bound to phosphorylated FASP peptides (pFASP) (Fig. 6 *C*) (47). The bent binding cleft produced by “loop up” conformations could more favorably interact with the pD substrate, which could explain how *tau* not only reduces activity on FASP but also increases the activity on pD substrates. Interestingly, we found that a *Drosophila* PER (dPER) peptide phosphorylated at S589 (perShort peptide), the site of the *per*^S^ mutation that destabilizes dPER and shortens circadian period (114), binds to CK1 when the substrate binding cleft is bent (47). The pS589 of the perShort peptide coordinates the first anion binding site identically to pFASP but follows a channel exposed by the “loop up” conformation of the activation loop (Fig. 6 *D*), in excellent agreement with the FTMap analysis. We did not submit the intermediate state of the activation loop (state II) in this analysis because, due to the high level of disorder in the L-EF region observed in this state, we do not expect it to be able to hold substrates in the binding cleft.

#### A binding pocket to shorten the circadian rhythm

**Figure 7:**
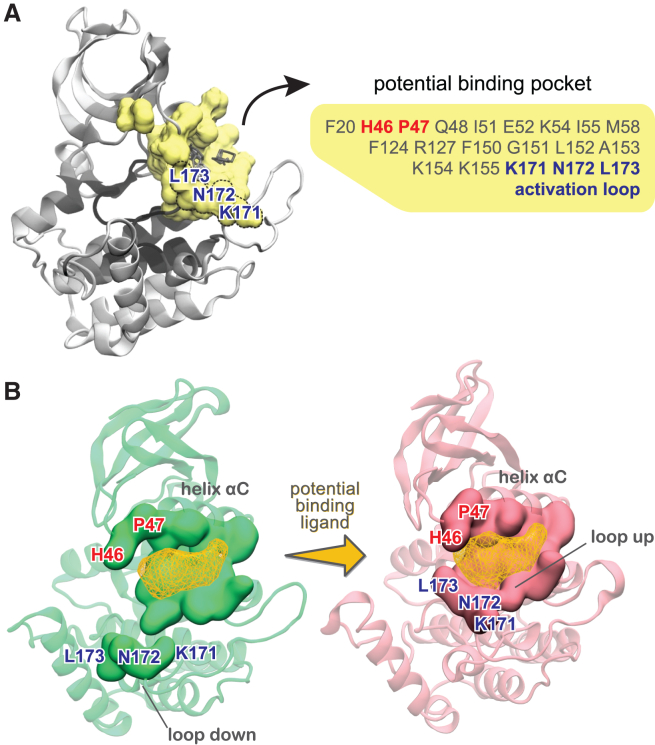
A potential binding pocket to shorten the circadian rhythm. (*A*) Residues belonging to the *tau* activation pocket are highlighted in yellow, whereas the small organic molecules used as probes by FTMap are represented as gray sticks. (*B*) Complete assembly of this pocket occurs when the activation loop is “up.” Ligand binding to this pocket is likely to stabilize the “loop up” conformation.

FTMap also identified potential binding sites on the surface of CK1, as described in supplemental materials and methods, Section E. Of particular interest is a pocket formed between helix αC and the activation loop (Fig. 7 *A*). As highlighted in Fig. 7 *A*, this “activation” pocket includes three residues belonging to the activation loop (Lys^171^, Asn^172^, and Leu^173^) and it is only fully assembled when the activation loop is up. Indeed, this pocket is ranked higher in the *tau* mutant (Table S10) than in the WT enzyme (Table S9), in agreement with the fact that *tau* stabilizes the upward conformation of the activation loop. Other key residues forming this potential binding pocket are His^46^ and Pro^47^ located in the loop connecting sheet β3 to helix αC (Fig. 7 *B*). Interestingly, mutations at these positions (H46R and P47S) of the *Drosophila* CK1 ortholog, *Doubletime,* shorten the circadian period by ∼4 h (62). Thus, occupation of this binding pocket by small molecules mimicking the sidechains of serine and arginine could stabilize the “loop up” conformation and bend the substrate binding cleft. This could increase CK1 activity against the pD and ultimately shorten the circadian period. A comparison of this potential CK1 pocket to known targeted pockets on several other kinase domains is provided in Fig. S19.

## Discussion

Despite its central role in governing circadian rhythms, the molecular mechanism underlying CK1 selectivity for its target regions of the circadian protein PER remains poorly understood, hindering rational attempts to develop CK1-specific circadian drugs. CK1’s active site, activation loop, and three anion binding sites are used to bind phosphorylated peptides (46,47) and are extremely well conserved across eukaryotes (73). However, because CK1 is generally thought to be constitutively active toward primed or acidic substrates (34,40,41,42,45), the role of its activation loop has been largely unexplored. Efforts to characterize serine/threonine kinase specificity in relation to substrate sequence variation have typically identified just the high-activity, primed consensus motif for CK1 (115). Additionally, many CK1 substrates with multiserine clusters motifs are initiated by priming phosphorylation from another kinase. However, CK1 activity on circadian substrates is distinctive because it acts on slower, nonconsensus phosphorylated motifs (25,47,116). To determine the molecular basis of CK1 activity specifically at these nonconsensus motifs, we used an extensive set of MD simulations integrated into MSMs to map the free energy landscape governing the slow conformational transitions of CK1’s activation loop. Our model revealed the existence of a slow equilibrium between the more stable “loop down” and the less stable “loop up” conformations in the WT enzyme and showed that the period-shortening *tau* mutant biases and accelerates the transitions toward the “up” conformation.

We also found that the dynamics of the activation loop are strongly connected to the conformational state of L-EF and that flipping of the activation loop requires transient unfolding of L-EF. This explains the long timescales associated with “up-down” transitions, which would happen faster if the conformation of the activation loop was controlled mainly by the configuration of Gly^175^, as previously hypothesized (73). Elucidation of the role of Gly^175^ for the conformational dynamics of the activation loop reinforces the importance of MD in re-visiting and adding complexity to structural hypotheses that are based on static observations. These findings also explain how the R178C *tau* mutation, which facilitates the unfolding of L-EF by disrupting the first anion binding site, significantly accelerates the dynamics of the activation loop. Interestingly, given the importance of L-EF for temperature-compensation against circadian substrates (31,40,45) and its allosteric connection with the activation loop (47), it is likely that the dynamic equilibrium involving the activation loop is also part of CK1 temperature-compensation mechanisms.

This work characterizes for the first time to our knowledge how CK1 interacts with the unprimed PER FASP region. Our model of the FASP interaction with CK1 suggests key interactions important for the nonconsensus priming step that are supported by in vitro biochemistry. In agreement with the relatively low atomic fluctuations of the +1 residue (relative to the priming site) from our model, our data clearly demonstrate the importance of the +1 valine (+1V) for nonconsensus priming of FASP, which fits into a small hydrophobic pocket created between Leu^173^ and Tyr^225^ on CK1. Our model also reveals that FASP makes use of the first anion binding site by partially inserting a negatively charged glutamate at position −1 (−1E) of the substrate. Additionally, we showed that stabilization of FASP in the binding cleft is complemented by electrostatic interactions between the lysine at position −3 (−3K) with Asp^132^ of CK1 as well as the β-phosphate group of ATP. Finally, we demonstrated that addition of a −3K and −1E to PER3 was sufficient to allow phosphorylation of the priming serine by CK1. Together these findings establish −3K, −1E, and +1V as bona fide residues necessary for proper CK1 recognition of FASP as a substrate.

Interestingly, although the −3K-ATP interaction can lock the FASP substrate in place for the priming event, we speculate that this interaction could also facilitate unbinding (or translocation) of the primed product by attaching it to the leaving ADP subproduct. In addition, substrate stabilization achieved by partial occupation of the first anion site by −1E could be just enough to promote priming without excessive stabilization of the primed product. In consensus substrates, this anion site is fully occupied by a negatively/phosphorylated residue at position −3, as recently shown in the structures of CK1 bound to primed Tap63a or PER FASP peptides (47,75). It can even be double occupied (by phosphorylated serines at positions −3 and −6) in the case of multiply phosphorylated Tap63α (75), or as seen in one crystal structures of CK1 in anionic solvent conditions (31). This suggests that the partial occupation of the first anion site by unprimed FASP is likely to be replaced with progressively stronger occupation of this site by a phosphorylated serine at position −3 to allow subsequent phosphorylation events. In a processive or semiprocessive mechanism, these escalating electrostatic interactions at the upstream region of FASP would provide a direction for the sequential phosphorylation events, whereas the lack of specific interactions in the downstream region would make it easier for the next phosphorylation site to translocate into the active site.

Using FTMap to map the surface of CK1 in different conformations, we demonstrated how the conformation of the activation loop reshapes the substrate binding cleft. Applying FTMap to the conformations extracted from our MSM also allowed us to identify a potentially attractive binding site between helix αC and the activation loop. Interestingly, this site partially overlaps with the site that accommodates a phosphate group in kinases that are activated by phosphorylation of the activation loop (73), suggesting a possible regulatory role. To add to that, two period-shortening mutations are found in this site: P47S and H46R. Differently from *tau*, both P47S and H46R mutants achieve their period-shortening effects by increasing activity on the pD region while retaining normal FASP priming activity relative to WT (73). This is consistent with what would be expected from increasing the population of “loop up” and “bent” substrate binding cleft conformations (which favor pD phosphorylation) without disruption of the first anion binding pocket (important for priming and subsequent phosphorylation of FASP). Targeting this pocket could help to bias the conformation of CK1 and have predictable effects on circadian period. Because this pocket is only fully assembled when the activation loop is “up,” we propose that it might be a viable site to pharmacologically stabilize the “up” conformation and shorten the period of circadian rhythms.

## Conclusion

Because PER stability is directly linked to clock periodicity, maintaining an ∼24-h circadian rhythm requires a delicate balance between its two antagonistic CK1 phosphorylation targets: the FASP and degron regions. CK1 balances its activity against these two substrates by using its activation loop as a molecular switch, forming specific binding cleft conformations when the loop is either up or down. This switch from “loop up” and “loop down” is controlled by a slow equilibrium dependent on the activation loop’s allosteric connection to loop EF and the anion binding sites. The circadian mutant *tau* accelerates the dynamics between the activation loop conformational states and stabilizes the “loop up” position, biasing kinase activity toward the degron that results in a shorten circadian period. *Tau* exemplifies the necessity for steep energetic barriers between the “loop up” and “loop down” conformations to control proper circadian timekeeping. Additionally, CK1 recognizes the FASP substrate lacking a consensus motif by using conserved residues surrounding the priming serine. This forms the molecular basis for the critical slow, rate-limiting priming step within the PER phosphoswitch mechanism. Finally, this work identifies a potential CK1 binding site that could be targeted to stabilize the activation “loop up” conformation to modulate phosphorylation activity toward the degron, expanding our therapeutic development strategies to fine-tune circadian period.

## Data and code availability

Jupyter notebooks and data for Markov state models are provided at GitHub/cpartch/CK1. Instructions for running simulations can be found in the supporting material.

## Acknowledgments

Funding for this work was provided by the 10.13039/100000002US National Institutes of Health grant R35 GM141849 (C.L.P.). D.M.V. was supported by Singapore Ministry of Health grant MOH-000600. J.A.M. was supported by TSCC computer resources from UC San Diego. C.L.P. was supported by the 10.13039/100000011Howard Hughes Medical Institute.

## Author contributions

C.G.R. and J.M.P. designed research, performed experiments, analyzed data, and wrote the manuscript. M.R.T. prepared materials, performed experiments, and edited the manuscript. A.M.F. and R.N. prepared materials, performed experiments, and analyzed data. E.P. performed experiments and analyzed data. R.A., D.M.V., J.A.M., and C.L.P. guided research, funded research, and edited the manuscript.

## Declaration of interests

The authors do not declare any conflicts of interest.

## Supporting material

Document S1. Figures S1–S19 and Tables S1–S10

Document S2. Article plus supporting material
