# Supplementary material for: Markovian state models uncover casein kinase 1 dynamics that govern circadian period
Source: Biophys J. 2025 Sep 18;124(22):4034–48. doi: 10.1016/j.bpj.2025.09.022 (PMC12709424; doi:10.1016/j.bpj.2025.09.022)
Supplement: Document S2. Article plus supporting material [file mmc2.pdf]

Biophysical Journal
Article

Markovian state models uncover casein kinase 1 dynamics that govern circadian period

Clarisse Gravina Ricci,[1,*] Jonathan M. Philpott,[2] Megan R. Torgrimson,[2] Alfred M. Freeberg,[2] Rajesh Narasimamurthy,[3] Emilia Pécora de Barros,[1] Rommie Amaro,[1] David M. Virshup,[3,4] J. Andrew McCammon,[1] and Carrie L. Partch[2,5,6,*]

[1]Department of Chemistry and Biochemistry, University of California, San Diego, San Diego, California; [2]Department of Chemistry and Biochemistry, University of California, Santa Cruz, Santa Cruz, California; [3]Program in Cancer and Stem Cell Biology, Duke-NUS Medical School, Singapore, Singapore; [4]Department of Pediatrics, Duke University Medical Center, Durham, North Carolina; [5]Center for Circadian Biology, University of California, San Diego, San Diego, California; and [6]Howard Hughes Medical Institute, University of California, Santa Cruz, Santa Cruz, California

SUMMARY Circadian rhythms in mammals are tightly regulated through phosphorylation of period (PER) proteins by casein kinase 1 (CK1, subtypes δ and ε). CK1 acts on at least two different regions of PER with opposing effects: phosphorylation of phosphodegron regions leads to PER degradation, whereas phosphorylation of the familial advanced sleep phase (FASP) region leads to PER stabilization. To investigate how substrate selectivity is encoded by the conformational dynamics of CK1, we performed a large set of independent molecular dynamics simulations of wild-type CK1 and the *tau* mutant (R178C) that biases kinase activity toward a phosphodegron. We used Markovian state models to integrate the simulations into a single model of the conformational landscape of CK1 and used Gaussian accelerated molecular dynamics to build the first molecular model of CK1 and the unphosphorylated FASP motif. These findings were biochemically validated using in vitro kinase assays and provide a mechanistic view of CK1, establishing how the activation loop acts as a key molecular switch to control substrate selectivity. We show that the wild-type CK1 prefers a "loop down" conformation that binds FASP, whereas the *tau* mutant favors an alternative conformation of the activation loop and significantly accelerates the dynamics of CK1. This reshapes the binding cleft in a way that impairs FASP binding and would ultimately lead to PER destabilization. Finally, we identified a potential binding pocket that could be targeted to influence the conformational state of this molecular switch and lead to predictable changes in circadian period. Our integrated approach offers a detailed model of CK1's conformational landscape and its relevance to normal, mutant, and druggable circadian timekeeping.

SIGNIFICANCE Disruption of circadian rhythms alters the temporal orchestration of vital cellular processes and increases the propensity for sleep disorders, metabolic disease, and cancer. Circadian rhythms are generated by a gene expression program controlled at the cellular level by a molecular clock composed of dedicated clock proteins. Among the essential protein characters is casein kinase 1 (CK1), which acts on multiple clock protein substrates. A delicate balance of CK1 activity on these substrates is crucial for proper circadian timekeeping, highlighting CK1 as a promising drug target to tune clock timing. This work describes the conformational landscape of CK1 that underlies its substrate specificity and provides molecular insight for pharmacologic development that could modulate CK1 function for those suffering from clock-related syndromes.

INTRODUCTION

One of the greatest achievements of life on Earth is the ability of organisms to anticipate the terrestrial cycles of light and darkness. From prokaryotes to mammals, life forms take advantage of day and night to perform cellular tasks in a coherent way by following an internal clock (1–5). Although the biological clock synchronizes to the solar

Submitted January 22, 2025, and accepted for publication September 16, 2025.

*Correspondence: cla.g.ricci@gmail.com or cpartch@ucsc.edu

Editor: Gregory Bowman.

https://doi.org/10.1016/j.bpj.2025.09.022

© 2025 The Author(s). Published by Elsevier Inc. on behalf of Biophysical Society.
This is an open access article under the CC BY license (http://creativecommons.org/licenses/by/4.0/).

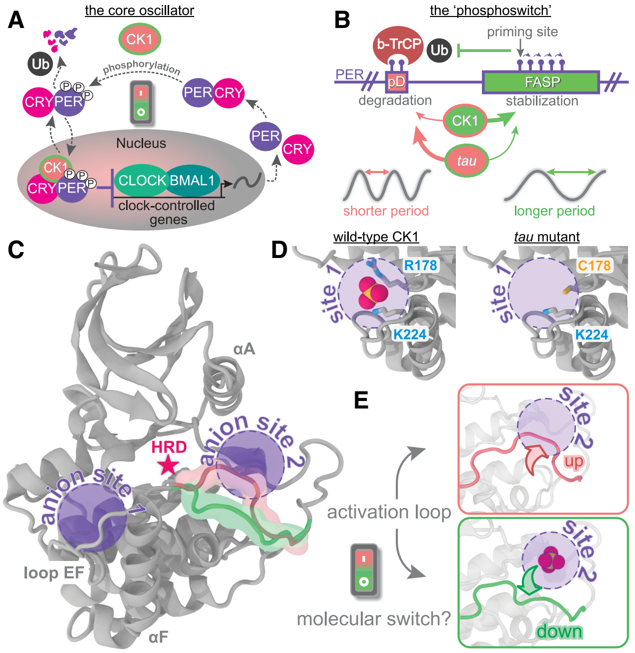

FIGURE 1 The CK1 activation loop acts as a molecular switch to control PER phosphorylation and circadian period. (*A*) Simple schematic view of the transcriptional-translational feedback loop at the core of the human clock. (*B*) The circadian period is finely regulated by a phosphoswitch mechanism controlling PER stability. (*C*) Apo structure of CK1δ (PDB: 1CKJ), highlighting the location of the catalytic HRD motif (*red star*), the two conserved anion binding sites (*purple circles*), and the alternative conformations of the activation loop (*pink and green*). (*D*) A sulfate anion is bound to the first anion site via two positively charged clamps (R178 and K224). The R178C mutation in *tau* impairs the ability of this site to bind anions. (*E*) Alternative conformations of the activation loop in *tau* (PDB: 6PXN), showing that the loop up conformation (*pink*) sterically blocks the second anion site.

day by making use of external cues, its intrinsic "ticking" pace is dictated by an internal core oscillator present in nearly every cell and displaying a period of ∼24 h (6). In mammals, the core oscillator consists of an interlocked transcription/translation feedback loop that generates daily oscillations in gene expression (3,7). This results in a circadian (about a day) expression of proteins involved in behavior, development, metabolism, DNA repair, and more (6). Mutations causing the intrinsic period to be significantly different from ∼24 h can prevent organisms from successfully synchronizing their clocks to the solar day. This results in social jetlag and sleep disorders, which in the long run can interfere with our immune response (8,9) and trigger pathologies such as metabolic syndrome, diabetes, and cancer (6,10–18). In this scenario, understanding the molecular underpinnings of the clock could unlock new pharmacological targets to treat a wide range of diseases.

In humans, the transcription/translation feedback loop is formed by a transcription activator complex, CLOCK: BMAL1, and a repressor complex formed by period proteins (PER1 and PER2), cryptochromes (CRY1 and CRY2), and the protein casein kinase 1 (subtypes δ and ε, hereafter jointly referred to as CK1) (Fig. 1 *A*) (19–23). The CLOCK:BMAL1 dimer activates the transcription of many circadian-controlled genes, including those of their repressors (PERs and CRYs), leading to daily oscillations in repressor expression. Because PER is the stoichiometric limiting factor in the assembly of the CK1:PER:CRY repressor complex (24), its abundance and stability are correlated with the duration of the intrinsic circadian period. The molecular mechanisms regulating the life span of PER proteins thus provide a direct link to circadian period. At the very center of this switch is CK1, which modulates PER stability through posttranslational modifications (25–30) and is thought to confer temperature insensitivity to circadian rhythms (31,32).

CK1 is a ubiquitously expressed serine/threonine kinase with activity against a broad variety of substrates in all cell types (33,34). Orthologs of CK1 have been implicated in timekeeping in a variety of eukaryotic organisms ranging from green algae to humans (35–38), denoting a well-conserved and far-reaching role across species. CK1 displays the typical two-lobed kinase architecture; however, it is not regulated by phosphorylation of its activation loop (Fig. 1 *C*)(33,39,40). Instead, the kinase domain of CK1 is constitutively active (34,41,42), and two highly conserved anion binding pockets play important regulatory roles, including substrate selectivity and recognition (40,43,44). CK1 preferentially acts on negatively charged or primed substrates, such as those with a D/E/pSxxS consensus motif (40,45). Recently, structures of CK1 show that it uses these anion binding sites to bind phosphorylated peptides, either in its own disordered tail (46) or its circadian substrate, PER2 (47). CK1 phosphorylates at least two different regions on PER with antagonist effects on its stability in a phosphoswitch mechanism (Fig. 1 *B*) (48–50). Interestingly, the CK1-dependent phosphorylation sites that form this phosphoswitch in PER do not display this consensus motif and are slow, rate-limiting steps for the circadian clock (25,48). Phosphorylation at phosphodegron (pD) regions recruits the E3 ubiquitin ligase, β-TrCP, and leads to PER degradation, with the ultimate effect of shortening the circadian period (51–55). On the other hand, phosphorylation of five consecutive serines in the FASP region (25) found within the CK1-binding domain of PER leads to its stabilization through feedback inhibition that attenuates kinase activity at the pD (47). Mutation of the first serine in this region causes abnormally short circadian periods and FASP (56,57). Other mutations in CK1/CK1 orthologs (36,38,43,58–63) or in its phosphorylation sites on PER/PER orthologs (49,59) can induce remarkable changes to the intrinsic circadian period, underscoring the central importance of the phosphoswitch for clock timing. Thus far, little is known about the conformational mechanism by which CK1 balances its activity between the pD and FASP regions.

Because its activity and selectivity influences clock periodicity, CK1 is gaining traction as an effective pharmacological target for dysfunctional clocks (27,64). ATP-competitive CK1 inhibitors capable of lengthening the circadian rhythm have been identified (27,65–72), but, unlike many mutations in the enzyme, no small molecules are capable of shortening the period (36,38,58,62). To develop highly specific circadian drugs that diversify our ability to modulate period length, more information is needed on the molecular factors dictating CK1 regulation and substrate selectivity.

The CK1-based circadian mutant *tau* (73) shortens the circadian period by approximately 4 h (61,63). In this mutant, an arginine in the first anion binding pocket is replaced by a cysteine (R178C) (Fig. 1 *D*), impairing the pocket's ability to bind negatively charged groups in primed or acidic substrates (44,73). This site lies near loop EF (L-EF), a flexible region generally implicated in substrate binding by kinases (40,45) and, in the case of CK1, is specifically required for temperature-compensated activity (31). We showed that *tau* simultaneously reduces the activity on the FASP region and increases the activity on the pD site, inverting substrate selectivity on PER relative to the wild-type (WT) enzyme (Fig. 1 *B*) (73). This makes the *tau* mutant an ideal system to study the conformational mechanisms of how CK1 toggles the phosphoswitch. Supported by molecular dynamics (MD) simulations, the crystal structure of *tau* revealed two alternative conformations of the activation loop ("up" and "down," Fig. 1 *E*), hinting at a two-state conformational switch at the core of CK1 substrate selectivity. For circadian substrates, these are the two predominant states of CK1, and they contain the structural features that generally define an active kinase: the DFG motif is positioned inward toward the ATP-binding site with specific dihedral angles between residues that allow the kinase to be catalytically primed, a salt bridge is formed between the helix αC and the N-terminal domain, and the activation loop is positioned correctly at both the C-terminal and N-terminal regions to bind ATP and substrates (74). Interestingly, the less frequent "loop up" conformation of the WT CK1 sterically abolishes the second anion binding pocket, which could be a determining factor for substrate binding and/or product inhibition (73,75).

To investigate how substrate selectivity is encoded in the conformational dynamics of CK1, here we performed a large set of unbiased MD simulations of WT CK1 and the *tau* mutant. These simulations were integrated into a Markov state model (MSM) (76–79) to describe the free energy landscape bridging "loop up" and "loop down" conformations in CK1. MSMs have been emerging as a powerful framework to uncover slow dynamics in complex biomolecular systems (80–82) and to quantify differences between protein ensembles (83–85), many times with significant implications for drug discovery and design (86–90). Here, we additionally used Gaussian accelerated MD (GaMD) (91–94) to boost the sampling of the CK1 conformational landscape and to build the first molecular model of the interaction of CK1 with the priming motif of FASP. Using in vitro kinase assays, we biochemically validated this binding model and identified essential residues necessary for proper CK1 phosphorylation of the stabilizing FASP region. Additionally, we found that FASP binds the "loop down" conformation, whereas the *tau* mutant accelerates CK1 dynamics, favors the "loop up" conformation of the activation loop, and reshapes the binding cleft in a way that hinders FASP binding and ultimately destabilizes PER. Our combined approach provides a comprehensive model of the conformational landscape of CK1 and its implications for circadian timekeeping by establishing the activation loop as a key molecular switch for substrate selectivity. Finally, FTMap identified a potential pocket that could be targeted to bias the "loop up" conformation and ultimately shorten the circadian period.

MATERIALS AND METHODS

Molecular dynamics simulations and related analyses

Initial structures

To simulate *tau* CK1δ, we used PDB: 6PXN (73), starting simulations from both the "loop up" conformation (chain A) and from the "loop down" conformation (chain B). To launch equivalent simulations with the WT CK1, we used PDB: 1CKJ (40), which has the activation loop crystallized both in "up" (chain A) and "down" (chain B) conformations. The structures from PDB: 1CKJ contained two (chain A) or three (chain B) WO_4^{-2} anions, which were computationally replaced by SO_4^{2-} anions at the same positions, to make the simulations of WT CK1δ comparable to the *tau* simulations.

Systems setup

Before simulations, the proteins were minimized using Maestro (Schrödinger), and the final protonation states were estimated using the H++ server (95). All systems were solvated in a preequilibrated cubic TIP3P (96) water box with at least 15 Å between the protein and the box boundaries. The systems net charge was neutralized with Na+ or Cl− counterions. Parameters for proteins and counterions were extracted from the ff14SB force field (97), whereas parameters for the SO_4^{2-} anions were extracted from the Generalized Amber Force Field (GAFF) (98) and adjusted as proposed (99).

Systems equilibration

Minimization and equilibration were performed with AMBER 16 (100), using the following protocol: 1) 2000 steps of energy minimization with a 500 kcal mol^{-1} $Å^{-1}$ position restraint on protein and SO_4^{2-} anions; 2) 1000 steps of energy minimization with a 500 kcal $mol^{-1}Å^{-1}$ position restraint on protein atoms only; 3) 2000 steps of energy minimization without position restraints; 4) 50 ps of NVT simulation, with gradual heating to a final temperature of 300 K, with 10 kcal $mol^{-1}Å^{-1}$ position restraint on protein and SO_4^{2-} anions; 5) 1 ns of NPT simulation to equilibrate the density (or final volume of the simulation box). Temperature was kept at 300 K using the Langevin thermostat and a collision frequency of 2 ps^{-1}. After equilibrating the volume, we ran additional 100 ns of simulations in the NVT regime for each system, using a time step of 2 fs, and all bonds involving hydrogen atoms were restrained with SHAKE (101). The PME method (102) was used to calculate electrostatic interaction using periodic boundary conditions, and a 12-Å cutoff was used to truncate nonbonded short-range interactions.

Gaussian accelerated MD simulations

After equilibration, we ran GaMD simulations with AMBER 17 (103). To boost the exploration of the conformational space, additional acceleration parameters were used, as described previously (91). All systems had a threshold energy E = Vmax and were subjected to a dual boost acceleration of both the dihedral and the total potential energies. To optimize the acceleration parameters, we first ran 2 ns of MD simulations with no boost potential, during which the minimum, maximum, average, and standard deviation (Vmin, Vmax, Vav, σavg) of the total potential and dihedral energies were estimated and used to derive boost potentials as detailed previously (91). These potentials were used to start 50 ns of GaMD simulations, during which the boost statistics and boost potentials were updated until the maximum acceleration was achieved. The maximum acceleration was constrained setting the upper limit of the standard deviation of the total boost potential to be 6 kcal/mol. We ran five replicas of production GaMD simulations for each system with fixed acceleration parameters derived from the previous equilibration stage. Each replica started from the same initial conformation, but the atoms were given different initial velocities, consistent with a Maxwell-Boltzmann distribution at 300 K. Each production simulation ran for 500 ns, totalizing 2.5 μs of sampling for each system, and 15 μs in total.

Conventional MD simulations

To start the MD simulations that would be used to build the MSMs, we extracted conformations from GaMD. For each system (WT and *tau*), 10 different conformations were selected, as described in the supplemental materials and methods, Section A. For each initial structure, we launched three independent MD replicas (with different initial velocities) for 300 ns, totalizing 9 μs for each system (WT or *tau*). After these finished running, we randomly selected 20 more structures from the new trajectories and relaunched two independent MD replicas for 300 ns, adding 12 μs to the total time simulated for each system. Conventional MD simulations were performed with the same methods and parameters as described in the "systems equilibration" subsection.

Markov state models

We used PyEmma version 2.5.7 (104) to process the trajectories and to build, validate, and analyze the MSMs. More details on the construction of the MSM models, including input features, parameters, and validation tests, are described in the supplemental materials and methods, Section B. Jupyter notebooks for the MSM analyses of WT and *tau* CK1 are available at https://github.com/cpartch/CK1, and full trajectories (23 GB data) are available upon request from jmmccammon@ucsd.edu.

Model of CK1 bound to FASP peptide

Due to space limitations, methodological details on the simulations used to create this model can be found in the supplemental materials and methods, Section C.

Mapping of binding pockets on the CK1 surface

This analysis was performed by subjecting representative MD-derived conformations of WT and *tau* CK1 to FTMap (105), a fast computational approach that uses small organic probes to identify consensus sites (or pockets) that are likely to bind drug-like molecules. Due to space limitations, more details on how this analysis was performed are described in the supplemental materials and methods, Section E.

Expression and purification of recombinant proteins

All plasmid purification was carried out in *Escherichia coli* DH5a cells. Proteins were expressed from a pET22-based vector in *Escherichia coli* BL21 (DE3) Rosetta2 cells (Sigma Aldrich) based on the Parallel vector series (106). All FASP peptides were expressed downstream of an N-terminal TEV-cleavable His-NusA tag. Human CK1δ catalytic domains (CK1, residues 1–317) were all expressed in BL21 (DE3) Rosetta2 cells (Sigma Aldrich) with a TEV-cleavable His-GST tag. Mutations were made using standard site-directed mutagenesis protocols and validated by sequencing. All proteins and peptides expressed from parallel vectors have an N-terminal vector artifact (GAMDPEF) remaining after TEV cleavage, and the peptides have a tryptophan and polybasic motif (WRKKK) following the vector artifact. Cells were grown in LB media (for natural abundance growths) or M9 minimal media with the appropriate stable isotopes (i.e., $^{15}N/^{13}C$ for NMR) as done before (25) at 37°C until the $O.D._{600}$ reached ~0.8; expression was induced with 0.5 mM IPTG, and cultures were grown for approximately 16–20 h more at 18°C.

For CK1 protein preps, cells were lysed in 50 mM Tris (pH 7.5), 300 mM NaCl, 1 mM TCEP, and 5% glycerol using a high-pressure extruder (Avestin) or sonicator (Fisher Scientific) on ice. His-GST-CK1 fusion proteins were purified using Glutathione Sepharose 4B resin (GE Healthcare) using standard approaches and eluted from the resin using phosphate buffered saline with 25 mM reduced glutathione. His-TEV protease was added to cleave the His-GST tag from CK1 at 4°C overnight. Cleaved CK1 was further purified away from His-GST and His-TEV using Ni-NTA resin (Qiagen) and subsequent size exclusion chromatography on a HiLoad 16/600 Superdex 75 prep grade column (GE Healthcare) in 50 mM Tris (pH 7.5), 200 mM NaCl, 5 mM BME, 1 mM EDTA, and 0.05% Tween 20. Purified CK1 proteins used for in vitro kinase assays were run on size-exclusion columns or buffer exchanged into storage buffer (50 mM Tris (pH 7.5), 100 mM NaCl, 1 mM TCEP, 1 mM EDTA, and 10% glycerol) using an Amicron Ultra centrifugal filter (Millipore) and frozen as small aliquots in liquid nitrogen for storage at −80°C.

For human PER2 FASP peptide preps, cells were lysed in a buffer containing 50 mM Tris (pH 7.5), 500 mM NaCl, 2 mM TCEP, 5% glycerol, and 25 mM imidazole using a high-pressure extruder (Avestin) or sonicator on ice (Fisher Scientific). His-NusA-FASP fusion proteins were purified using Ni-NTA resin using standard approaches and eluted from the resin using 50 mM Tris (pH 7.5), 500 mM NaCl, 2 mM TCEP, 5% glycerol, and 250 mM imidazole. His-TEV protease was added to cleave the His-NusA tag from the PER2 peptides at 4°C overnight. The cleavage reaction was subsequently concentrated and desalted into low imidazole lysis buffer using a HiPrep 26/10 Desalting column. Peptides were purified away from His-NusA and His-TEV using Ni-NTA resin with 50 mM Tris (pH 7.5), 500 mM NaCl, 2 mM TCEP, 5% glycerol, and 25 mM imidazole. Peptides were purified by size-exclusion chromatography on a HiLoad 16/600 Superdex 75 prep grade column, using NMR buffer (25 mM MES (pH 6.0), 50 mM NaCl, 2 mM TCEP, and 10 mM $MgCl_2$) or 1× kinase buffer (25 mM Tris (pH 7.5), 100 mM NaCl, 10 mM $MgCl_2$, and 2 mM TCEP) for NMR or ADP-Glo kinase assays, respectively.

NMR kinase assays

NMR spectra were collected on a Varian INOVA 600 MHz or a Bruker 800 MHz spectrometer equipped with a ^{1}H, ^{13}C, ^{15}N triple resonance z-axis pulsed-field-gradient cryoprobe. Spectra were processed using NMRPipe (107) and analyzed using CCPNmr Analysis (108). Backbone resonance assignments were determined previously (47). NMR kinase reactions were performed at 25°C with 150 μM ^{15}N-human PER2 FASP, 2.5 mM ATP, and 2 μM CK1. SOFAST HMQC spectra (data acquisition = 5 min) were collected at the indicated timepoints, or HSQC spectra were collected on quenched samples (after addition of EDTA to final concentration of 20 mM) at the indicated timepoints, and the relative peak volumes were calculated and normalized as described previously (25).

ADP-Glo kinase assays

Kinase reactions were performed on the indicated recombinant peptides (FASP WT or alanine mutants) using the ADP-Glo kinase assay kit (Promega) according to manufacturer's instructions. All reactions were performed in 30 μL volumes using 1× kinase buffer (25 mM Tris (pH 7.5), 100 mM NaCl, 10 mM $MgCl_2$, and 2 mM TCEP) supplemented with ATP and substrate peptides. To determine apparent kinetic parameters (K_M), duplicate reactions with 100 μM ATP and 200 nM CK1 kinase were incubated in 1× kinase buffer at room temperature for 1 h with the indicated amount of substrate peptide (and repeated for $n = 2$ independent assays). Five-microliter aliquots were taken and quenched with ADP-Glo reagent after the 1 h incubation, and luminescence measurements were taken at room temperature with a SYNERGY2 microplate reader (BioTek) in 384-well microplates. Data analysis was performed using Excel (Microsoft) or Prism (GraphPad).

Radioactive kinase assays

PER FASP region peptides were synthesized and purified to 95% or higher (SABio). Two independent reaction mixtures of 50 μL containing 200 μM of the FASP or in reaction buffer (25 mM Tris (pH 7.5), 7.5 mM $MgCl_2$, 1 mM DTT, and 0.1 mg/mL BSA) were preincubated for 5 min with or without 20 nM CK1 (for primed FASP) or 200 nM CK1 (for unprimed FASP) and the reaction was started by addition of 750 μM of UltraPure ATP (Promega) containing 1–2 μCi of γ-^{32}p ATP (PerkinElmer). After incubation of the reaction mix at 30°C, an 8-μL aliquot of the reaction mix was transferred to P81 phosphocellulose paper (Reaction Biology Corp) at the indicated timepoints. The P81 paper was washed three times with 75 mM of orthophosphoric acid and once with acetone. The air-dried P81 paper was counted for P_i incorporation using a scintillation counter (PerkinElmer) by Cherenkov counting. Results shown are from four independent assays.

Peptide Name	Species	Peptide Sequence	Figure Shown
PER2 WT (FASP)	mouse	RKKKTEVSAHLSSLTLP GKAESVVSLTSQ	Fig. S15 *B*
PER2 pS659 (primed FASP)	mouse	RKKKTEVSAHLSSLTL PGKAEpSVVSLTSQ	Fig. S15 *C*
PER3 WT	mouse	RKKKPSTDIEGGAAR TLSTAALSVASGISQ	Fig. S15 *E*
PER3 KAE	mouse	RKKKPSTDIEGGAART LSTKAESVASGISQ	Fig. S15 *E*
PER3 WT (priming only)	mouse	RKKKPSTDIEGGAAR TLSTAALSVAAGIAQ	Fig. S15 *F*
PER3 KAE (priming only)	mouse	RKKKPSTDIEGGAAR TLSTKAESVAAGIAQ	Fig. S15 *F*

RESULTS

Mapping the conformational landscape of CK1

The activation loop and loop EF are the slowest loops in CK1

To characterize the dynamics of WT CK1 and the *tau* mutant, we ran multiple all-atom MD simulations totaling 21 μs for each protein system (Fig. S1). We then employed the MSM framework to integrate these trajectories into a single model describing the conformational free energy landscape of CK1 in WT and *tau*. MSMs can derive long timescale dynamics from a large number of relatively short MD simulations by 1) dividing the conformational space into a large number of discrete microstates, 2) using the MD data to count transitions between states after a specified lag time, and finally, 3) estimating a transition matrix that describes the dynamics of the system in the discretized conformational space (76,109). An important step in model building thus consists in selecting relevant features to discretize the conformational space. For our model, we started by selecting pairwise distances involving functionally important regions of CK1 and applied time-lagged independent component analysis (tICA) (110) to reduce these features to a smaller number of collective variables representing the slowest modes of motions (TICs). We found that the slowest and dominant mode of motion in CK1 involves the activation loop (TIC 1) and that interconversion between the "loop up" and "loop down" conformations happens at long scales, elucidating an important regulatory role for this loop (Fig. S2). The second slowest motion (TIC 2) involves L-EF, near the first anion site, which has been previously implicated in substrate binding and temperature-compensation mechanisms (31,40,45). Based on these results, we selected five pairwise distances that were jointly combined by tICA to create the final MSMs. Further methodological details on construction of the models are provided in supplemental materials and methods (see section Markovian state models*;* Figs. S3–S5).

Tau accelerates and inverts the conformational equilibrium involving the activation loop

MSM-based conformational landscapes for WT CK1 and the *tau* mutant reveal that each system displays three preferred states (Fig. 2 *A*). Two of these states are roughly equivalent in WT and *tau* (states I/I' and III/III'), whereas states II and IV' are exclusive of WT and *tau*, respectively. Visual inspection supported by pairwise distances and RMSD (Fig. S6) reveal that the states differ mainly with respect to the conformation of the activation loop (up, down, or intermediate) and the conformational state of L-EF (folded or unfolded) (Fig. 2 *B*). States I/I' and III/III' correspond to "loop up" and "loop down" conformations of the activation loop, respectively, in relatively good agreement with corresponding crystallographic structures. The remaining states display the L-EF in an unfolded state, with the activation loop either adopting an intermediate conformation (state II, in WT) or the "loop down" conformation (state IV,' in *tau*).

Populations derived from the MSM uncover a slow equilibrium between "loop up" (state I) and "loop down" (state III) conformations in the WT kinase, with a clear preference for the "loop down" conformation (Fig. 2 *C*, top). The observation of an additional metastable state (II) in which the activation loop adopts a "halfway" conformation suggests the existence of an intermediate state connecting the two main states (I and III) in the WT enzyme. This

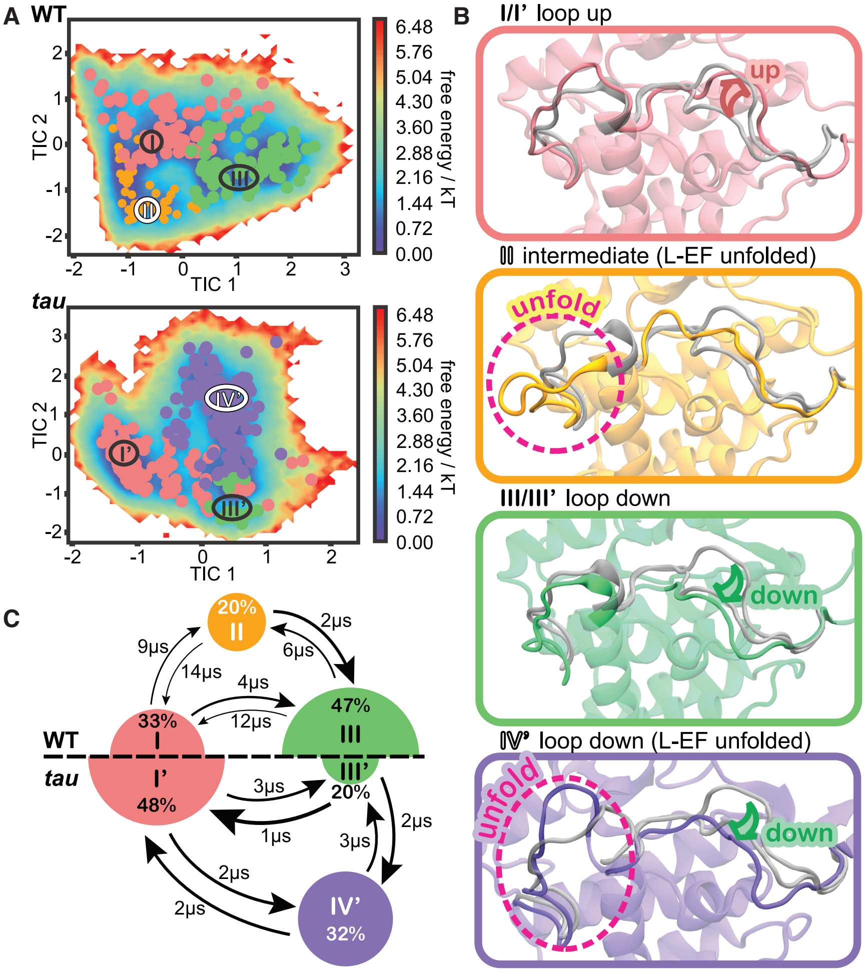

FIGURE 2 The equilibrium between three preferred conformational states is accelerated and inverted by the CK1 *tau* mutant. (*A*) Free energy landscapes of the WT CK1 (*top*) and *tau* mutant (*bottom*) in terms of the slowest tICA components, with microstates clustered into metastable states (*identified by roman numerals*). (*B*) Representative conformations of each metastable state. For comparison, x-ray conformations of the activation loop and L-EF are superimposed (*transparent gray*; loop down PDB: 1CKJ, loop up PDB: 6PXN). (*C*) Equilibrium populations of metastable states and MFPTs between states for the WT (*top*) and *tau* (*bottom*) protein systems. Radius of the circles is proportional to the equilibrium population percentage, and thickness of the arrows is proportional to transition rates between states. The numbers next to the arrows indicate MFPTs.

intermediate state also suggests that flipping of the activation loop requires disorganization of L-EF, which displays significantly more conformational freedom in state II (Fig. S6).

Interestingly, the *tau* mutation inverts the conformational equilibrium characteristic of WT, stabilizing the activation loop in the "up" conformation (state I') and destabilizing the "down" conformation (Fig. 2 *C*, bottom), which is almost always accompanied by unfolding of L-EF (state IV'). In addition, flipping of the activation loop between up and down conformations does not involve a long-lived intermediate or "midway" conformation. Such inversion of the conformational landscape strongly supports the activation loop as a key molecular switch controlling the circadian period since the *tau* mutant is known for inverting CK1 selectivity for its circadian substrates. Our model also recapitulates previous findings that the *tau* mutation destabilizes the first anion site, facilitating the unfolding of L-EF, but "only" when the activation loop is down (state IV') (73). When the activation loop is up, L-EF remains folded and displays WT-like dynamics (state I'), supporting that the activation loop exerts allosteric control over the dynamics of L-EF.

Apart from equilibrium populations, MSMs also provide transition rates between states, informing on the kinetics of the system. By comparing mean first passage times (MFPTs) between states (Fig. 2 *C*, arrows), we find that the *tau* mutation significantly accelerates the conformational transitions between states. Interconversion between loop up and down conformations in the *tau* mutant (I' ⇔ IV' ≈ 2 μs) is at least two times faster than in the WT enzyme (I ⇔ III ≈ 4–12 μs), indicating that the *tau* mutation significantly reduces the energy barriers associated with flipping of the activation loop. This agrees with the lack of intermediate "midway" conformations in the *tau* mutant. *Tau* also accelerates unfolding of L-EF, which in state IV' adopts a wide range of conformations ranging from collapsed to fully extended (see Fig. S6 *B*).

The role of Gly^{175} in the conformational dynamics of the activation loop

The activation loop in CK1 is preceded by a conserved glycine at position 175, distinct from the glycine found in the classic DFG motif (Gly 151) that flips between catalytically active and inactive conformations. In other serine/threonine kinases,

a backbone flip of a glycine at this conserved position may be correlated with conformational changes of the activation loop, and therefore, it could link these loop conformations to the first anion binding pocket *tau* mutation site (R178C) (111). In CK1, x-ray structures suggested that Gly^{175} backbone could work as a "hinge" controlling the conformation of the activation loop (Fig. 3 *A*) (40,73). To investigate this hypothesis, we built MSMs based solely on the backbone angles of Gly^{175} (Figs. S7 and S8).

The resulting Gly^{175}-based MSMs revealed no significant correlation between the configuration of Gly^{175} and the conformation of the activation loop in WT CK1 (Fig. S9). Thus, although the configuration of Gly^{175} might determine the most energetically stable conformation of the activation loop in low entropy scenarios (crystalline state), its importance appears to be overcome by other factors when the protein is in solution. Surprisingly, the *tau* mutant displays a moderate correlation between φ^{Gly175} and the activation loop ($\varphi^{Gly175} < 0°$ favors "loop up" and $\varphi^{Gly175} > 0°$ favors "loop down"), indicating that Gly^{175} backbone is more determinant of the conformation of the activation loop in the *tau* mutant than in the WT enzyme.

In part, this can be explained by the energetic barriers separating states in the conformational landscape of CK1. In the WT enzyme, a configurational torsion of Gly^{175} alone is not enough to overcome the high energy barriers associated with the flip of the activation loop. In the *tau* mutant, these barriers are decreased by disruption of the first anion binding site and unfolding of L-EF, allowing the conformational dynamics of the activation loop to be influenced more heavily by the backbone configuration of Gly^{175}. This is in excellent agreement with MFPTs provided by the MSMs that show that torsional transitions of Gly^{175} happen at timescales faster than 4 μs (Fig. S8 *A*), the same timescale range at which the activation loop flips in the *tau* mutant (see Fig. 2 *C*). In WT CK1, loop transitions happen at much longer timescales (4–12 μs) (see Fig. 2 *C*), indicating that the conformational landscape in the WT enzyme is governed by slow cooperative motions, likely related to the conformational state of L-EF (Fig. 3 *B*).

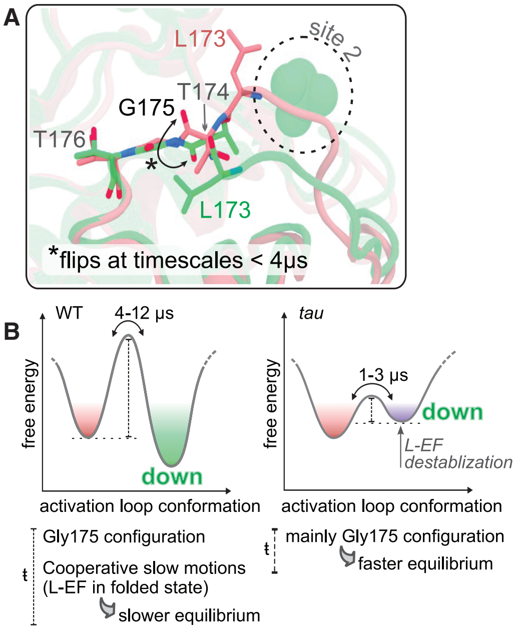

FIGURE 3 The role of Gly^{175} for the conformational dynamics of the activation loop. (*A*) View of x-ray structures highlighting the different configurations adopted by Gly^{175} in "loop up" and "loop down" conformations of the CK1 activation loop (PDB: 6PXN and 1CKJ, respectively). (*B*) Schematic representation of the conformational landscape involving the activation loop, based on MSM-derived kinetics and stability of the two most populated states in each system (WT CK1, *top*; and *tau*, *bottom*).

A molecular model of FASP interactions involved in priming

To gain a better understanding on how the conformational landscape of CK1 is linked to its activity on circadian substrates, we decided to model the interaction between CK1 and the FASP region of PER2 (Fig. 4 *A*). We based our initial model on a recently published crystallographic structure of CK1 bound to TAp63α (Fig. S10, PDB: 6RU7, chains A and C) (75). As with FASP, TAp63α also undergoes sequential phosphorylation by CK1, with the difference that the priming is achieved by another kinase, CDK2 (112). We refined our model with a combined set of 2 μs of accumulated GaMD simulations followed by additional 2 μs of accumulated cMD simulations (Fig. S11, details in supporting material).

FASP binds to the loop down conformation

Our final model consists of a FASP-bound conformation in which the priming serine (S662 in human PER2) is well positioned to accept the γ phosphate from ATP, with the activation loop in the "down" conformation (Figs. 4 *B* and S11 *B*). V663 (at position +1) appears to play a key role in anchoring the backbone of FASP into the active site (see Fig. 4 *B*), displaying low atomic fluctuations (Fig. 4 *C*) and engaging in persistent hydrophobic interactions with Tyr^{225} in helix αF and with Leu^{173} in the activation loop (Fig. 4 *D*).

Electrostatic interactions upstream of the priming site of FASP contribute to substrate binding

Although residues downstream of the priming site display high mobility freedom and a lack of persistent interactions with CK1, the upstream region of FASP is less dynamic and likely to contribute to substrate binding (Fig. 4 *C*). We noticed that the first anion binding site is partially occupied by E661 (at position −1), which displays persistent electrostatic interactions with Arg^{178} but not as much with Lys^{224} (Fig. 4 *E*). In the *tau* mutant, Arg^{178} is replaced with a cysteine, likely disrupting this interaction. We also found that K659 (at position −3) invariably forms a salt

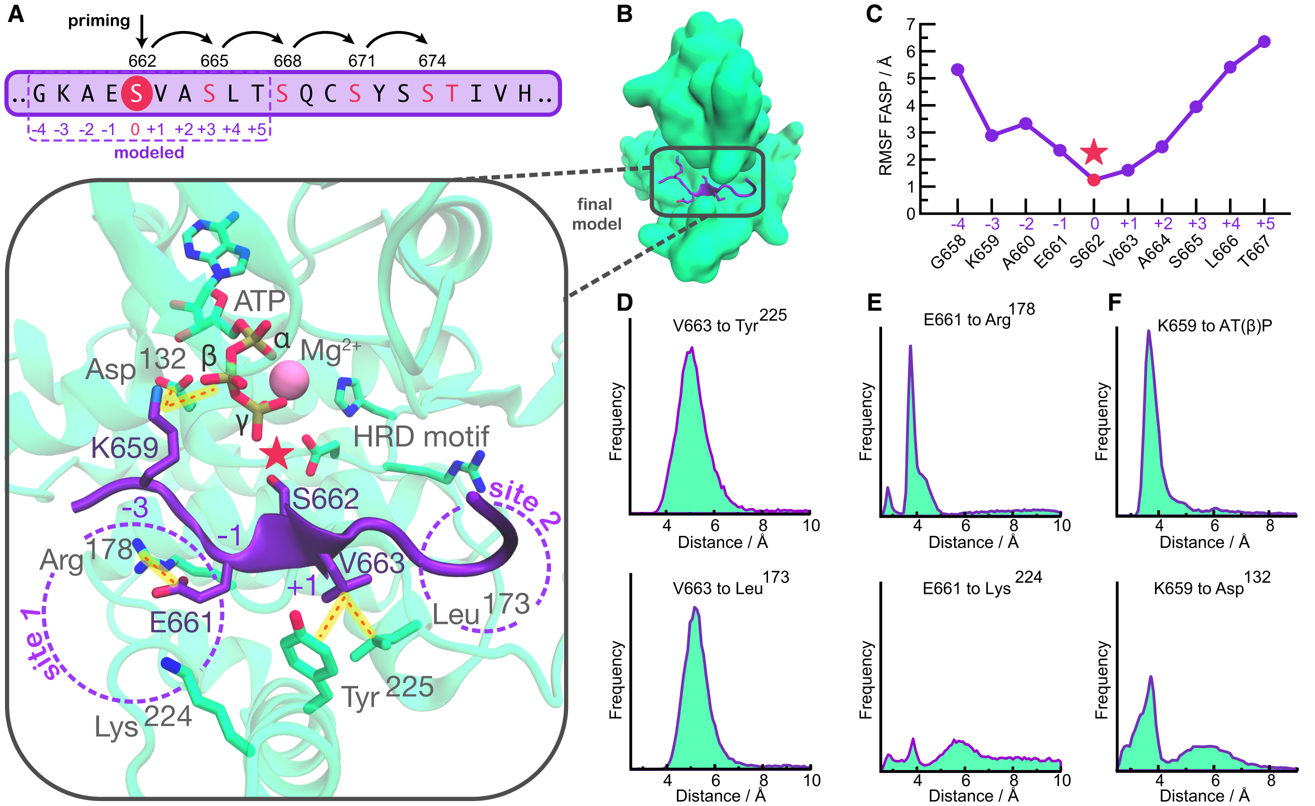

FIGURE 4 Molecular model of the interaction between CK1 and unphosphorylated FASP. (*A*) The FASP region of human PER2. Residue numbers with arched arrows represent serines that are sequentially phosphorylated by CK1. Positions listed below (−4 to +5) are relative to priming serine, S662. (*B*) Representative structure of the final model with the unphosphorylated FASP peptide (*purple*) and CK1 (*green*). For clarity, we use one-letter amino acid code for residues of the substrate (FASP) and three-letter code for residues of the kinase CK1. Pink star represents the priming event of S662. (*C*) Atomic fluctuations of the bound FASP based on accumulated molecular dynamics trajectories. (*D–F*) Distance-based interaction histograms involving V663 at position +1 (*D*), E661 at position −1 (*E*), and K659 at position −3 (*F*) with CK1 residues or ATP.

bridge with the β phosphate of ATP, oftentimes assisted by an additional salt bridge with an Asp^{132} (just downstream of the catalytic HRD motif) (Fig. 4 *F*).

Biochemical validation of the PER FASP priming model

To support our model of unprimed FASP bound to CK1, we performed a series of biochemical experiments to assess the relative contribution of residues near the FASP priming site as molecular determinants of CK1 priming activity. As we have done previously (25,47,73), we used an NMR-based kinase assay to measure priming activity within human PER2 FASP peptides with site-specific resolution. In agreement with our model, introducing alanine mutations to the −3, −1, or +1 positions of FASP led to a decrease in priming activity (Figs. 5 *A* and S14).

To get a further sense of how these mutations might contribute to the binding of unprimed FASP, we introduced an alanine mutation at the +4 Ser (S665) to limit kinase activity to just the priming site (Fig. S15 *A*). We then performed substrate titrations of FASP peptides with alanine mutations at the −3, −1, and +1 positions in this "priming only" (S665A) background by ADP-Glo assay. Similar to the NMR assay, the −3 and −1 alanine mutations led to an approximate 10-fold increase in the apparent Michaelis-Menten constant ($K_M$app) relative to the WT substrate ($K_M$app = 210.3 ± 0.3 μM); the $K_M$app for +1 alanine mutant could not determined due to its low activity. Together, these values suggest significantly reduced affinity of the unprimed mutant FASP substrates for the kinase (Fig. 5 *B*). Mutation of the +1 valine in FASP had the largest decrease in kinase activity, in agreement with lower overall atomic fluctuations displayed by this residue in our MD model (Fig. 4 *C*). Moreover, we found that priming activity can be rescued at the downstream serine in a "priming deficient" S662A mutant by simply substituting a valine for the +1 leucine at the second phosphorylation site (i.e., FASP S662A/L666V, Fig. 5 *C*). Taken together, these results indicate that priming of FASP is highly sensitive to the presence of a valine at the +1 position. Interestingly, the hydrophobic pocket occupied by this valine is created between Tyr^{225} and Leu^{173} of CK1 only when the activation loop is down. The +1 hydrophobic pocket is also small and likely contributes to substrate selectivity based on steric occlusion of bulkier residues at the +1 position, considering

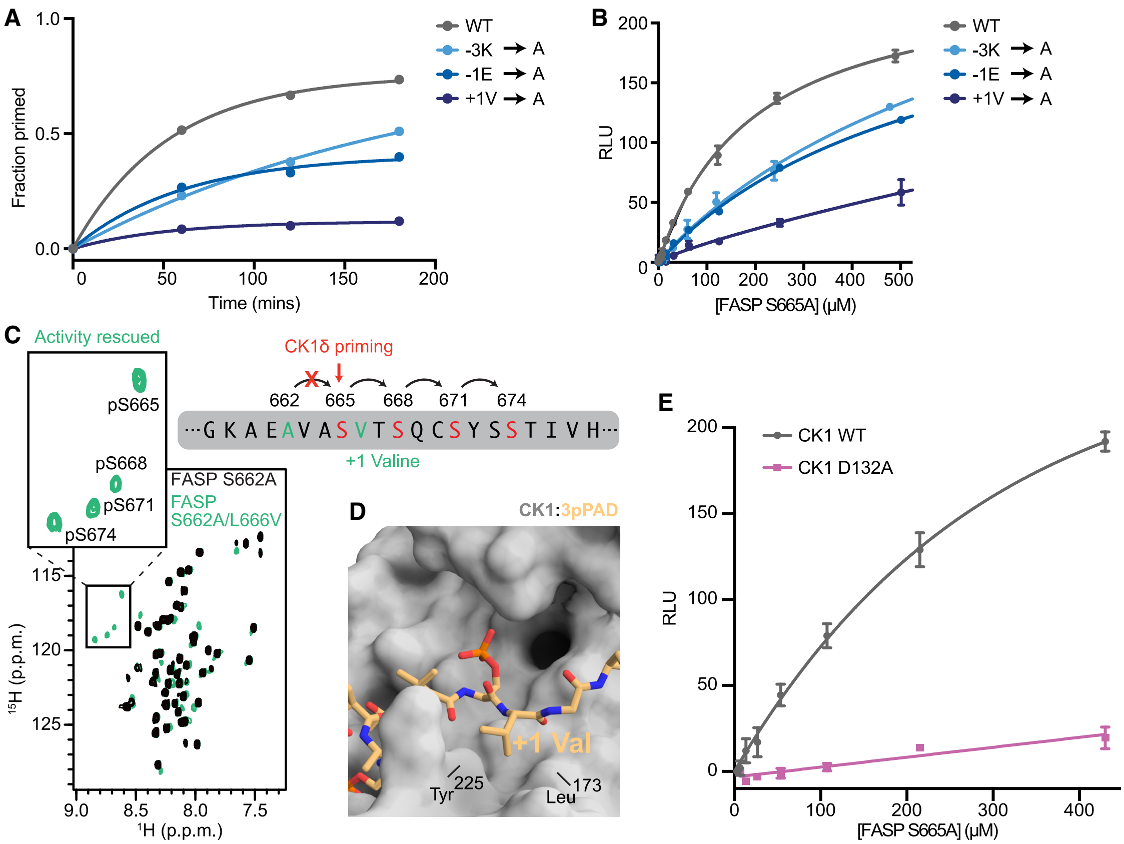

FIGURE 5 Biochemical validation of the model for nonconsensus FASP priming. (*A*) NMR-based time course kinase assay quantifying the increase in peak volume corresponding to the phosphopeak of the priming serine in human PER2 FASP (S662), taken from a series of ^{15}N-^{1}H HSQC spectra (Fig. S14). (*B*) Representative ADP-Glo titration assay comparing WT FASP and alanine mutants at positions −3, −1, and +1 in the "priming only" background (S665A mutation to halt sequential kinase activity; see Fig. S15 *A*) from replicate titration experiments $n = 2$, mean ± standard deviation. (*C*) ^{15}N-^{1}H HSQC spectra comparing a "priming disrupted" FASP peptide (S662A, *black*) and a "priming rescued" FASP peptide (S662A/L666V, *teal*) in the presence of CK1. Zoom shows the region of the spectra where phosphoserines appear. (*D*) Zoom of co-crystal structure of CK1 and a triply phosphorylated Tap63α PAD peptide, 3pPAD, highlighting the active site and +1 hydrophobic pocket region (PDB: 6RU8). The +1 valine residue is inserted between the small hydrophobic pocket created between Tyr^{225} and Leu^{173} of CK1 when the activation loop is in the downward conformation. (*E*) ADP-Glo titration of the "priming only" (S665A) FASP peptide comparing CK1 WT (*black*) and D132A (*purple*) from replicate titration experiments $n = 2$, mean ± standard deviation.

that co-crystal structures of CK1 bound to phosphorylated Tap63α PAD and FASP peptides are composed entirely of backbone-backbone interactions between peptide and kinase in this region (Fig. 5 *D*).

We further tested our FASP binding model by introducing an alanine mutation to CK1 at Asp^{132} (D132A) because this residue frequently interacted with the −3 lysine of FASP over the course of the MD trajectories. Titration of the "priming only" FASP peptide (S665A) comparing CK1 WT and D132A showed a dramatic loss of kinase activity (Fig. 5 *E*). Since Asp^{132} is located directly under the nucleotide binding site and potentially in position to contact ATP, we also sought to test whether the D132A mutation would disrupt kinase activity on a primed FASP peptide, where CK1 activity is driven by the consensus recognition mechanism involving the first anion binding site. Although the D132A mutation showed a dramatic loss in activity on the unprimed FASP peptide, it had a more modest effect on the primed FASP peptide (Fig. S15 *B* and *C*), suggesting that this mutation primarily reduces activity on FASP via recognition of the unprimed substrate.

We showed that CK1 phosphorylates the nearly identical PER1 FASP sequence comparable to PER2, both in vitro and in cells (47). However, one PER homolog within the circadian system, PER3, does not appear to be a substrate of the kinase and lacks the critical residues that precede the priming serine (113). Here, we further tested our model by introducing a lysine at the −3 position and a glutamate at the −1 position of PER3 FASP-like peptides (A610K/L612E) to mimic the PER2 FASP priming region and rescue priming activity. These mutant peptides were used in a ^{32}P-ATP time course kinase assay and showed an increase in CK1 activity for both the WT (Fig. S15 *E*) and the "priming only" (Fig. S15 *F*) substrates in the presence of a −3K and −1E, further demonstrating that these residues play an important role in CK1 recognition and activity on FASP substrates. An alignment comparing these CK1 motifs with other known CK1-targeted poly-SXXS motifs shows

that the −3K, −1E, and +1V seem to be conserved just among the PER substrates (Fig. S16).

Mapping of binding pockets on the CK1 surface

Our MSMs support that inversion of substrate selectivity in the *tau* mutant is achieved by inversion of the conformational equilibrium in the activation loop, with destabilization of the "loop down" conformation in favor of the alternative "loop up." To understand how the activation loop reshapes the molecular surface of CK1 and to look for potential binding sites controlling substrate selectivity, we screened the CK1 surface using FTMap (105). For more details, see supplemental materials and methods, Section E.

The activation loop significantly reshapes the substrate binding cleft in CK1

For both the WT and *tau* mutant, the top-ranked pockets identified by FTMap correspond to the ATP-binding site and the Mg^{2+} pocket near the catalytic site (Tables S7–S10). Interestingly, we observed a fragmentation of the active site and substrate binding cleft in the *tau* mutant, which break down into three separate subpockets not as well ranked as the large contiguous pocket detected in the WT enzyme (Figs. S16 and S17). Comparison of these pockets when CK1 is in "up" or "down" conformation reveal how dramatically the activation loop conformation reshapes the substrate binding cleft (Fig. 6). The "loop down" conformation promotes a straight binding cleft running contiguously from the first to the second anion binding site, above the activation loop (Fig. 6 *A*). The "loop up" conformation, however, appears to promote a bent binding cleft, with part of the substrate channel in the space between helices αD and αF, below the activation loop (Fig. 6 *B*). Considering that the WT CK1 has a higher preference for FASP, we hypothesized that the straight binding cleft produced by the more common "loop down" conformation is well suited to bind the FASP substrate, in agreement with our recent co-crystal structures of WT CK1 bound to phosphorylated FASP peptides (pFASP) (Fig. 6 *C*) (47). The bent binding cleft produced by "loop up" conformations could more favorably interact with the pD substrate, which could explain how *tau* not only reduces activity on FASP but also increases the activity on pD substrates. Interestingly, we found that a *Drosophila* PER (dPER) peptide phosphorylated at S589 (perShort peptide), the site of the per^S mutation that destabilizes dPER and shortens circadian period (114), binds to CK1 when the substrate binding cleft is bent (47). The pS589 of the perShort peptide coordinates the first anion binding site identically to pFASP but follows a channel exposed by the "loop up" conformation of the activation loop (Fig. 6 *D*), in excellent agreement with the FTMap analysis. We did not submit the intermediate state of the activation loop (state II) in this analysis because, due to the high level of disorder in the

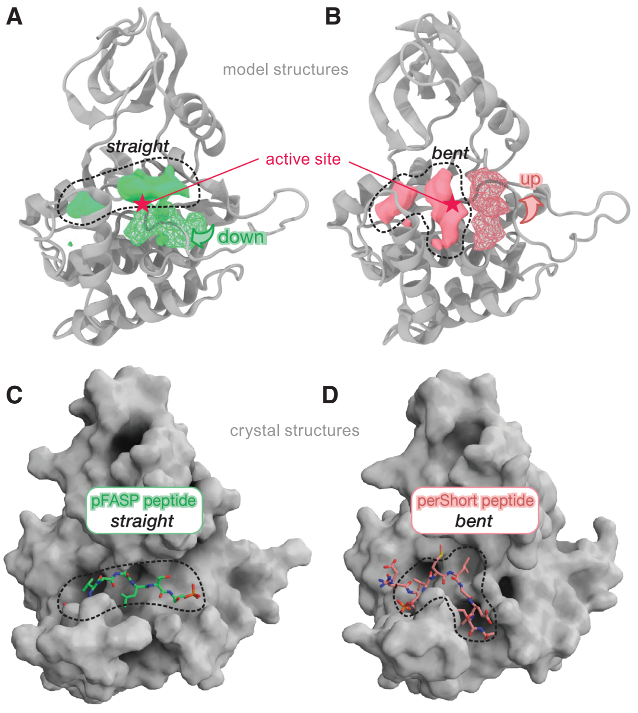

FIGURE 6 The conformation of the activation loop shapes the substrate binding cleft. (*A*) The "loop down" conformation of the activation loop creates a straight binding cleft, whereas (*B*) the "loop up" conformation creates a bent binding cleft, opening a subpocket right under the active site. The solid blobs represent density maps computed based on organic probes from FTMap, whereas the meshed blobs represent occupancy maps computed for residues Lys^{171}, Asn^{172}, and Leu^{173} along the simulations. (*C* and *D*) Co-crystal structure of human PER2 pFASP bound to CK1 (PDB: 8D7M) in the straight substrate binding cleft (*C*), whereas the dPER perShort peptide binds to CK1 (PDB: 8D7P) following a bent substrate binding cleft (*D*).

L-EF region observed in this state, we do not expect it to be able to hold substrates in the binding cleft.

A binding pocket to shorten the circadian rhythm

FTMap also identified potential binding sites on the surface of CK1, as described in supplemental materials and methods, Section E. Of particular interest is a pocket formed between helix αC and the activation loop (Fig. 7 *A*). As highlighted in Fig. 7 *A*, this "activation" pocket includes three residues belonging to the activation loop (Lys^{171}, Asn^{172}, and Leu^{173}) and it is only fully assembled when the activation loop is up. Indeed, this pocket is ranked higher in the *tau* mutant (Table S10) than in the WT enzyme (Table S9), in agreement with the fact that *tau* stabilizes the upward conformation of the activation loop. Other key residues forming this potential binding pocket are His^{46} and Pro^{47} located in the loop connecting sheet β3 to helix αC (Fig. 7 *B*). Interestingly, mutations at these positions (H46R and P47S) of the *Drosophila* CK1 ortholog, *Doubletime,* shorten the circadian period by ∼4 h (62). Thus, occupation of this binding pocket by small molecules mimicking the sidechains of serine and arginine could

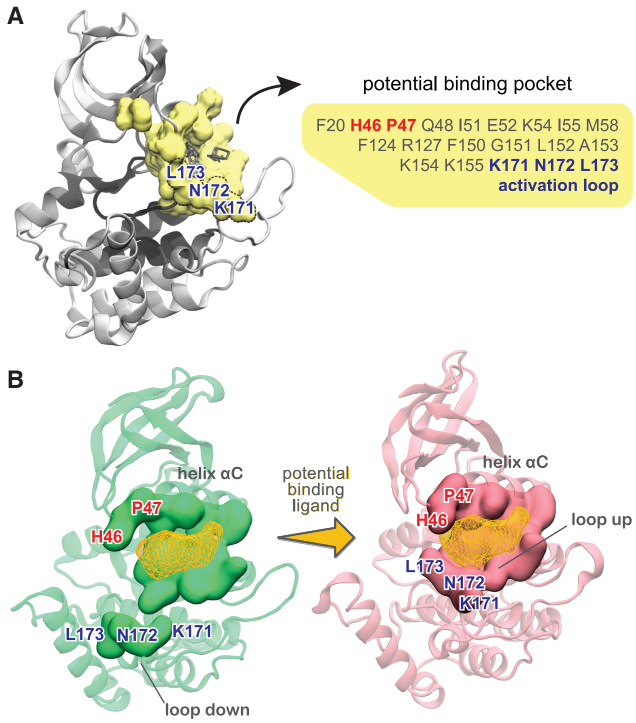

FIGURE 7 A potential binding pocket to shorten the circadian rhythm. (*A*) Residues belonging to the *tau* activation pocket are highlighted in yellow, whereas the small organic molecules used as probes by FTMap are represented as gray sticks. (*B*) Complete assembly of this pocket occurs when the activation loop is "up." Ligand binding to this pocket is likely to stabilize the "loop up" conformation.

stabilize the "loop up" conformation and bend the substrate binding cleft. This could increase CK1 activity against the pD and ultimately shorten the circadian period. A comparison of this potential CK1 pocket to known targeted pockets on several other kinase domains is provided in Fig. S19.

DISCUSSION

Despite its central role in governing circadian rhythms, the molecular mechanism underlying CK1 selectivity for its target regions of the circadian protein PER remains poorly understood, hindering rational attempts to develop CK1-specific circadian drugs. CK1's active site, activation loop, and three anion binding sites are used to bind phosphorylated peptides (46,47) and are extremely well conserved across eukaryotes (73). However, because CK1 is generally thought to be constitutively active toward primed or acidic substrates (34,40–42,45), the role of its activation loop has been largely unexplored. Efforts to characterize serine/threonine kinase specificity in relation to substrate sequence variation have typically identified just the high-activity, primed consensus motif for CK1 (115). Additionally, many CK1 substrates with multiserine clusters motifs are initiated by priming phosphorylation from another kinase. However, CK1 activity on circadian substrates is distinctive because it acts on slower, nonconsensus phosphorylated motifs (25,47,116). To determine the molecular basis of CK1 activity specifically at these nonconsensus motifs, we used an extensive set of MD simulations integrated into MSMs to map the free energy landscape governing the slow conformational transitions of CK1's activation loop. Our model revealed the existence of a slow equilibrium between the more stable "loop down" and the less stable "loop up" conformations in the WT enzyme and showed that the period-shortening *tau* mutant biases and accelerates the transitions toward the "up" conformation.

We also found that the dynamics of the activation loop are strongly connected to the conformational state of L-EF and that flipping of the activation loop requires transient unfolding of L-EF. This explains the long timescales associated with "up-down" transitions, which would happen faster if the conformation of the activation loop was controlled mainly by the configuration of Gly^{175}, as previously hypothesized (73). Elucidation of the role of Gly^{175} for the conformational dynamics of the activation loop reinforces the importance of MD in re-visiting and adding complexity to structural hypotheses that are based on static observations. These findings also explain how the R178C *tau* mutation, which facilitates the unfolding of L-EF by disrupting the first anion binding site, significantly accelerates the dynamics of the activation loop. Interestingly, given the importance of L-EF for temperature-compensation against circadian substrates (31,40,45) and its allosteric connection with the activation loop (47), it is likely that the dynamic equilibrium involving the activation loop is also part of CK1 temperature-compensation mechanisms.

This work characterizes for the first time to our knowledge how CK1 interacts with the unprimed PER FASP region. Our model of the FASP interaction with CK1 suggests key interactions important for the nonconsensus priming step that are supported by in vitro biochemistry. In agreement with the relatively low atomic fluctuations of the +1 residue (relative to the priming site) from our model, our data clearly demonstrate the importance of the +1 valine (+1V) for nonconsensus priming of FASP, which fits into a small hydrophobic pocket created between Leu^{173} and Tyr^{225} on CK1. Our model also reveals that FASP makes use of the first anion binding site by partially inserting a negatively charged glutamate at position −1 (−1E) of the substrate. Additionally, we showed that stabilization of FASP in the binding cleft is complemented by electrostatic interactions between the lysine at position −3 (−3K) with Asp^{132} of CK1 as well as the β-phosphate group of ATP. Finally, we demonstrated that addition of a −3K and −1E to PER3 was sufficient to allow phosphorylation of the priming serine by CK1. Together these findings establish −3K, −1E, and +1V as bona fide residues necessary for proper CK1 recognition of FASP as a substrate.

Interestingly, although the −3K-ATP interaction can lock the FASP substrate in place for the priming event, we

speculate that this interaction could also facilitate unbinding (or translocation) of the primed product by attaching it to the leaving ADP subproduct. In addition, substrate stabilization achieved by partial occupation of the first anion site by −1E could be just enough to promote priming without excessive stabilization of the primed product. In consensus substrates, this anion site is fully occupied by a negatively/phosphorylated residue at position −3, as recently shown in the structures of CK1 bound to primed Tap63a or PER FASP peptides (47,75). It can even be double occupied (by phosphorylated serines at positions −3 and −6) in the case of multiply phosphorylated Tap63α (75), or as seen in one crystal structures of CK1 in anionic solvent conditions (31). This suggests that the partial occupation of the first anion site by unprimed FASP is likely to be replaced with progressively stronger occupation of this site by a phosphorylated serine at position −3 to allow subsequent phosphorylation events. In a processive or semiprocessive mechanism, these escalating electrostatic interactions at the upstream region of FASP would provide a direction for the sequential phosphorylation events, whereas the lack of specific interactions in the downstream region would make it easier for the next phosphorylation site to translocate into the active site.

Using FTMap to map the surface of CK1 in different conformations, we demonstrated how the conformation of the activation loop reshapes the substrate binding cleft. Applying FTMap to the conformations extracted from our MSM also allowed us to identify a potentially attractive binding site between helix αC and the activation loop. Interestingly, this site partially overlaps with the site that accommodates a phosphate group in kinases that are activated by phosphorylation of the activation loop (73), suggesting a possible regulatory role. To add to that, two period-shortening mutations are found in this site: P47S and H46R. Differently from *tau*, both P47S and H46R mutants achieve their period-shortening effects by increasing activity on the pD region while retaining normal FASP priming activity relative to WT (73). This is consistent with what would be expected from increasing the population of “loop up” and “bent” substrate binding cleft conformations (which favor pD phosphorylation) without disruption of the first anion binding pocket (important for priming and subsequent phosphorylation of FASP). Targeting this pocket could help to bias the conformation of CK1 and have predictable effects on circadian period. Because this pocket is only fully assembled when the activation loop is “up,” we propose that it might be a viable site to pharmacologically stabilize the “up” conformation and shorten the period of circadian rhythms.

CONCLUSION

Because PER stability is directly linked to clock periodicity, maintaining an ∼24-h circadian rhythm requires a delicate balance between its two antagonistic CK1 phosphorylation targets: the FASP and degron regions. CK1 balances its activity against these two substrates by using its activation loop as a molecular switch, forming specific binding cleft conformations when the loop is either up or down. This switch from “loop up” and “loop down” is controlled by a slow equilibrium dependent on the activation loop’s allosteric connection to loop EF and the anion binding sites. The circadian mutant *tau* accelerates the dynamics between the activation loop conformational states and stabilizes the “loop up” position, biasing kinase activity toward the degron that results in a shorten circadian period. *Tau* exemplifies the necessity for steep energetic barriers between the “loop up” and “loop down” conformations to control proper circadian timekeeping. Additionally, CK1 recognizes the FASP substrate lacking a consensus motif by using conserved residues surrounding the priming serine. This forms the molecular basis for the critical slow, rate-limiting priming step within the PER phosphoswitch mechanism. Finally, this work identifies a potential CK1 binding site that could be targeted to stabilize the activation “loop up” conformation to modulate phosphorylation activity toward the degron, expanding our therapeutic development strategies to fine-tune circadian period.

DATA AND CODE AVAILABILITY

Jupyter notebooks and data for Markov state models are provided at GitHub/cpartch/CK1. Instructions for running simulations can be found in the supporting material.

ACKNOWLEDGMENTS

Funding for this work was provided by the US National Institutes of Health grant R35 GM141849 (C.L.P.). D.M.V. was supported by Singapore Ministry of Health grant MOH-000600. J.A.M. was supported by TSCC computer resources from UC San Diego. C.L.P. was supported by the Howard Hughes Medical Institute.

AUTHOR CONTRIBUTIONS

C.G.R. and J.M.P. designed research, performed experiments, analyzed data, and wrote the manuscript. M.R.T. prepared materials, performed experiments, and edited the manuscript. A.M.F. and R.N. prepared materials, performed experiments, and analyzed data. E.P. performed experiments and analyzed data. R.A., D.M.V., J.A.M., and C.L.P. guided research, funded research, and edited the manuscript.

DECLARATION OF INTERESTS

The authors do not declare any conflicts of interest.

SUPPORTING MATERIAL

Supporting Material can be found online at https://doi.org/10.1016/j.bpj.2025.09.022.

REFERENCES

1. Aschoff, J. 1965. Circadian Rhythms in Man. *Science.* 148:1427–1432.
2. Rosbash, M. 1995. Molecular Control of Circadian-Rhythms. *Curr. Opin. Genet. Dev.* 5:662–668.
3. Partch, C. L., C. B. Green, and J. S. Takahashi. 2014. Molecular architecture of the mammalian circadian clock. *Trends Cell Biol.* 24:90–99.
4. Cohen, S. E., and S. S. Golden. 2015. Circadian Rhythms in Cyanobacteria. *Microbiol. Mol. Biol. Rev.* 79:373–385.
5. Eelderink-Chen, Z., J. Bosman, …, M. Merrow. 2021. A circadian clock in a nonphotosynthetic prokaryote. *Sci. Adv.* 7:eabe2086.
6. Bass, J., and M. A. Lazar. 2016. Circadian time signatures of fitness and disease. *Science.* 354:994–999.
7. Takahashi, J. S. 2017. Transcriptional architecture of the mammalian circadian clock. *Nat. Rev. Genet.* 18:164–179.
8. Mazzoccoli, G., M. Vinciguerra, …, A. Relógio. 2020. The Circadian Clock, the Immune System, and Viral Infections: The Intricate Relationship Between Biological Time and Host-Virus Interaction. *Pathogens.* 9:83.
9. Ray, S., and A. B. Reddy. 2020. COVID-19 management in light of the circadian clock. *Nat. Rev. Mol. Cell Biol.* 21:494–495.
10. Roenneberg, T., and M. Merrow. 2016. The Circadian Clock and Human Health. *Curr. Biol.* 26:R432–R443.
11. Hatori, M., C. Vollmers, …, S. Panda. 2012. Time-Restricted Feeding without Reducing Caloric Intake Prevents Metabolic Diseases in Mice Fed a High-Fat Diet. *Cell Metab.* 15:848–860.
12. Roenneberg, T., K. V. Allebrandt, …, C. Vetter. 2012. Social jetlag and obesity. *Curr. Biol.* 22:939–943.
13. McHill, A. W., E. L. Melanson, …, K. P. Wright, Jr. 2014. Impact of circadian misalignment on energy metabolism during simulated nightshift work. *Proc. Natl. Acad. Sci. USA.* 111:17302–17307.
14. Sulli, G., M. T. Y. Lam, and S. Panda. 2019. Interplay between Circadian Clock and Cancer: New Frontiers for Cancer Treatment. *Trends Cancer.* 5:475–494.
15. Sulli, G., E. N. C. Manoogian, …, S. Panda. 2018. Training the Circadian Clock, Clocking the Drugs, and Drugging the Clock to Prevent, Manage, and Treat Chronic Diseases. *Trends Pharmacol. Sci.* 39:812–827.
16. Stenvers, D. J., F. A. J. L. Scheer, …, A. Kalsbeek. 2019. Circadian clocks and insulin resistance. *Nat. Rev. Endocrinol.* 15:75–89.
17. Sinturel, F., V. Petrenko, and C. Dibner. 2020. Circadian Clocks Make Metabolism Run. *J. Mol. Biol.* 432:3680–3699.
18. Dong, Z., G. Zhang, …, J. N. Rich. 2019. Targeting Glioblastoma Stem Cells through Disruption of the Circadian Clock. *Cancer Discov.* 9:1556–1573.
19. Partch, C. L. 2020. Orchestration of Circadian Timing by Macromolecular Protein Assemblies. *J. Mol. Biol.* 432:3426–3448.
20. Cox, K. H., and J. S. Takahashi. 2019. Circadian clock genes and the transcriptional architecture of the clock mechanism. *J. Mol. Endocrinol.* 63:R93–R102.
21. Aryal, R. P., P. B. Kwak, …, C. J. Weitz. 2017. Macromolecular Assemblies of the Mammalian Circadian Clock. *Mol. Cell.* 67:770–782.e6.
22. Michael, A. K., J. L. Fribourgh, …, C. L. Partch. 2017. Formation of a repressive complex in the mammalian circadian clock is mediated by the secondary pocket of CRY1. *Proc. Natl. Acad. Sci. USA.* 114:1560–1565.
23. Xu, H., C. L. Gustafson, …, C. L. Partch. 2015. Cryptochrome 1 regulates the circadian clock through dynamic interactions with the BMAL1 C terminus. *Nat. Struct. Mol. Biol.* 22:476–484.
24. Lee, Y., R. Chen, …, C. Lee. 2011. Stoichiometric relationship among clock proteins determines robustness of circadian rhythms. *J. Biol. Chem.* 286:7033–7042.
25. Narasimamurthy, R., S. R. Hunt, …, D. M. Virshup. 2018. CK1delta/epsilon protein kinase primes the PER2 circadian phosphoswitch. *Proc. Natl. Acad. Sci. USA.* 115:5986–5991.
26. Hirano, A., Y. H. Fu, and L. J. Ptáček. 2016. The intricate dance of post-translational modifications in the rhythm of life. *Nat. Struct. Mol. Biol.* 23:1053–1060.
27. Meng, Q. J., E. S. Maywood, …, A. S. I. Loudon. 2010. Entrainment of disrupted circadian behavior through inhibition of casein kinase 1 (CK1) enzymes. *Proc. Natl. Acad. Sci. USA.* 107:15240–15245.
28. Lee, H., R. Chen, …, C. Lee. 2009. Essential roles of CKIδ and CKIε in the mammalian circadian clock. *Proc. Natl. Acad. Sci. USA.* 106:21359–21364.
29. Gallego, M., and D. M. Virshup. 2007. Post-translational modifications regulate the ticking of the circadian clock. *Nat. Rev. Mol. Cell Biol.* 8:139–148.
30. Vielhaber, E., E. Eide, …, D. M. Virshup. 2000. Nuclear entry of the circadian regulator mPER1 is controlled by mammalian casein kinase I epsilon. *Mol. Cell Biol.* 20:4888–4899.
31. Shinohara, Y., Y. M. Koyama, …, H. R. Ueda. 2017. Temperature-Sensitive Substrate and Product Binding Underlie Temperature-Compensated Phosphorylation in the Clock. *Mol. Cell.* 67:783–798.e20.
32. Isojima, Y., M. Nakajima, …, H. R. Ueda. 2009. CKIepsilon/delta-dependent phosphorylation is a temperature-insensitive, period-determining process in the mammalian circadian clock. *Proc. Natl. Acad. Sci. USA.* 106:15744–15749.
33. Cheong, J. K., and D. M. Virshup. 2011. Casein kinase 1: Complexity in the family. *Int. J. Biochem. Cell Biol.* 43:465–469.
34. Vielhaber, E., and D. M. Virshup. 2001. Casein kinase I: from obscurity to center stage. *IUBMB Life.* 51:73–78.
35. Görl, M., M. Merrow, …, M. Brunner. 2001. A PEST-like element in FREQUENCY determines the length of the circadian period in Neurospora crassa. *Embo J.* 20:7074–7084.
36. Kloss, B., J. L. Price, …, M. W. Young. 1998. The Drosophila clock gene double-time encodes a protein closely related to human casein kinase Iepsilon. *Cell.* 94:97–107.
37. van Ooijen, G., M. Hindle, …, A. J. Millar. 2013. Functional Analysis of Casein Kinase 1 in a Minimal Circadian System. *PLoS One.* 8:e70021.
38. Xu, Y., Q. S. Padiath, …, Y. H. Fu. 2005. Functional consequences of a CKIdelta mutation causing familial advanced sleep phase syndrome. *Nature.* 434:640–644.
39. Goldsmith, E. J., R. Akella, …, J. M. Humphreys. 2007. Substrate and docking interactions in serine/threonine protein kinases. *Chem. Rev.* 107:5065–5081.
40. Longenecker, K. L., P. J. Roach, and T. D. Hurley. 1996. Three-dimensional structure of mammalian casein kinase I: molecular basis for phosphate recognition. *J. Mol. Biol.* 257:618–631.
41. Gietzen, K. F., and D. M. Virshup. 1999. Identification of inhibitory autophosphorylation sites in casein kinase I epsilon. *J. Biol. Chem.* 274:32063–32070.
42. Johnson, L. N., M. E. Noble, and D. J. Owen. 1996. Active and inactive protein kinases: Structural basis for regulation. *Cell.* 85:149–158.
43. Venkatesan, A., J. Y. Fan, …, J. L. Price. 2019. The Circadian tau Mutation in Casein Kinase 1 Is Part of a Larger Domain That Can Be Mutated to Shorten Circadian Period. *Int. J. Mol. Sci.* 20:813.
44. Zeringo, N. A., and J. J. Bellizzi, 3rd. 2014. A PER2-derived mechanism-based bisubstrate analog for casein kinase 1epsilon. *Chem. Biol. Drug Des.* 84:697–703.
45. Xu, R. M., G. Carmel, …, X. Cheng. 1995. Crystal-Structure of Casein Kinase-1, a Phosphate-Directed Protein-Kinase. *Embo Journal.* 14:1015–1023.
46. Harold, R. L., N. K. Tulsian, …, C. L. Partch. 2024. Isoform-specific C-terminal phosphorylation drives autoinhibition of Casein kinase 1. *Proc. Natl. Acad. Sci. USA.* 121:e2415567121.

47. Philpott, J. M., A. M. Freeberg, …, C. L. Partch. 2023. PERIOD phosphorylation leads to feedback inhibition of CK1 activity to control circadian period. *Mol. Cell.* 83:1677–1692.e8.

48. Narasimamurthy, R., and D. M. Virshup. 2021. The phosphorylation switch that regulates ticking of the circadian clock. *Mol. Cell.* 81:1133–1146.

49. Masuda, S., R. Narasimamurthy, …, D. M. Virshup. 2020. Mutation of a PER2 phosphodegron perturbs the circadian phosphoswitch. *Proc. Natl. Acad. Sci. USA.* 117:10888–10896.

50. Zhou, M., J. K. Kim, …, D. M. Virshup. 2015. A Period2 Phosphoswitch Regulates and Temperature Compensates Circadian Period. *Mol. Cell.* 60:77–88.

51. Vanselow, K., J. T. Vanselow, …, A. Kramer. 2006. Differential effects of PER2 phosphorylation: molecular basis for the human familial advanced sleep phase syndrome (FASPS). *Genes Dev.* 20:2660–2672.

52. Eide, E. J., M. F. Woolf, …, D. M. Virshup. 2005. Control of mammalian circadian rhythm by CKIepsilon-regulated proteasome-mediated PER2 degradation. *Mol. Cell Biol.* 25:2795–2807.

53. Shirogane, T., J. Jin, …, J. W. Harper. 2005. SCFbeta-TRCP controls clock-dependent transcription via casein kinase 1-dependent degradation of the mammalian period-1 (Per1) protein. *J. Biol. Chem.* 280:26863–26872.

54. Ohsaki, K., K. Oishi, …, N. Ishida. 2008. The role of beta-TrCP1 and beta-TrCP2 in circadian rhythm generation by mediating degradation of clock protein PER2. *J. Biochem.* 144:609–618.

55. Francisco, J. C., and D. M. Virshup. 2024. Hierarchical and scaffolded phosphorylation of two degrons controls PER2 stability. *J. Biol. Chem.* 300:107391.

56. Toh, K. L., C. R. Jones, …, Y. H. Fu. 2001. An hPer2 phosphorylation site mutation in familial advanced sleep phase syndrome. *Science.* 291:1040–1043.

57. Xu, Y., K. L. Toh, …, L. J. Ptácek. 2007. Modeling of a human circadian mutation yields insights into clock regulation by PER2. *Cell.* 128:59–70.

58. Brennan, K. C., E. A. Bates, …, L. J. Ptáček. 2013. Casein kinase idelta mutations in familial migraine and advanced sleep phase. *Sci. Transl. Med.* 5:183ra56.

59. Rothenfluh, A., M. Abodeely, and M. W. Young. 2000. Short-period mutations of per affect a double-time-dependent step in the Drosophila circadian clock. *Curr. Biol.* 10:1399–1402.

60. Suri, V., J. C. Hall, and M. Rosbash. 2000. Two novel doubletime mutants alter circadian properties and eliminate the delay between RNA and protein in Drosophila. *J. Neurosci.* 20:7547–7555.

61. Lowrey, P. L., K. Shimomura, …, J. S. Takahashi. 2000. Positional syntenic cloning and functional characterization of the mammalian circadian mutation tau. *Science.* 288:483–492.

62. Price, J. L., J. Blau, …, M. W. Young. 1998. Double-time is a novel clock gene that regulates PERIOD protein accumulation. *Cell.* 94:83–95.

63. Ralph, M. R., and M. Menaker. 1988. A mutation of the circadian system in golden hamsters. *Science.* 241:1225–1227.

64. Kim, D. W., C. Chang, …, J. K. Kim. 2019. Systems approach reveals photosensitivity and PER2 level as determinants of clock-modulator efficacy. *Mol. Syst. Biol.* 15:e8838.

65. Kolarski, D., C. Miró-Vinyals, …, B. L. Feringa. 2021. Reversible modulation of circadian time with chronophotopharmacology. *Nat. Commun.* 12:3164.

66. Kim, J. K., D. B. Forger, …, K. M. Walton. 2013. Modeling and validating chronic pharmacological manipulation of circadian rhythms. *CPT Pharmacometrics Syst. Pharmacol.* 2:e57.

67. Long, A., H. Zhao, and X. Huang. 2012. Structural Basis for the Interaction between Casein Kinase 1 Delta and a Potent and Selective Inhibitor. *J. Med. Chem.* 55:956–960.

68. Long, A. M., H. Zhao, and X. Huang. 2012. Structural Basis for the Potent and Selective Inhibition of Casein Kinase 1 Epsilon. *J. Med. Chem.* 55:10307–10311.

69. Sprouse, J., L. Reynolds, …, G. E. Pickard. 2010. Chronic treatment with a selective inhibitor of casein kinase I δ/ε yields cumulative phase delays in circadian rhythms. *Psychopharmacology.* 210:569–576.

70. Sprouse, J., L. Reynolds, …, M. Engwall. 2009. Inhibition of casein kinase I ε/δ produces phase shifts in the circadian rhythms of Cynomolgus monkeys. *Psychopharmacology.* 204:735–742.

71. Walton, K. M., K. Fisher, …, T. T. Wager. 2009. Selective inhibition of casein kinase 1 epsilon minimally alters circadian clock period. *J. Pharmacol. Exp. Ther.* 330:430–439.

72. Badura, L., T. Swanson, …, J. Sprouse. 2007. An inhibitor of casein kinase I epsilon induces phase delays in circadian rhythms under free-running and entrained conditions. *J. Pharmacol. Exp. Ther.* 322:730–738.

73. Philpott, J. M., R. Narasimamurthy, …, C. L. Partch. 2020. Casein kinase 1 dynamics underlie substrate selectivity and the PER2 circadian phosphoswitch. *eLife.* 9:e52343.

74. Modi, V., and R. L. Dunbrack. 2022. Kincore: a web resource for structural classification of protein kinases and their inhibitors. *Nucleic Acids Res.* 50:D654–D664.

75. Gebel, J., M. Tuppi, …, V. Dötsch. 2020. p63 uses a switch-like mechanism to set the threshold for induction of apoptosis. *Nat. Chem. Biol.* 16:1078–1086.

76. Pande, V. S., K. Beauchamp, and G. R. Bowman. 2010. Everything you wanted to know about Markov State Models but were afraid to ask. *Methods.* 52:99–105.

77. Bowman, G. R., F. Noé, and V. S. Pande. 2014. An Introduction to Markov State Models and Their Application to Long Timescale Molecular Simulation. *In* Advances in Experimental Medicine and Biology, *797*. N. Rezaei, O. Steinlein, and …, R. Gerlaieds. Springer, p. 1, online resource (XII, 139 pages 65 illustrations, 48 illustrations in color.

78. Chodera, J. D., and F. Noé. 2014. Markov state models of biomolecular conformational dynamics. *Curr. Opin. Struct. Biol.* 25:135–144.

79. Husic, B. E., and V. S. Pande. 2018. Markov State Models: From an Art to a Science. *J. Am. Chem. Soc.* 140:2386–2396.

80. Shukla, D., C. X. Hernández, …, V. S. Pande. 2015. Markov State Models Provide Insights into Dynamic Modulation of Protein Function. *Acc. Chem. Res.* 48:414–422.

81. Pontiggia, F., D. V. Pachov, …, D. Kern. 2015. Free energy landscape of activation in a signalling protein at atomic resolution. *Nat. Commun.* 6:7284.

82. Malmstrom, R. D., C. T. Lee, …, R. E. Amaro. 2014. Application of Molecular-Dynamics Based Markov State Models to Functional Proteins. *J. Chem. Theory Comput.* 10:2648–2657.

83. Barros, E. P., Ö. Demir, …, R. E. Amaro. 2020. Markov state models and NMR uncover an overlooked allosteric loop in p53. *Chem. Sci.* 12:1891–1900.

84. Zimmerman, M. I., K. M. Hart, …, G. R. Bowman. 2017. Prediction of New Stabilizing Mutations Based on Mechanistic Insights from Markov State Models. *ACS Cent. Sci.* 3:1311–1321.

85. Malmstrom, R. D., A. P. Kornev, …, R. E. Amaro. 2015. Allostery through the computational microscope: cAMP activation of a canonical signalling domain. *Nat. Commun.* 6:7588.

86. Shukla, D., Y. Meng, …, V. S. Pande. 2014. Activation pathway of Src kinase reveals intermediate states as targets for drug design. *Nat. Commun.* 5:3397.

87. Bowman, G. R., E. R. Bolin, …, S. Marqusee. 2015. Discovery of multiple hidden allosteric sites by combining Markov state models and experiments. *Proc. Natl. Acad. Sci. USA.* 112:2734–2739.

88. Taylor, B. C., C. T. Lee, and R. E. Amaro. 2019. Structural basis for ligand modulation of the CCR2 conformational landscape. *Proc. Natl. Acad. Sci. USA.* 116:8131–8136.

89. Juárez-Jiménez, J., A. A. Gupta, …, J. Michel. 2020. Dynamic design: manipulation of millisecond timescale motions on the energy landscape of cyclophilin A. *Chem. Sci.* 11:2670–2680.

90. Durrant, J. D., S. E. Kochanek, …, R. E. Amaro. 2020. Mesoscale All-Atom Influenza Virus Simulations Suggest New Substrate Binding Mechanism. *ACS Cent. Sci.* 6:189–196.
91. Miao, Y., V. A. Feher, and J. A. McCammon. 2015. Gaussian Accelerated Molecular Dynamics: Unconstrained Enhanced Sampling and Free Energy Calculation. *J. Chem. Theory Comput.* 11:3584–3595.
92. Miao, Y. 2018. Acceleration of biomolecular kinetics in Gaussian accelerated molecular dynamics. *J. Chem. Phys.* 149:072308.
93. Ricci, C. G., J. S. Chen, …, G. Palermo. 2019. Deciphering Off-Target Effects in CRISPR-Cas9 through Accelerated Molecular Dynamics. *ACS Cent. Sci.* 5:651–662.
94. Wang, J., P. R. Arantes, …, Y. Miao. 2021. Gaussian accelerated molecular dynamics (GaMD): principles and applications. *Wiley Interdiscip. Rev. Comput. Mol. Sci.* 11:e1521.
95. Anandakrishnan, R., B. Aguilar, and A. V. Onufriev. 2012. H++ 3.0: automating pK prediction and the preparation of biomolecular structures for atomistic molecular modeling and simulations. *Nucleic Acids Res.* 40:W537–W541.
96. Jorgensen, W. L., J. Chandrasekhar, …, M. L. Klein. 1983. Comparison of simple potential functions for simulating liquid water. *J. Chem. Phys.* 79:926–935.
97. Maier, J. A., C. Martinez, …, C. Simmerling. 2015. ff14SB: Improving the Accuracy of Protein Side Chain and Backbone Parameters from ff99SB. *J. Chem. Theory Comput.* 11:3696–3713.
98. Wang, J., R. M. Wolf, …, D. A. Case. 2004. Development and testing of a general amber force field. *J. Comput. Chem.* 25:1157–1174.
99. Kashefolgheta, S., and A. Vila Verde. 2017. Developing force fields when experimental data is sparse: AMBER/GAFF-compatible parameters for inorganic and alkyl oxoanions. *Phys. Chem. Chem. Phys.* 19:20593–20607.
100. Case, D. A., R. M. Betz, …, P. A. Kollman. 2016. AMBER 2016. University of California.
101. Ryckaert, J.-P., G. Ciccotti, and H. J. C. Berendsen. 1977. Numerical integration of the cartesian equations of motion of a system with constraints: molecular dynamics of n-alkanes. *J. Comput. Phys.* 23:327–341.
102. Darden, T., D. York, and L. Pedersen. 1993. Particle mesh Ewald: An $N\cdot\log(N)$ method for Ewald sums in large systems. *J. Chem. Phys.* 98:10089–10092.
103. Case, D. A., R. M. Betz, …, N. Homeyer. 2017. AMBER 2017. University of California, San Francisco.
104. Scherer, M. K., B. Trendelkamp-Schroer, …, F. Noé. 2015. PyEMMA 2: A Software Package for Estimation, Validation, and Analysis of Markov Models. *J. Chem. Theory Comput.* 11:5525–5542.
105. Kozakov, D., L. E. Grove, …, S. Vajda. 2015. The FTMap family of web servers for determining and characterizing ligand-binding hot spots of proteins. *Nat. Protoc.* 10:733–755.
106. Sheffield, P., S. Garrard, and Z. Derewenda. 1999. Overcoming expression and purification problems of RhoGDI using a family of "parallel" expression vectors. *Protein Expr. Purif.* 15:34–39.
107. Delaglio, F., S. Grzesiek, …, A. Bax. 1995. NMRPipe: a multidimensional spectral processing system based on UNIX pipes. *J. Biomol. NMR.* 6:277–293.
108. Vranken, W. F., W. Boucher, …, E. D. Laue. 2005. The CCPN data model for NMR spectroscopy: development of a software pipeline. *Proteins.* 59:687–696.
109. Noé, F., H. Wu, …, N. Plattner. 2013. Projected and hidden Markov models for calculating kinetics and metastable states of complex molecules. *J. Chem. Phys.* 139:184114.
110. Pérez-Hernández, G., F. Paul, …, F. Noé. 2013. Identification of slow molecular order parameters for Markov model construction. *J. Chem. Phys.* 139:015102.
111. Nolen, B., S. Taylor, and G. Ghosh. 2004. Regulation of protein kinases; controlling activity through activation segment conformation. *Mol. Cell.* 15:661–675.
112. Tuppi, M., S. Kehrloesser, …, V. Dötsch. 2018. Oocyte DNA damage quality control requires consecutive interplay of CHK2 and CK1 to activate p63. *Nat. Struct. Mol. Biol.* 25:261–269.
113. Lee, C., D. R. Weaver, and S. M. Reppert. 2004. Direct association between mouse PERIOD and CKIepsilon is critical for a functioning circadian clock. *Mol. Cell Biol.* 24:584–594.
114. Konopka, R. J., and S. Benzer. 1971. Clock mutants of Drosophila melanogaster. *Proc. Natl. Acad. Sci. USA.* 68:2112–2116.
115. Johnson, J. L., T. M. Yaron, …, L. C. Cantley. 2023. An atlas of substrate specificities for the human serine/threonine kinome. *Nature.* 613:759–766.
116. Marzoll, D., F. E. Serrano, …, M. Brunner. 2022. Casein kinase 1 and disordered clock proteins form functionally equivalent, phospho-based circadian modules in fungi and mammals. *Proc. Natl. Acad. Sci. USA.* 119:e2118286119.

Biophysical Journal, Volume *124*

Supplemental information

Markovian state models uncover casein kinase 1 dynamics that govern circadian period

Clarisse Gravina Ricci, Jonathan M. Philpott, Megan R. Torgrimson, Alfred M. Freeberg, Rajesh Narasimamurthy, Emilia Pécora de Barros, Rommie Amaro, David M. Virshup, J. Andrew McCammon, and Carrie L. Partch

Supplemental Materials and Methods

Markovian State Models uncover Casein Kinase 1 dynamics that govern circadian period

Clarisse Gravina Ricci,[1*] Jonathan M. Philpott,[2] Megan R. Torgrimson,[2] Alfred M. Freeberg,[2] Rajesh Narasimamurthy,[3] Emilia Pécora de Barros,[1] Rommie Amaro,[1] David M. Virshup,[3,4] J. Andrew McCammon,[1] and Carrie L. Partch[2,5,6*]

[1] Department of Chemistry and Biochemistry, University of California San Diego, San Diego, California, United States.

[2] Department of Chemistry and Biochemistry, University of California Santa Cruz, Santa Cruz, California, United States.

[3] Program in Cancer and Stem Cell Biology, Duke-NUS Medical School, Singapore, Singapore.

[4] Department of Pediatrics, Duke University Medical Center, Durham, North Carolina, United States.

[5] Center for Circadian Biology, University of California San Diego, San Diego, California, United States.

[6] Howard Hughes Medical Institute, University of California Santa Cruz, Santa Cruz, California, United States.

* Corresponding authors: cla.g.ricci@gmail.com (C.G.R.), cpartch@ucsc.edu (C.L.P.)

A. Molecular dynamics simulations of apo proteins

To start the molecular dynamics simulations of WT and *tau* CK1 that would be used to build the MSMs, we extracted conformations from Gaussian accelerated MD simulations as described previously [1] and illustrated in Figure S1. We wanted these initial structures to capture different conformations of the activation loop, so we applied principal component analysis (PCA) to the backbone atoms of the activation segment and its flanking regions (residues 151 to 185, Figure S1A). All the GaMD trajectories from WT or *tau* CK1 were concatenated together so that the resulting vectorial space (PC1 vs PC2) is the same for both systems. We then selected 10 conformations located at different regions of the essential space describing the activation loop dynamics and used them as starting points for our first round of conventional MD simulations (Figure S1B). For each initial structure, we launched three independent MD replicas (with different initial velocities) for 300 ns, totalizing 9us for each system (WT or *tau*). After these finished running, we randomly selected 20 more structures from the new trajectories and re-launched two independent MD replicas for 300 ns, adding 12us to the total time simulated for each system.

All systems were solvated with TIP3P water molecules [2] in cubic boxes with at least 15 Angstroms between the protein and the box boundaries. The simulation boxes were neutralized with Na+ or Cl- counterions. Protein and ions were parametrized with Amber ff14SB forcefield [3], while parameters for

SO_4^{2+} (the anion bound to the first anion binding pocket in the WT CK1) were obtained from the Generalized Amber Force Field (GAFF) [4] and adjusted as previously proposed [5].

Conventional MD simulations were performed with AMBER 16 [6] in the NVT regime, with a time step of 2fs. The PME method [7] was used to calculate electrostatic interactions using periodic boundary conditions. A 12 Angstroms cutoff was used to truncate non-bonded short-range interactions. The simulation temperature was maintained at 300 K with using the Langevin thermostat and a collision frequency of 2 ps^{-1}. No pressure coupling was applied as the boxes had been previously equilibrated. All bonds involving hydrogen were constrained using SHAKE.

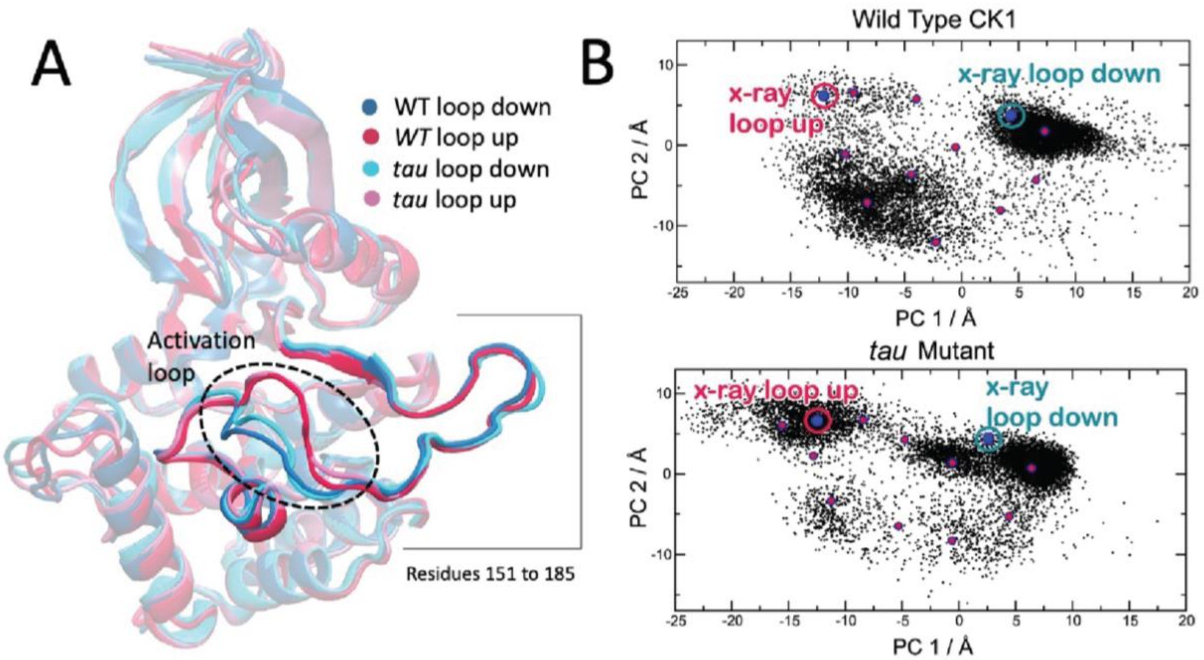

Figure S1. Selection of conformations from previous GaMD simulations as starting points for the MD simulations used to generate MSMs. A) Region of CK1 selected for Principal Component Analysis. B) Conformations from GaMD simulations selected for starting conventional simulations (pink dots).

B. Markovian State Models (MSMs)

Main (distance-based) model

Before building the MSMs, we used root mean square fluctuation (RMSF) profiles to identify the most flexible or rigid regions of the proteins (Figure S2A). We then selected 9 pair-wise distances involving the activation loop (Leu173) and the C-terminal portion of L-EF (T220). All these distances involved functionally important residues of protein, some of them flexible (S19 at the Gly-rich loop and T44 at the L-3A loop) and some of them rigid (D128 in the HRD motif and G151 in the DFG motif) to provide good anchor points. Pair-wise distances between these residues were calculated based on the positions of the α-carbons accumulated over WT and *tau* trajectories and used as input features for time-lagged Independent Component Analysis (tICA) [8]. tICA essentially identifies the linear combination of features

that best describes the *slowest* modes of motions of the system. The resulting orthogonal components (or TICs) are ranked by their associated implied timescales (from slowest to fastest). Figure S2B shows that the slowest mode of motion (TIC 1) consistently involves Leu173 at the activation loop, while the second slowest motion (TIC 2) consistently involves T220 at L-EF. In particular, the feature that most contributes to TIC 1 is the distance between Leu173, in the activation loop, and G151, which is part of the DF<u>G</u> motif, a rigid region of the protein. These results show that rearrangement of the activation loop is the slowest mode of motion in our model, even though L-EF displays higher amplitude of motion as measured by atomic fluctuations (RMSF). Based on the contributions of each feature to the TIC components (Figure S3B), we figured that the motions displayed by the activation loop (1st TIC) and L-EF (2nd TIC) are well captured by the 5 pair-distances distances involving Leu173 (Figure S3C), so we decided to use these as input to construct our main MSM.

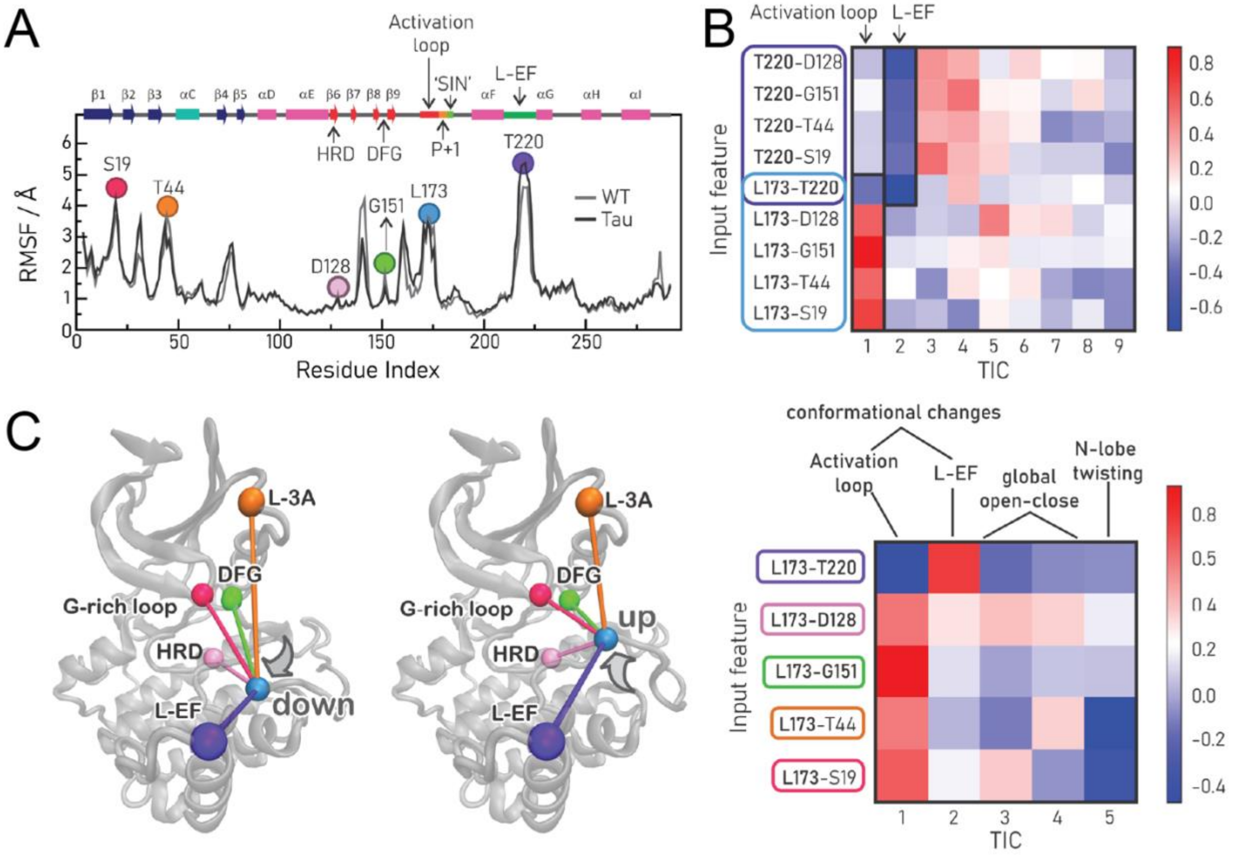

Figure S2. Selection of distances for the MSM model. A) Backbone RMSF with respect to average structure for concatenated WT or *tau* trajectories. Alignment was performed with the rigid core of the enzyme (residues 85 to 165). The residues whose pair wise distances were selected as input features are highlighted. B) tICA results showing the correlation of input features with each TIC component (1st and 2nd are highlighted). C) Final pair wise distances selected for tICA and MSM construction.

We used PyEmma [9] version 2.5.7 to process the trajectories and to build, validate and analyze the MSMs. Discretization of the conformational space was performed for WT and *tau* separately using k-means clustering, with k = 200 for both systems. The corresponding implied timescale plots are shown in Figure

S3A. We then used Hidden Markov Models to coarse-grain the MSMs into fewer metastable states that are more amenable to human interpretation. Metastable states represent kinetically distinct conformations separated by high-energy barriers (or slow motions). We decided on the number of metastable states for each system based on spectral analysis of the tICA modes of motion (Figure S3B) [10, 11]. For both WT and *tau*, we observed a spectral gap between the 2nd and 3rd timescales, indicating the dominance of two slow modes of motion and three well separated minima in the conformational landscape, so we coarse-grained the MSM with three metastable states for both systems. Clustering of microstates into metastable states was performed with the PCCA++ algorithm [12-14]. The results are shown in Figure S4A, along with the visualization of the 2nd and 3rd eigenvectors of the transition matrix, which represent the two slowest modes of motion (the 1st eigenvector corresponds to the equilibrium distribution) (Figure S4B). We then constructed Bayesian Hidden Markov Models (HMSM) [15, 16], with n = 3 and lag time (τ) of 3 ns. The quality of the coarse-grained HMSM was verified by ITS plots (Figure S5A) and Chapman-Kolmogorov (CK) tests (Figure S5B,C), with 95% confidence intervals calculated using a Bayesian sampling scheme. These are the models presented and discussed in the main manuscript.

To characterize the conformations trapped in each metastable state, we extracted 5000 representative structures with at least 70% of assigned membership. To describe the activation loop, we measured the α–carbon distance between Leu173, in the activation loop, and Leu152 (located at a rigid region of the protein) (Figure S6A). To characterize the conformational state of L-EF, we measured the RMSD of this region with respect to the crystallographic structure (Figure S67). Based on this analysis we classified the metastable states as described in Table S1. Mean first passage times (MFPTs) between states are reported in Table S2.

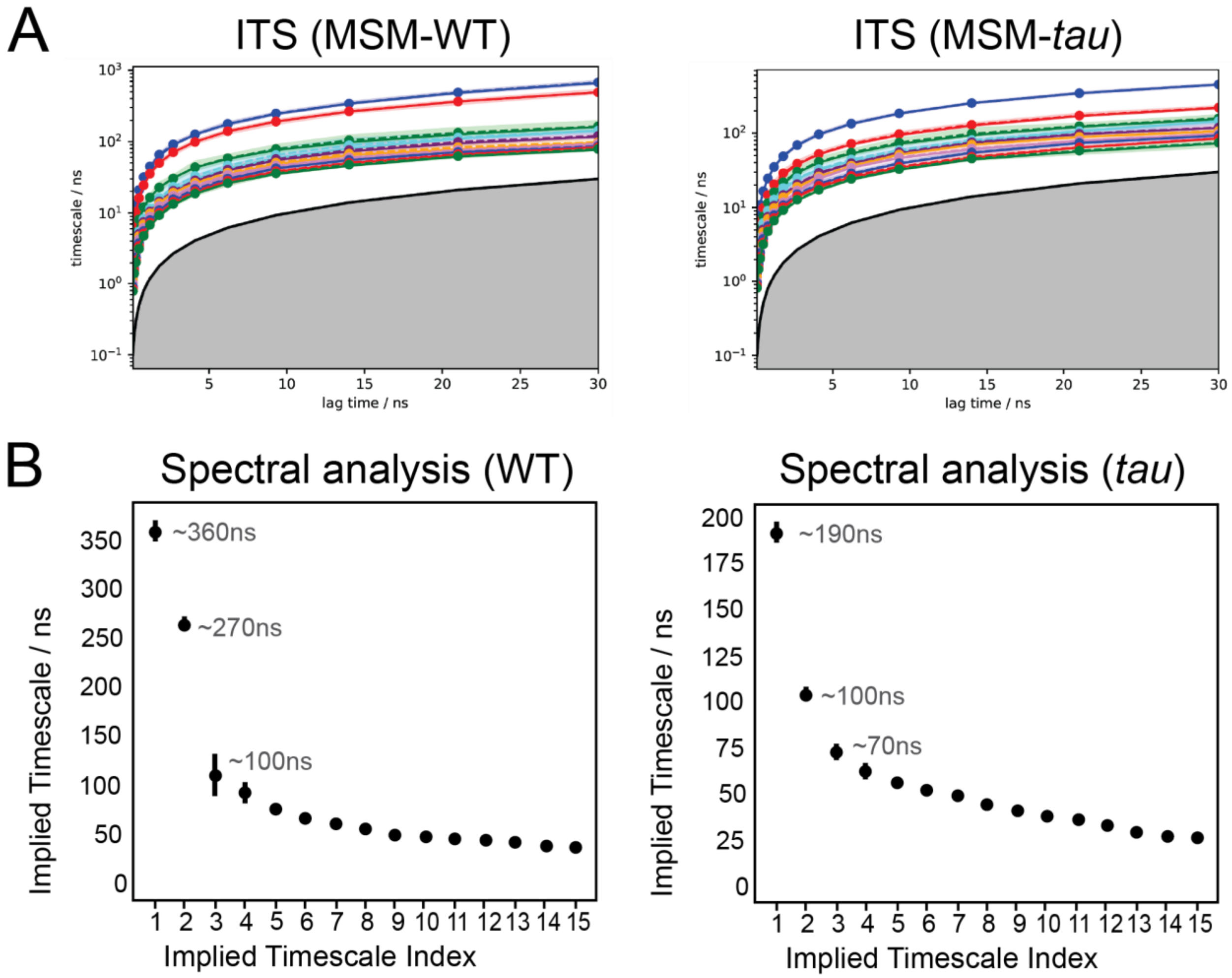

Figure S3. Analysis of the MSM model for coarse-graining. A) Convergence of MSM implied timescale plots. The grey area marks the lower limit of timescales that can be resolved for each lag time. Colored shaded areas represent 95% confidence intervals estimates from Bayesian-sampled transition matrices. Dashed lines represent the mean of Bayesian MSMs. B) Spectral analysis of implied timescales.

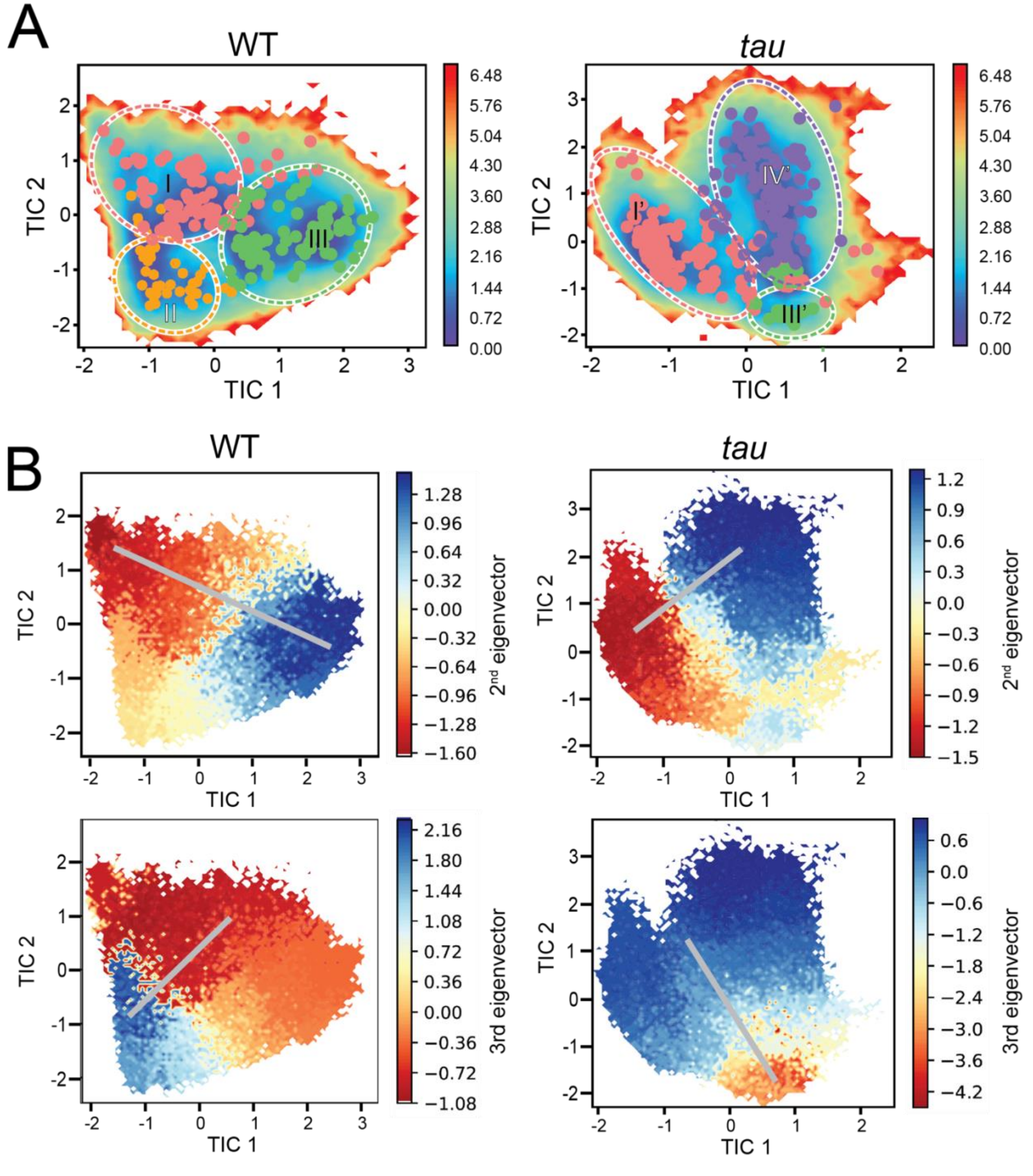

Figure S4. Analysis of metastable states. A) Free energy landscapes (FEL) superimposed with microstate clusters assigned to metastable states (I/I', II, III/III', and IV'). FELs were obtained by re-reweighting the trajectory frames with the stationary probability distribution (1st eigenvector) derived from the MSM transition matrix. B) Probability shifts associated with the 2nd and 3rd eigenvectors, which represent the 1st and 2nd slowest conformational changes in the system.

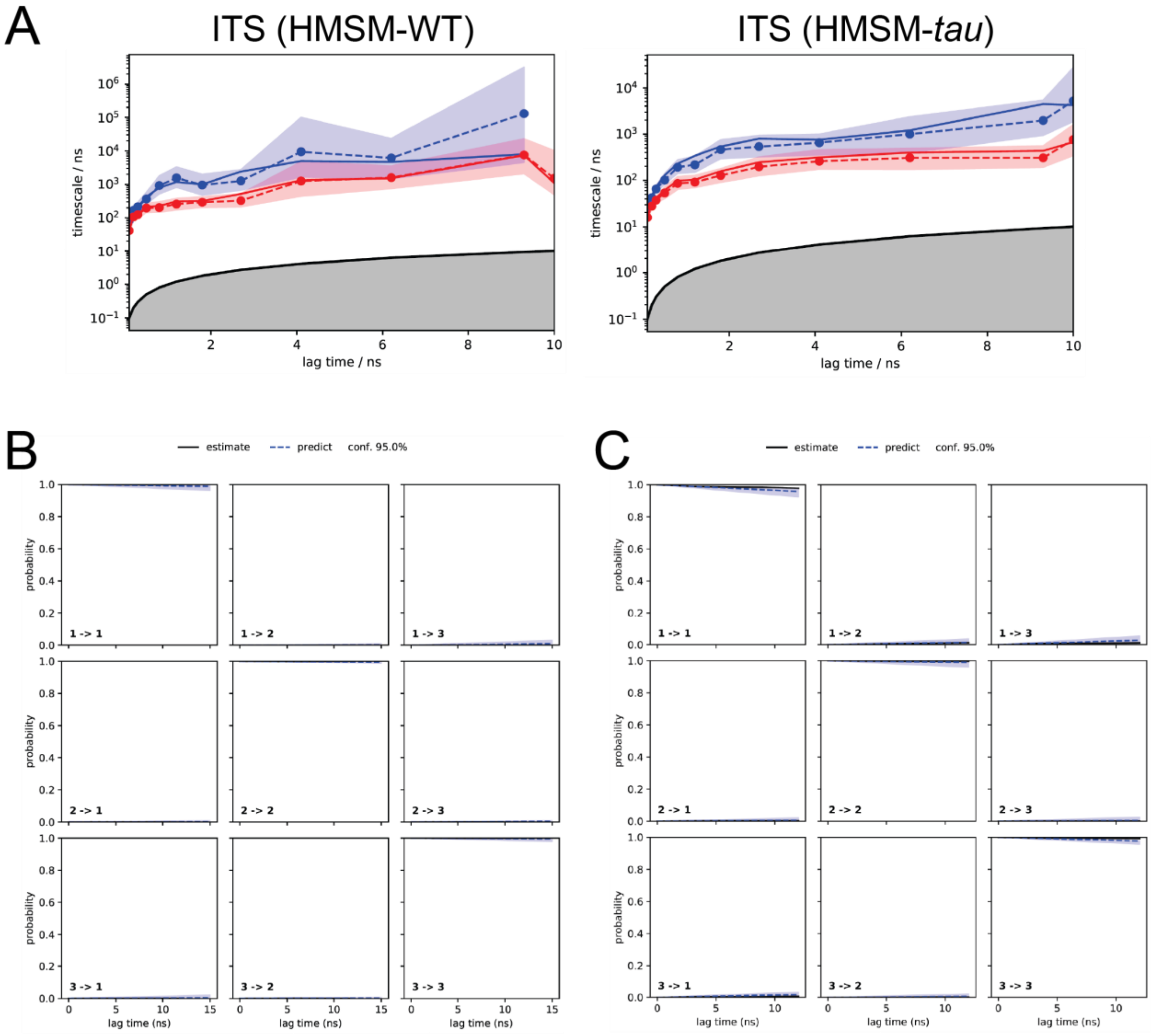

Figure S5. Quality assessment of the model. A) Convergence of ITS plots for coarse-grain HMSM. B,C) Chapman-Kolmogorov (CK) tests for HMSMs obtained for WT CK1 (B) and *tau* mutant (C). Shaded areas in the plots represent 95% confidence intervals estimated from Bayesian-sampled transition matrices and dashed lines represent the mean of Bayesian MSMs.

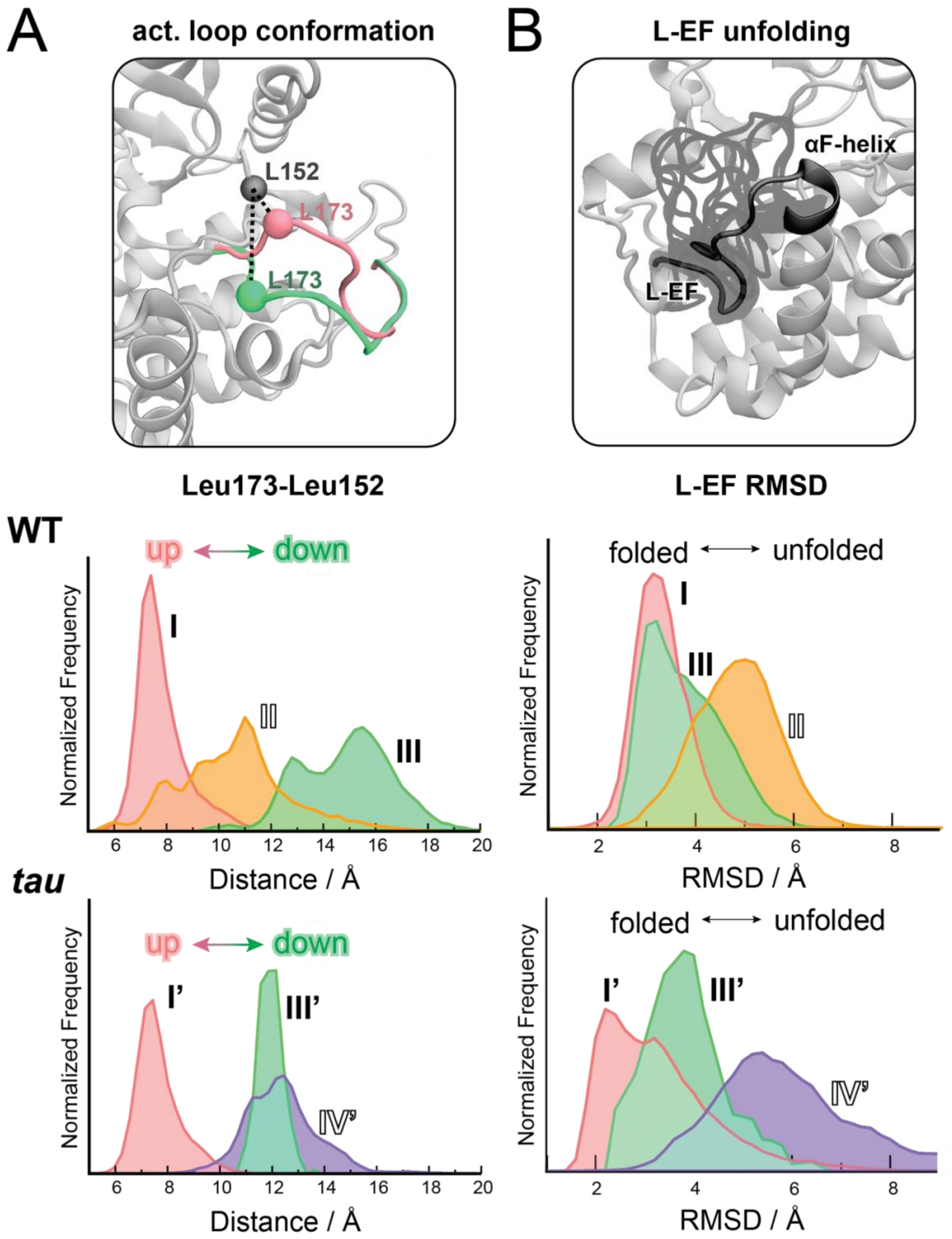

Figure S6. Characterization of meta-stable states in terms of the conformation of the activation loop. A) The conformation of the activation loop (cα distance between Leu173 and Leu152) and (B) conformational state of L-EF (high RMSD values indicate unfolding) for WT (top) and *tau* mutant (bottom). Histograms are normalized and do not reflect the relative populations of each state. Distances were calculated between the γ-carbon atom of Leu^{173} and the α-carbon of Leu^{152}. RMSDs were calculated for the backbone of residues 212 to 226 (loop EF and top of helix F), after alignment was performed for residues 85 to 165 with respect to the average structure.

Table S1. Classification of main metastable states

Metastable state	% population in WT	% population in *tau*	Conformation of the activation loop	L-EF conformational state
I/I’	34 +- 25	48 +- 9	Up	Folded
II	19 +- 14	---	Intermediate	Unfolded
III/III’	47 +- 21	20 +- 6	Down	Folded
IV’	---	32 +- 8	Down	Unfolded

Table S2. MFPT between metastable states from main HMSM

Transitions in WT	MFPT / µs	Transitions in *tau*	MFPT / µs
I → II	10.9	I’ → III’	1.6
II → I	13.9	III’ → I’	1.2
I → III	8.8	I’ → IV’	1.7
III → I	14.9	IV’ → I’	1.4
II → III	3.3	III’ → IV’	0.9
III → II	6.5	IV’ → III’	0.9

Gly^{175} (torsional) model

The second MSM model was constructed with input features based solely on the backbone angles of Gly^{175} (Figure S7A) since this residue was hypothesized to work as a hinge controlling the conformation of the activation loop [1]. Discretization of Gly^{175} configurational space was performed for WT and *tau* separately, using k-means clustering with k = 200 for both systems. Converged ITS plots revealed a gap between the 1st and 2nd timescales in WT and between the 2nd and 3rd timescales in the *tau* mutant (Figure S7B). We thus clustered the microstates into 2 metastable states for the WT and 3 metastable states for the *tau* mutant (Figure S7C). HMSM were generated with τ=20ns and validated as shown in Figure S7 (panels D-F).

The resulting HMSMs are displayed in Figure S8A and detailed in Tables S3 and S4. Conformational characterization of metastable states (Figure S8B) reveal that the torsional models fail to separate the two conformations of the activation loop. This is especially the case of WT, where metastable states A and B contain both ‘down’ and ‘up’ conformations within the same state. The difference between these two states is that in state A the activation loop can also adopt intermediate conformations between ‘up’ and ‘down’. For the *tau* mutant, the torsional model performs slightly better: while the most populated state (B’) still mixes ‘up’ and ‘down’ conformations, states C’ and D’ consist of (mainly) ‘loop up’ and ‘loop down’ conformations, respectively.

We next plotted representative structures of the metastable states in the Ramachandran space of Gly^{175} (Figure S9A). To investigate whether the configuration of Gly^{175} is correlated with the conformation of the activation loop, we also colored the Ramachandran plots according to the distance between Leu^{173} and Leu^{152} (Figure S9B). Visual inspection of these plots reveals no clear correlations in the WT but suggests a moderate correlation between the conformation of the activation loop and ϕ^{Gly175} in *tau*. To verify this, we performed a linear regression between ϕ^{Gly175} and the distance between Leu^{173} and Leu^{152}, as shown in Figure S9C. The resulting correlation coefficients confirm a weak correlation between the ϕ^{Gly175} and the conformation of the activation loop in the *tau* mutant (r=0.5), with negative values of ϕ^{Gly175} favoring

‘loop up’ conformations and positive values of ϕ^{Gly175} favoring ‘loop down’ conformations. In the WT enzyme, this correlation is not significant (r=0.36).

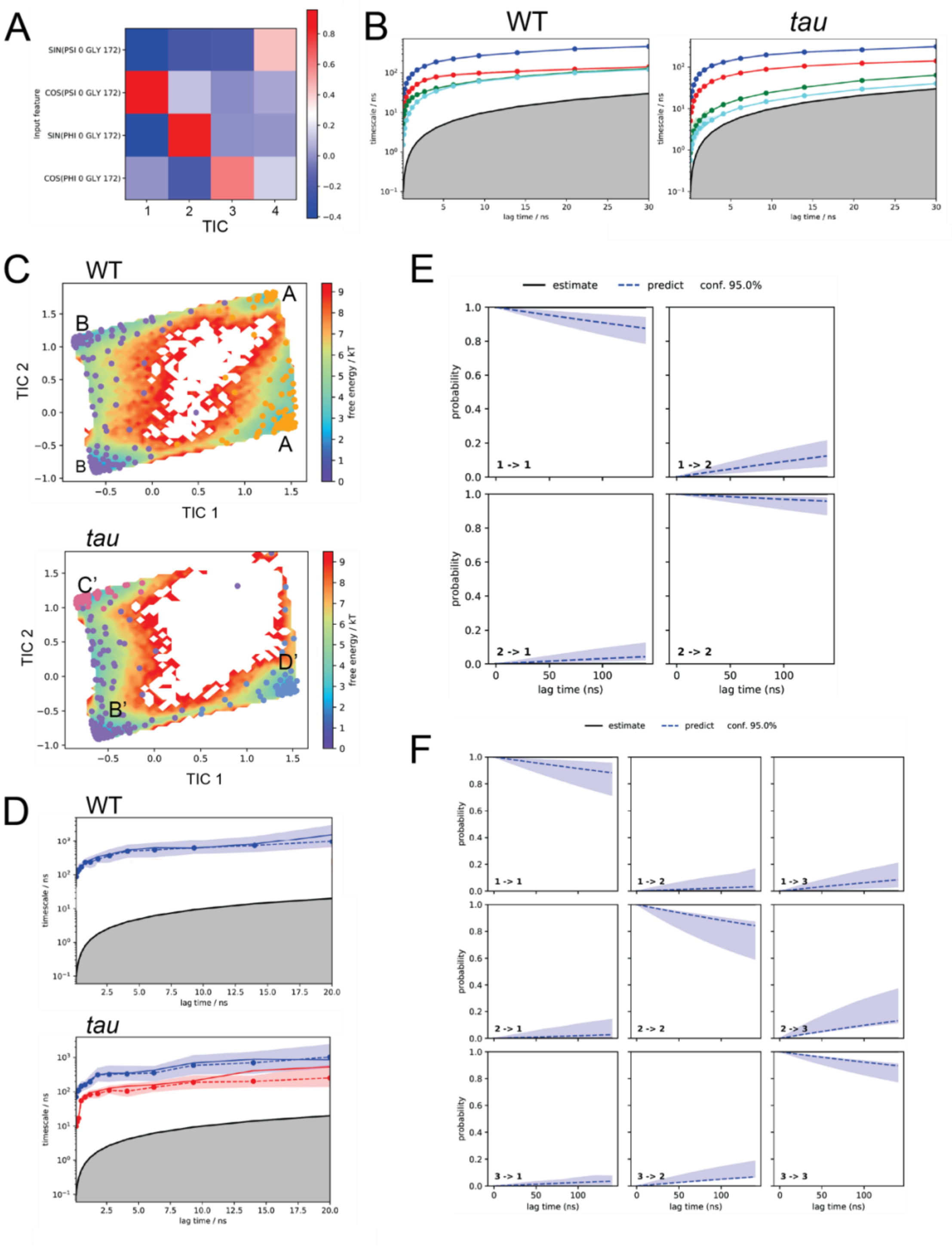

Figure S7. MSM model based on Gly^{175} torsions. A) tICA results using Gly^{175} backbone angles as input features. B) Convergence of MSM implied timescale plots. The grey area marks the lower limit of timescales that can be resolved for each lag time. Colored shaded areas represent 95% confidence intervals estimates from Bayesian-sampled transition matrices. Dashed lines represent the mean of Bayesian MSMs. C) Resulting free energy landscapes in terms of the slowest tICA components, with microstates clustered into meta-stable states (identified by letters). D) Convergence of ITS plots for coarse-grain HMSM. E,F) Chapman-Kolmogorov (CK) tests for HMSMs obtained for WT CK1 (E) and *tau* mutant (F). Shaded areas in the plots represent 95% confidence intervals estimated from Bayesian-sampled transition matrices and dashed lines represent the mean of Bayesian MSMs.

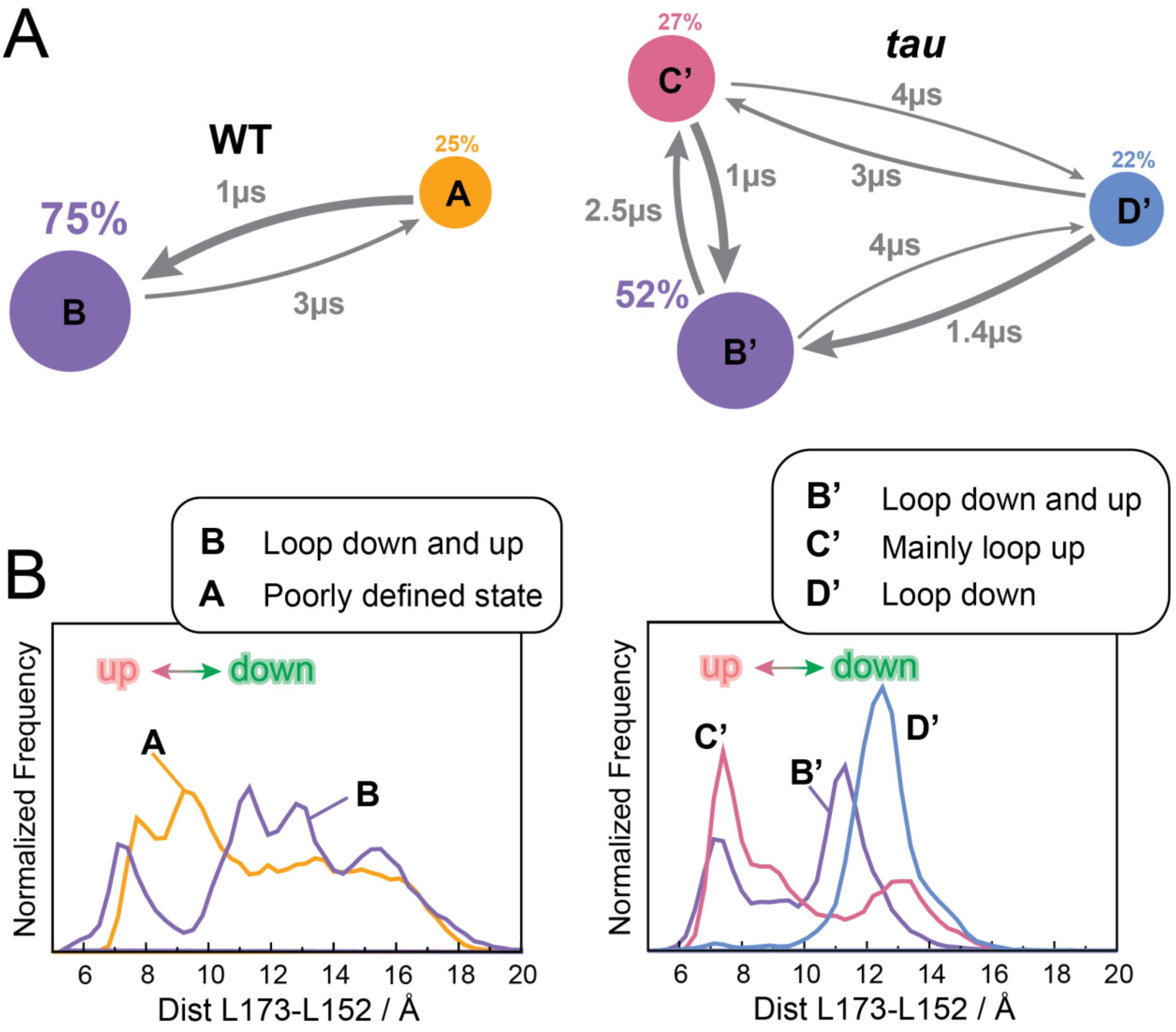

Figure S8. Characterization of the Gly^{175} MSM model in terms of the conformation of the activation loop. A) Equilibrium populations of meta-stable states and MFPTs between states for the WT (left) and *tau* (right) protein systems. Area of the circles is proportional to the equilibrium population and thickness of the arrows is proportional to transition rates between states. The numbers next to the arrows indicate MFPTs. B) Characterization of meta-stable states in terms of the conformation of the activation loop (α-carbon distance between Leu^{173} and Leu^{152}) for the WT (left) and *tau* (right) protein systems. Histograms are normalized and do not reflect the relative populations of each state. Distances were calculating between the γ-carbon atom of Leu^{173} and the α-carbon of Leu^{152}.

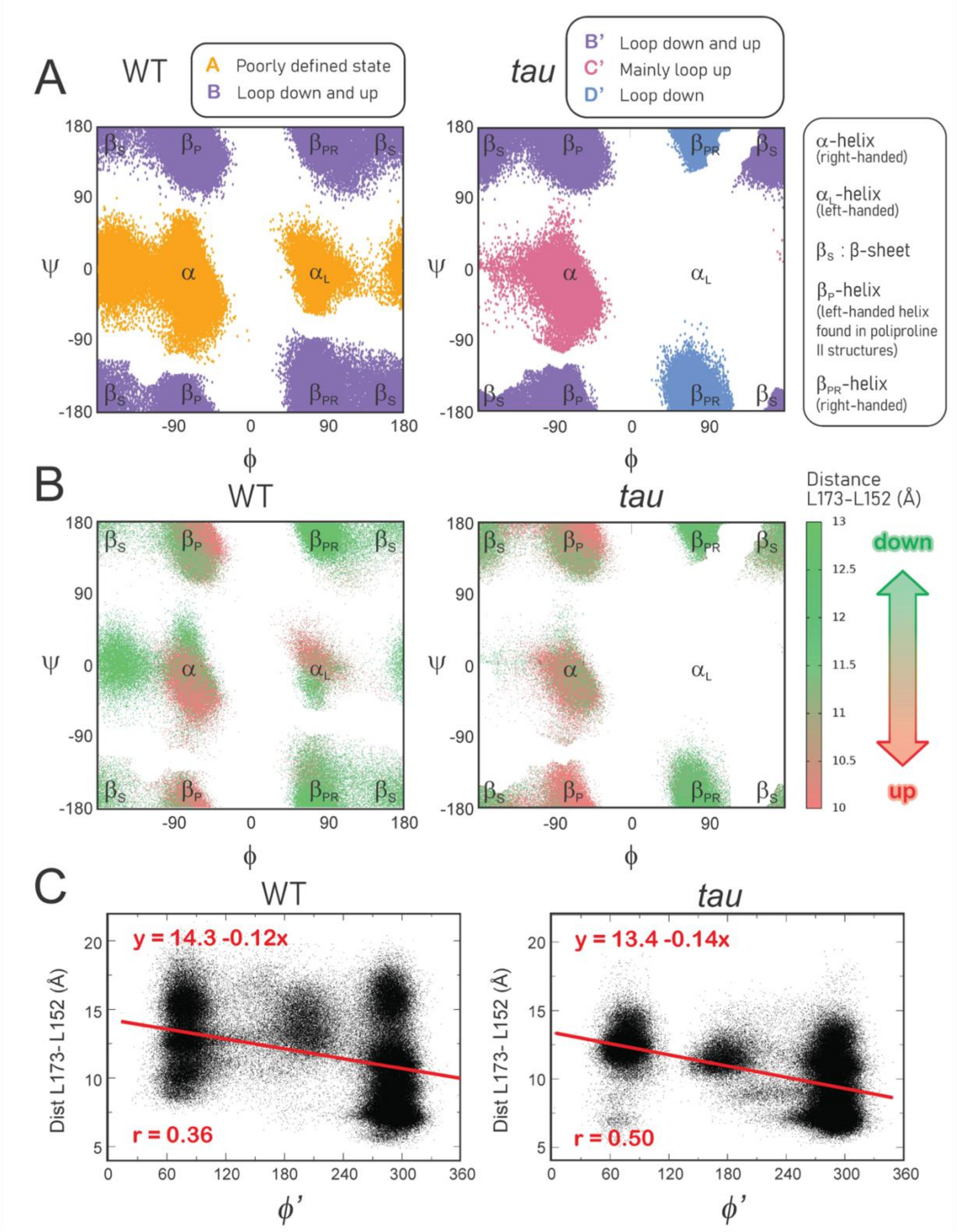

Figure S9. Meta-stable states produced by the Gly^{175} MSM model do not correlated with the conformation of the activation loop. A) Gly^{175} Ramachandran space colored according to metastable state assignment for WT (left) and *tau* (right). Low energy regions are indicated as described previously [17]. B) Gly^{175} Ramachandran space colored according the (cα) distance between Leu^{173} and Asp^{152}. C) Linear regression between ϕ^{G175} and the (cα) distance between Leu^{173} and Leu^{152}.

Table S3. Classification of torsional HMSM metastable states

Metastable state	% population in WT	% population in *tau*	Conformation of the activation loop
A	25 +- 10	---	Poorly defined
B/B'	75 +- 10	52 +- 13	Up and down
C	---	27 +- 10	Mainly up
D	---	21 +- 15	Down

Table S4. MFPT between metastable states from torsional HMSM

Transitions in WT	MFPT / µs	Transitions in *tau*	MFPT / µs
A → B	1.1	B'→ C'	2.5
B → A	3.1	C'→ B'	1.2
		B'→ D'	4.1
		D'→ B'	1.4
		C'→ D'	4.3
		D → C'	2.9

C. Molecular models of CK1 bound to FASP peptide

Initial model based on TAp63α-CK1 complex

To model the interaction between FASP and CK1, we used the x-ray structure of CK1 bound to a double phosphorylated TAp63α peptide as template (PDB 6RU7, chains A and C) [18]. Alignment between FASP and TAp63α peptide sequences is shown in Figure S10A. In this template, CK1 displays the activation loop in the 'loop down' conformation, which is the preferred conformation in the wild-type enzyme according to our MSMs. Considering that FASP is the preferred substrate over the pD, it is reasonable to assume that FASP binds to the most common 'loop down' conformation. Moreover, both the FASP priming motif and the TAp63a peptide in the template structure display a valine at position +1, which, in the template structure, interacts with Leu173 in the 'loop down' conformation.

To model the interactions prior to the priming event, the original ADP molecule in the template structure was computationally replaced by an ATP molecule extracted from an ATP-bound PKA structure (PDB 1ATP), after the active site of the two kinases were superimposed with Lovoalign [19]. We also removed any crystallographic water and ions and manually added Mg^{2+} ions coordinated to the ATP molecule as shown in Figure S10B. The template was processed with Maestro to have hydrogens added, bond orders assigned, and atomic positions optimized. We then mutated the sidechains one at a time (from N- to C-terminal), minimizing the structure after each mutation, until the bound peptide matched the FASP sequence (Figure S10B).

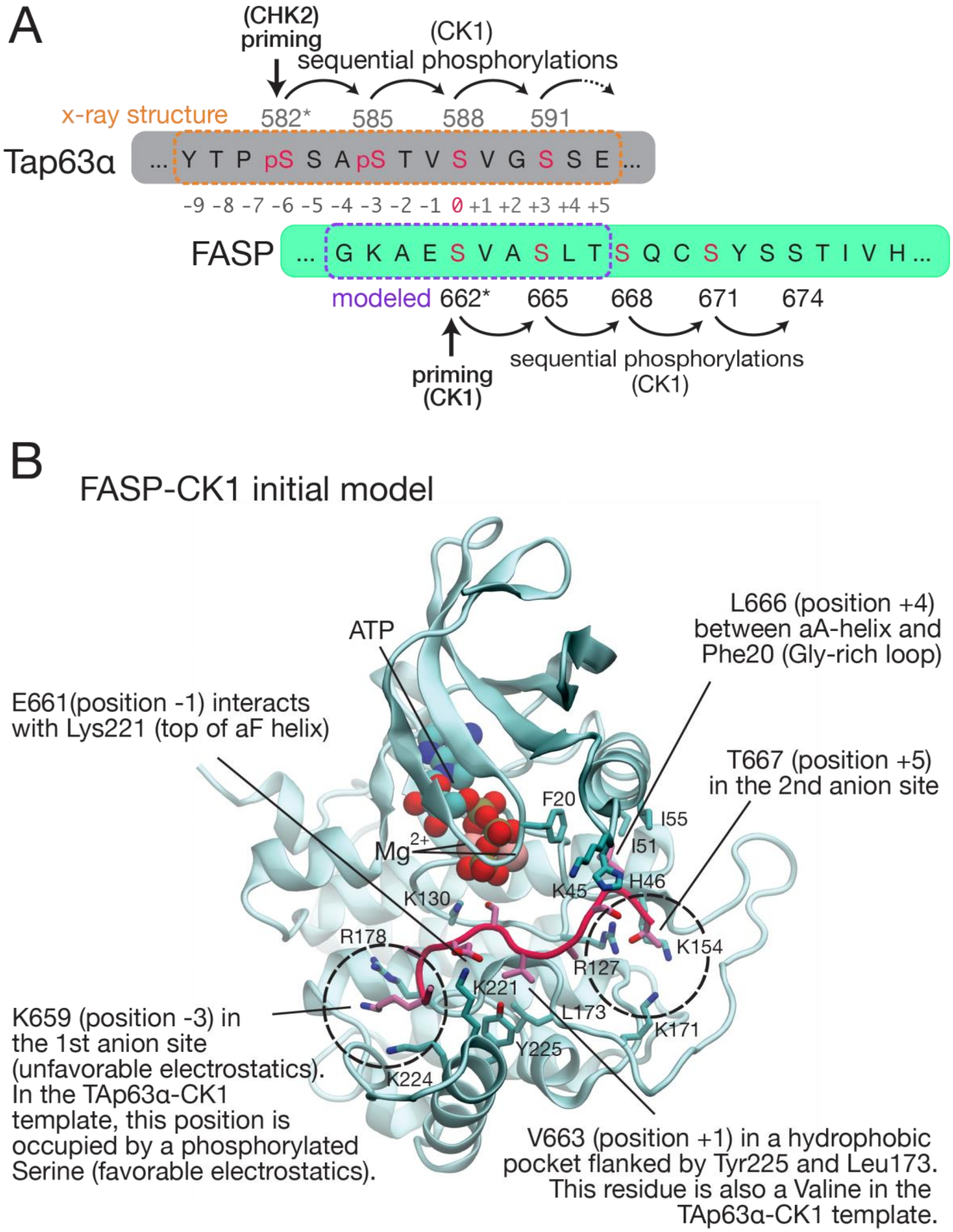

Figure S10. Model used as a starting point for MD simulations A) Alignment between TAp63α peptide and the priming region of human PER2 FASP. B) Molecular model of FASP-CK1 based on PDB 6RU7.

Relaxation of the model with Gaussian accelerated MD simulations

To avoid spurious electrostatic interactions during the MD simulations, the N- and C-terminals of the FASP peptide were capped with acetyl (ACE) and amide (NME) capping groups, respectively. Prior to simulations, hydrogen atoms were reassigned with PROPKA [20, 21] at pH 7.0. Parameters for protein residues were obtained from AMBER ff14SB force field [3] while parameters for ATP were obtained as previously described [22].To allow the system to relax and to accelerate the exploration of the binding landscape, we initially ran 10 independent replicas of GaMD simulations [23] starting with different random velocities. Each replica was simulated for 100 ns, totaling 2µs of accelerated simulations. The simulations were performed with AMBER 17 [24] using a time step of 1 fs. Equilibration and simulation protocols are summarized in Table S5.

Table S5. GaMD equilibration and simulation protocol.

Stage	Position restraints	k (kcal/mol/Å^2)	T(K)	P(20)	Steps	Time(ns)
Equilibration						
Minimization 1	CK1, FASP, ATP, Mg^{2+} (a.a)	500	---	---	2x10^3	---
Minimization 2	CK1 and FASP (a.a)	500	---	---	1x10^3	---
Minimization 3	CK1 (a.a.) and FASP (bb.)	500	---	---	1x10^3	---
Minimization 4	CK1 and FASP (bb.)	500	---	---	1x10^3	---
Minimization 5	CK1 (bb.)	500	---	---	1x10^3	---
Minimization 6	---	---	---	---	1x10^3	---
Thermalization	CK1, FASP, ATP, Mg^{2+} (a.a.)	30	300	---	25x10^3	0.05
Density equil.	CK1, FASP, ATP, Mg^{2+} (a.a.)	30	300	1	5x10^5	1
GaMD preparation						
cMD prep.	---	---	300	---	2x10^6	4
GaMD prep.	---	---	300	---	24x10^6	48
Production						
GaMD (x10 replicas)	---	---	300	---	1000x10^6	200 (x10)

a.a.: all atoms; bb.: backbone

The resulting GaMD simulations produced a variety of transient binding modes, many resulting in partial dissociation of FASP. The high flexibility of FASP in these simulations suggests that our initial model lies in an unstable region of the binding free energy landscape (Figure S11A). This is not surprising considering our model was built based on a primed peptide (Tap63α) with a phosphate group (position -3) inserted in the first anion site, whereas in the FASP model this position is occupied by a positively charged lysine (see Figure S10B). In a significant portion of the trajectories, however, FASP rearranged into stably bound conformations and, in 14% of these, it adopted conformations prone to catalysis, with the oxygen of S662 near the γ phosphorous atom of ATP (Figure S11B). During the GaMD simulations, the activation loop remained mostly in the 'down' conformation and was particularly stable in the trajectories leading to bound states (replicas 1, 2 and 5 in Figure S11C).

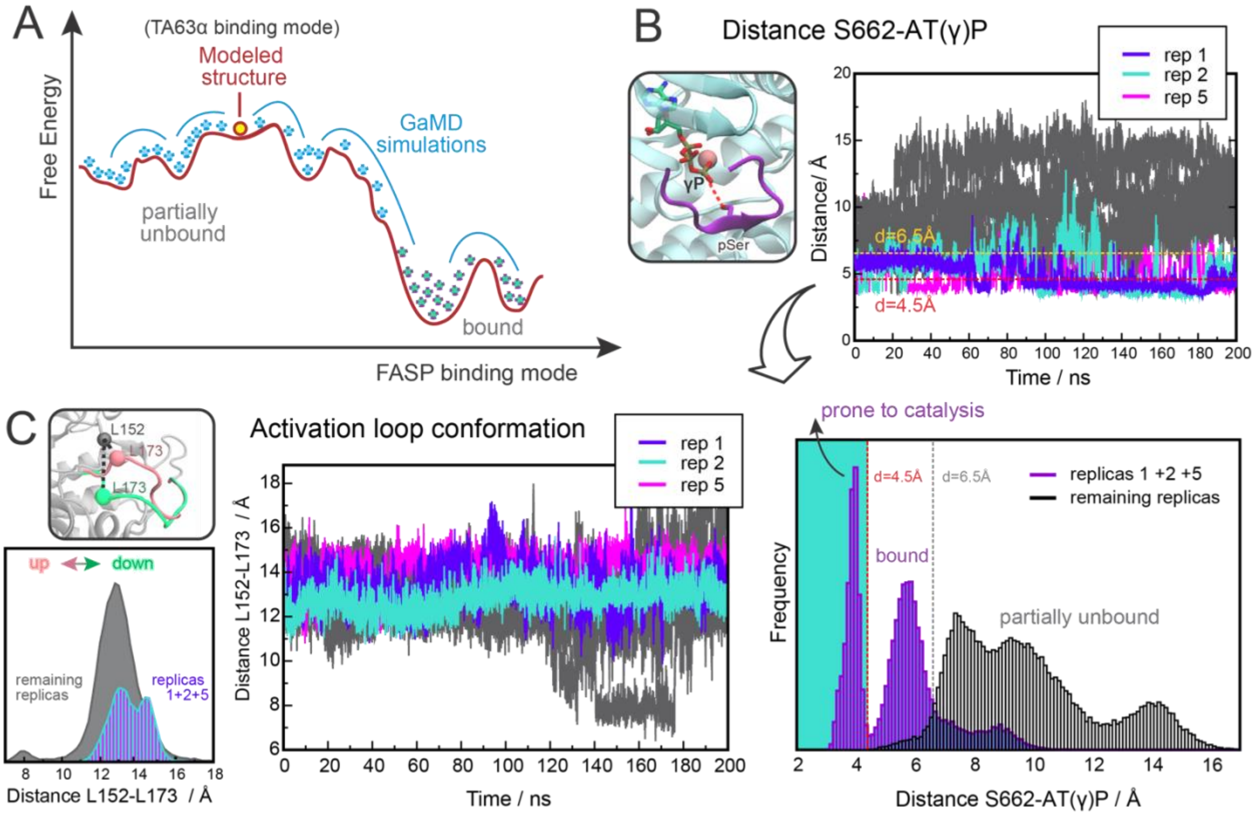

Figure S11. GaMD model of FASP-bound CK1. A) Schematic representation of FASP-CK1 binding landscape based on GaMD trajectories. B) Temporal evolution (top) and corresponding distribution (bottom) of the distance between S662 (γ oxygen) and the γ phosphorous atoms from ATP. C) Temporal evolution (right) and corresponding distribution (left) of the distance between γ-carbon atom of Leu^{173} and the α-carbon of Leu^{152}. Trajectories that produced bound states (replicas 1, 2, and 5) are highlighted.

Final refinement with conventional MD simulations

To refine the binding mode of FASP, we selected 10 equally spaced conformations from trajectories that produced stably bound states as starting points for cMD simulations (Figure S12A). Based on these starting points, we ran 50 independent replicas (40 ns each), totaling 2µs of simulation time (Figure S12B). All replicas started with different initial velocities and used the same force field and parameters as described for the GaMD simulations, but without any boosting potential. Although FASP retained mobility freedom in the cMD simulations, it remained bound to CK1 and often sampled conformations that are prone to catalysis (Figure S12C). A detailed look at FASP dynamics during individual replicas (Figure S12D) reveals that FASP can rearrange in and out of its 'catalytically prone' binding mode within the nanosecond timescale.

Visual inspection of frames belonging to the 'catalytically prone' state reveals that the C-terminal portion of the FASP peptide retains high mobility freedom and does not engage in specific interactions with CK1. V663 at position +1 (relative to the priming serine) remains stably bound in the hydrophobic pocket between helix αF (Tyr^{225}) and the activation loop (Leu^{173}) (Figure S13A). The first anion binding pocket is partially occupied by E661 at position -1, which engages in electrostatic interactions with one of the positively charged clamps forming this site (Arg^{178}, in the P+1 loop) (Figure S13B). After moving out of the first anion site, K659 at position -3 engages in electrostatic interactions with the β phosphate of ATP and,

at times, with Asp[132] (Figure S13C). Observations for the catalytically prone conformations were confirmed by RMSF and distance analyses (Table S6) reported in Figure 4 of the main manuscript.

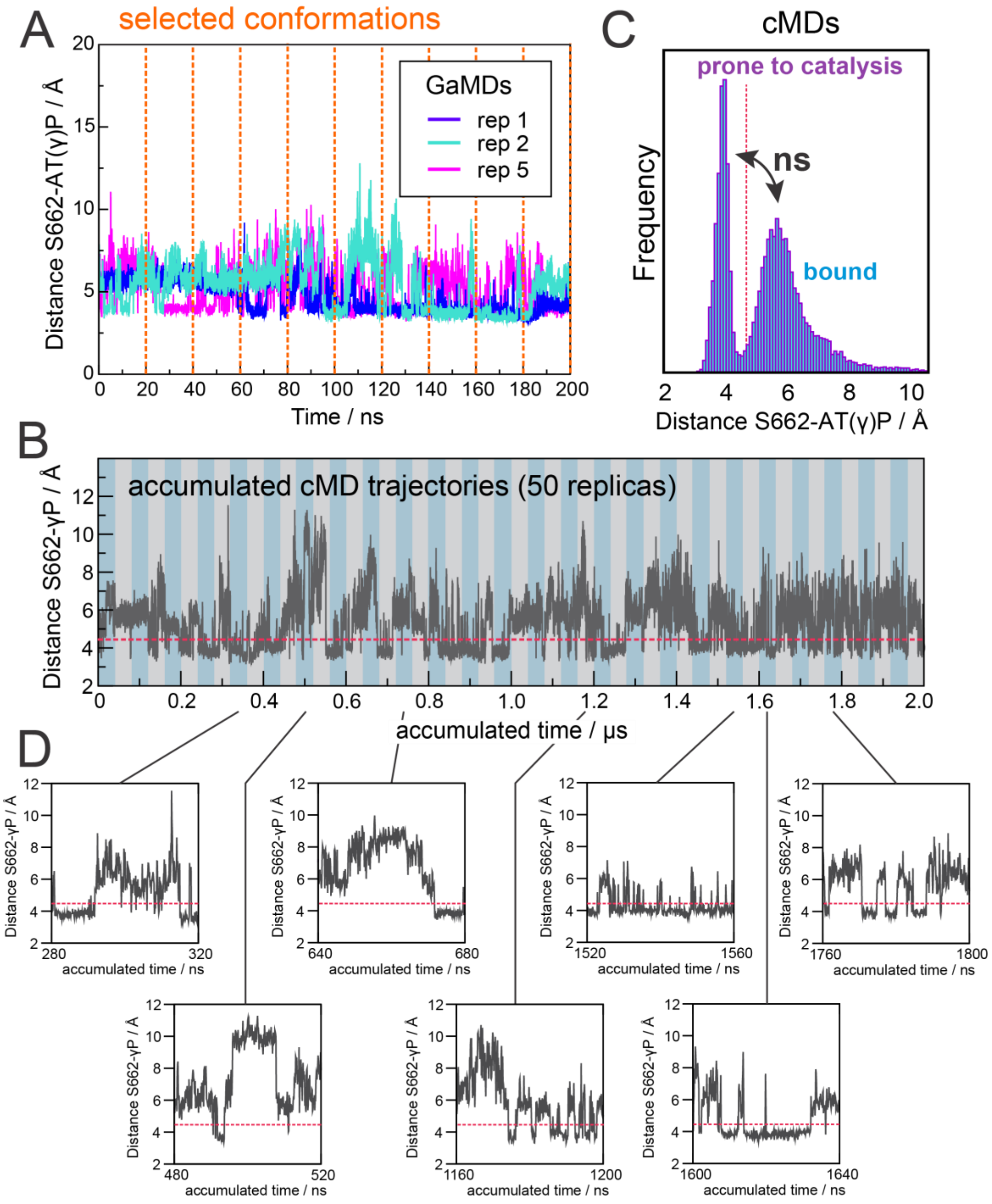

Figure S12. Final (cMD refined) model of FASP-bound CK1. A) Snapshots extracted from GaMD trajectories to be used as starting points to run cMD simulations. B) Distance between S662 (γ oxygen) and the γ phosphorous atoms from ATP calculated during the cMD simulations (each shaded stripe represents an individual replica). C) Distribution of the distances between S662 and AT(γ)P in the accumulated cMD simulations. D) Examples of binding mode transitions within individual cMD replicas.

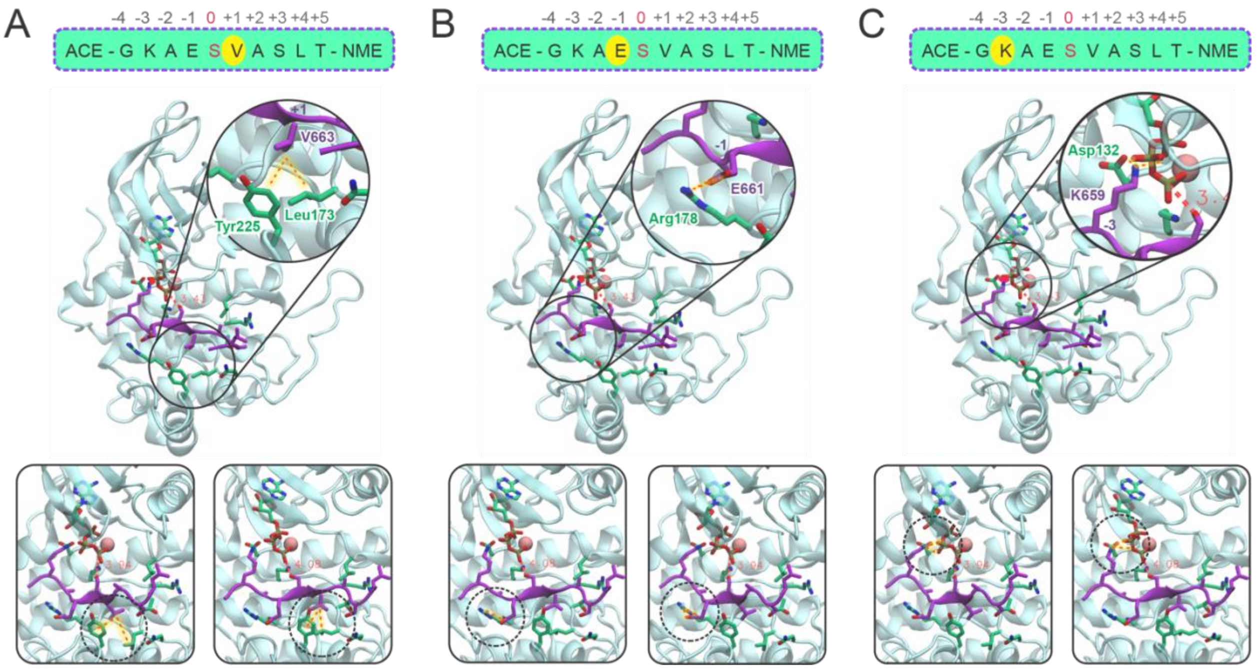

Figure S13. MD snapshot illustrating the interactions displayed between CK1 and FASP. A - C) Electrostatic interactions between V663 at position +1 (A), E661 at position -1 (B), and K659 at position -3 (C) of FASP with CK1.

Table S6. Definitions of how the distances reported in Figure 4 of the main manuscript were calculated.

Distance	Selection 1	Selection 2
V663 to Tyr^{225}	Res 663 (FASP) γ-carbons	Res 225 (CK1) γ-carbon, δ-carbons and ε-carbons
V663 to Leu^{173}	Res 663 (FASP) γ-carbons	Res 173 (CK1) δ-carbons
E661 to Arg^{178}	Res 661 (FASP) ε-oxygens	Res 178 (CK1) η-nitrogens
E661 to Lys^{224}	Res 661 (FASP) ε-oxygens	Res 224 (CK1) ζ-nitrogen
K659 to AT(β)P	Res 659 (FASP) ζ-nitrogen	ATP and γ-phosphate
K659 to Asp^{132}	Res 659 (FASP) ζ-nitrogen	Res 132 (CK1) δ-oxygens

D. Biochemical validation of CK1-FASP model with mutational experiments

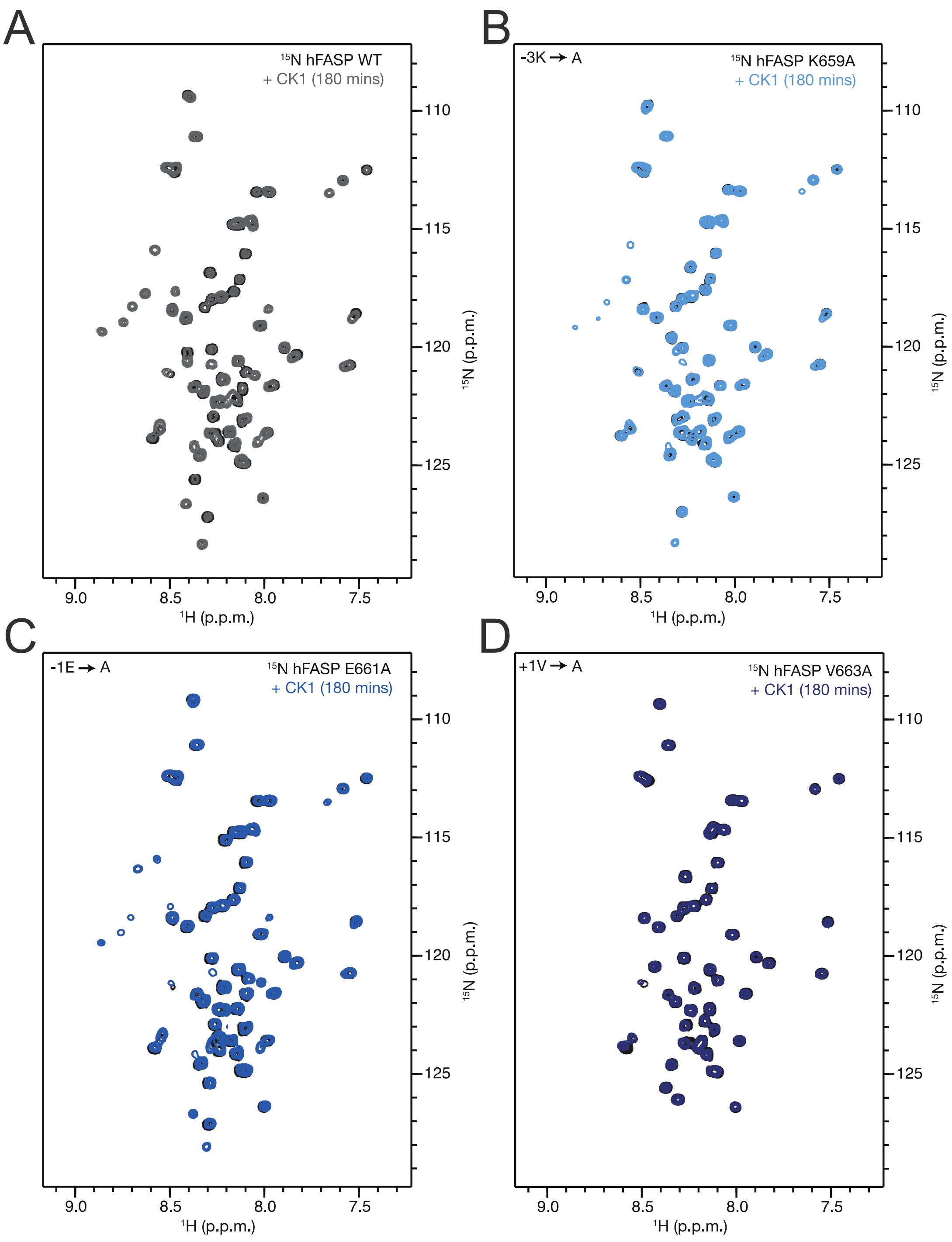

Figure S14. Biochemical validation of residues necessary for FASP priming. A - D) ^{15}N-^{1}H HSQC spectra comparing CK1 activity on human PER2 FASP WT (A) and alanine mutant FASP peptides K659A (B), E661A (C), and V663A (D) at a 3 hour timepoint.

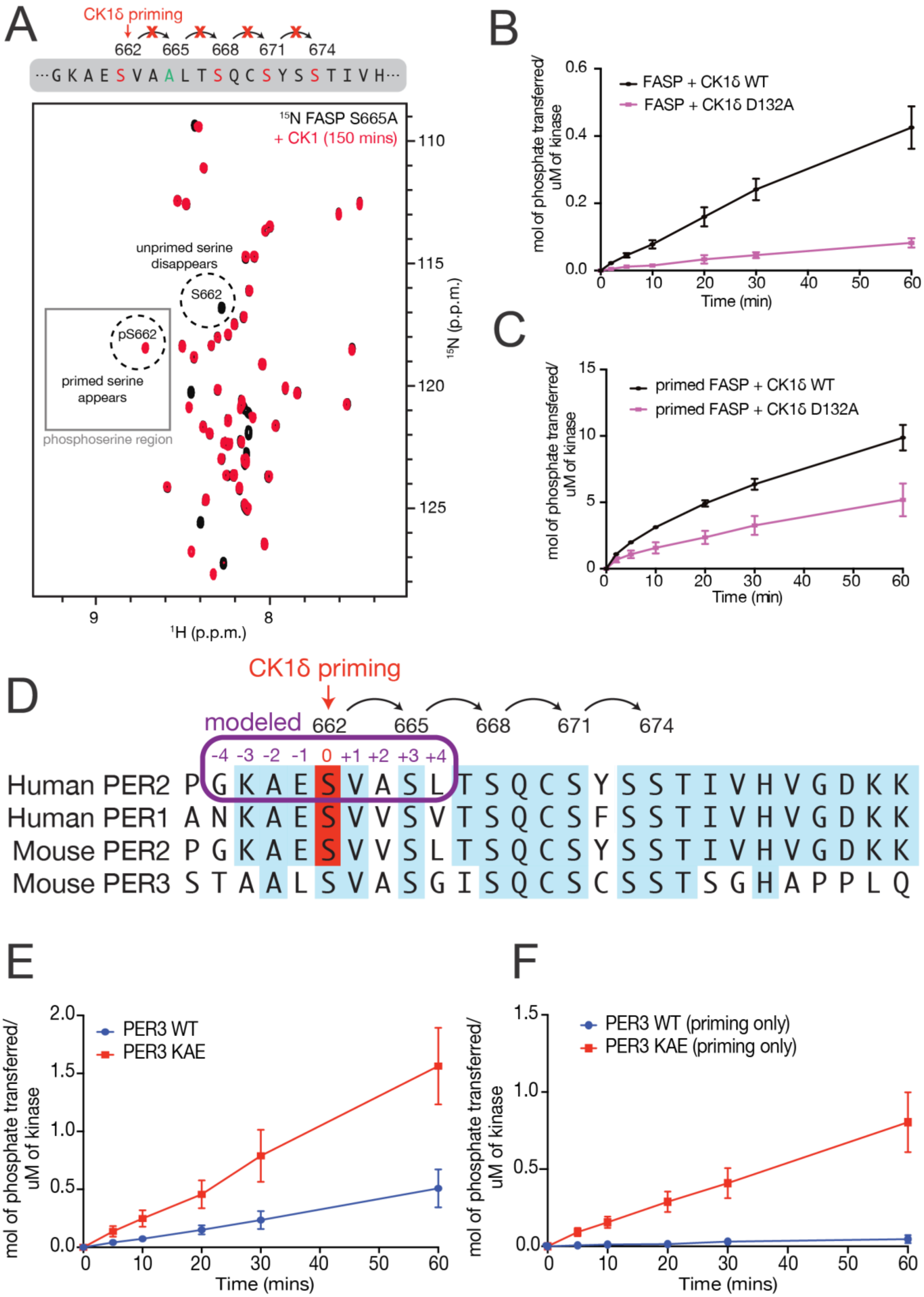

A
CK1δ priming
662 665 668 671 674
···G K A E S V A A L T S Q C S Y S S T I V H···
15N FASP S665A
+ CK1 (150 mins)
unprimed serine disappears
S662
pS662
primed serine appears
phosphoserine region
110
115
120
125
15N (p.p.m.)
9
8
1H (p.p.m.)
B
mol of phosphate transferred/ uM of kinase
FASP + CK1δ WT
FASP + CK1δ D132A
Time (min)
C
mol of phosphate transferred/ uM of kinase
primed FASP + CK1δ WT
primed FASP + CK1δ D132A
Time (min)
D
CK1δ priming
modeled
662 665 668 671 674
-4 -3 -2 -1 0 +1 +2 +3 +4
Human PER2 P G K A E S V A S L T S Q C S Y S S T I V H V G D K K
Human PER1 A N K A E S V V S V T S Q C S F S S T I V H V G D K K
Mouse PER2 P G K A E S V V S L T S Q C S Y S S T I V H V G D K K
Mouse PER3 S T A A L S V A S G I S Q C S C S S T S G H A P P L Q
E
mol of phosphate transferred/ uM of kinase
PER3 WT
PER3 KAE
Time (mins)
F
mol of phosphate transferred/ uM of kinase
PER3 WT (priming only)
PER3 KAE (priming only)
Time (mins)

Figure S15. Biochemical validation of FASP priming model. A) Schematic depicting disruption of sequential kinase activity in the "priming only" human PER2 FASP peptide (S665A), along with ^{15}N-^{1}H HSQC spectra comparing activity between the WT and the "priming only" peptides at the 150 min timepoint of a CK1 kinase reaction showing no detectable downstream phosphorylation activity within the S665A peptide. B-C) ^{32}P-ATP timecourse kinase reactions with synthetic mouse PER2 FASP peptides comparing activity on unprimed (B) and primed (C) substrates, between CK1 WT (black) and D132A (purple). D) Multiple sequence alignment of the FASP region from mammalian PER homologs with residue numbers corresponding to the human PER2 sequence. The -4 to +4 positions are labeled with respect to the priming serine (S662, red) and the purple box highlights the model peptide sequence from MD simulations. Blue shading indicates conservation of 75% or higher. E-F) ^{32}P-ATP timecourse kinase reactions with synthetic mouse FASP peptides corresponding to the WT PER3 sequence (blue) and a PER3 sequence with the -3 and -1 residues mutated to the corresponding PER2 residues at those positions (-3K and -1E, respectively) (red). F) ^{32}P-ATP timecourse kinase reaction as in panel E with serine residues downstream of the priming site mutated to alanine.

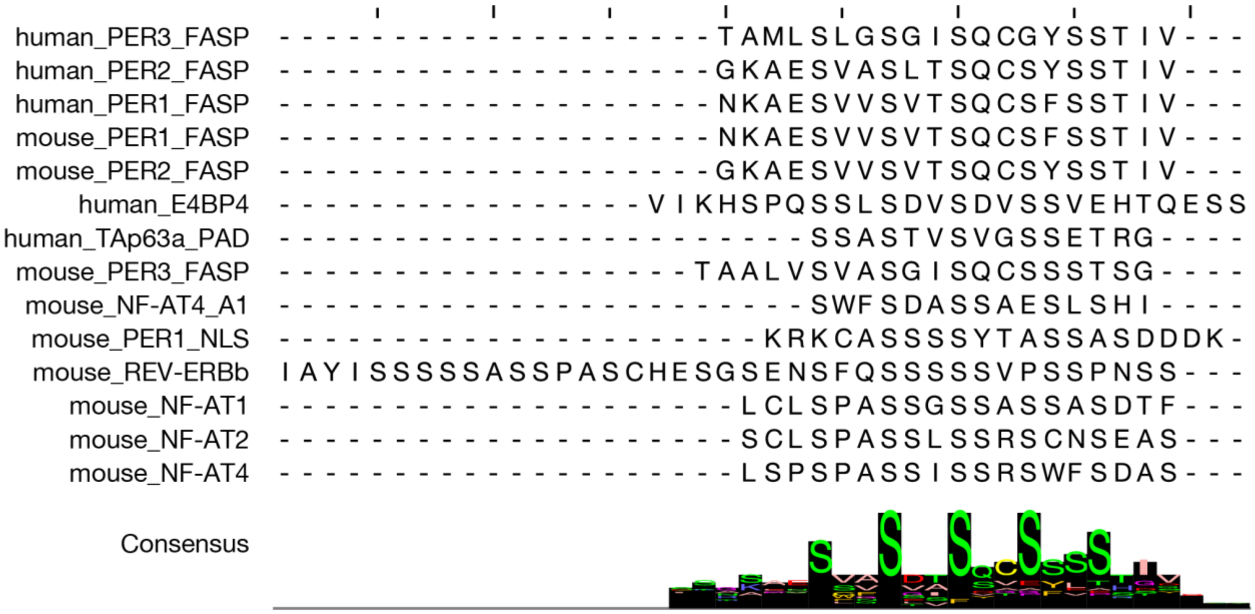

Figure S16. Alignment of CK1-targeted poly-SXXS motifs. Sequences for biochemically verified CK1 targets containing poly-SXXS motifs were identified from literature, aligned with Clustal Omega and displayed with Jalview. Mouse/human PER1 FASP [25], mouse/human PER2 FASP [26], mouse/human PER3 FASP (this work), human E4BP4 [27], human TAp63a PAD (NB: CK1 activity requires priming by CHK2) [28], mouse NF-AT4 A1 [29] , mouse PER1 NLS [30], mouse REV-ERBb [31], mouse NF-AT1, 2 and 4 [32].

E. Reshaping of the substrate binding cleft and identification of binding pockets on the CK1 surface

To map binding sites on the surface of CK1, we employed FTMap [33], a fast computational approach that uses small organic probes to identify consensus sites (or pockets) that are likely to bind drug-like molecules. We submitted a series of conformations representative of the most populated MSM states to the FTMap server (https://ftmap.bu.edu/) and analyzed the results with FTProd [34]. For the WT system, we submitted 6 conformations from state I (loop up) and 21 conformations from state III (loop down). For the *tau* mutant, we submitted 26 conformations from state I' (loop up) and 21 conformations from state IV' (loop down with loop EF unfolded). Tables S7 and S8 describe the consensus sites (CS) detected for WT and *tau* conformations, respectively. When a site is detected (colorful cells), it contains a score based on

the number of organic probes that docked in that site. When a cell is left blank, it means that FTMap did not detect that pocket in the corresponding CK1 structure.

Table S7. Consensus sites (CS) detected for WT CK1.

State	#	CS0	CS1	CS2	CS3	CS4	CS5	CS6	CS7	CS8	CS9	CS10
I (up)	1	38	-	3	-	19	-	7	-	4	3	4
I (up)	2	32	5	2	-	21	-	15	-	-	7	-
I (up)	3	51	-	2	10	4	-	11	-	2	4	-
I (up)	4	44	8	-	7	12	2	-	-	4	6	-
I (up)	5	58	3	2	-	-	-	-	-	7	12	-
I (up)	6	45	-	5	-	6	-	7	-	4	16	-
III (down)	1	60	-	-	-	-	3	-	-	3	15	-
III (down)	2	36	-	23	-	4	9	3	-	-	-	2
III (down)	3	33	41	3	7	-	-	-	-	-	-	-
III (down)	4	42	19	5	-	-	5	2	-	-	6	-
III (down)	5	18	27	26	-	4	-	8	-	-	-	-
III (down)	6	14	21	20	4	21	-	4	-	-	2	-
III (down)	7	18	49	-	2	3	-	7	-	-	2	-
III (down)	8	39	10	-	-	3	13	6	-	-	11	3
III (down)	9	20	-	17	-	17	-	11	-	12	-	-
III (down)	10	47	25	9	-	-	-	-	-	-	-	-
III (down)	11	21	38	-	8	-	-	-	-	-	14	2
III (down)	12	22	38	18	-	-	-	5	-	-	-	-
III (down)	13	22	43	12	-	-	-	-	-	-	4	-
III (down)	14	7	33	24	5	-	-	2	-	6	3	-
III (down)	15	15	-	24	33	-	-	-	-	-	3	-
III (down)	16	18	26	14	-	6	-	-	-	14	3	-
III (down)	17	8	11	33	14	3	-	2	3	2	2	-
III (down)	18	34	18	17	-	3	-	7	-	-	2	-
III (down)	19	34	18	19	-	2	3	-	-	3	-	-
III (down)	20	38	3	30	-	5	-	6	-	-	-	-
III (down)	21	27	42	4	-	3	-	2	-	-	-	-

Table S8. Consensus sites (CS) detected for *tau* CK1.

State	#	CS0	CS1	CS2	CS3	CS4	CS5	CS6	CS7	CS8	CS9	CS10	C11
III' (up)	1	33	23	-	-	-	7	-	3	-	4	-	6
III' (up)	2	28	30	-	11	8	-	-	-	-	-	-	-
III' (up)	3	20	31	-	2	16	-	-	-	-	12	-	-
III' (up)	4	34	25	-	2	10	9	-	-	-	-	-	2
III' (up)	5	-	23	-	21	-	24	-	3	4	5	2	-
III' (up)	6	4	40	-	3	24	7	-	-	-	5	-	-
III' (up)	7	13	27	-	31	-	10	-	-	-	-	-	-
III' (up)	8	28	20	-	4	27	-	-	-	-	-	-	-
III' (up)	9	15	38	15	3	6	-	-	-	-	-	3	-
III' (up)	10	16	22	-	27	-	12	-	-	-	-	-	2
III' (up)	11	36	28	-	4	8	-	-	-	-	-	-	-
III' (up)	12	17	24	5	2	16	-	-	10	-	2	-	-
III' (up)	13	17	24	5	2	16	-	-	10	-	2	-	-
III' (up)	14	6	32	-	-	15	25	-	-	-	4	-	-
III' (up)	15	37	26	-	13	-	3	-	-	-	-	-	-
III' (up)	16	32	36	-	5	8	-	-	-	2	4	-	-
III' (up)	17	19	16	7	-	17	5	-	-	-	16	-	-
III' (up)	18	29	15	4	4	15	3	-	-	6	-	5	-
III' (up)	19	33	26	2	-	-	-	-	8	-	9	2	-
III' (up)	20	9	21	-	10	31	2	-	-	-	5	-	-
III' (up)	21	37	39	-	4	-	-	-	-	-	-	-	-
III' (up)	22	10	16	-	26	11	-	-	-	8	14	-	-
III' (up)	23	45	17	8	4	4	-	-	-	-	-	-	-
III' (up)	24	32	23	-	17	8	-	-	-	-	-	-	-
III' (up)	25	-	24	16	19	12	5	-	-	-	-	-	-
III' (up)	26	45	21	-	-	5	9	2	-	-	-	-	-
IV' (down)	1	45	17	8	4	4	-	-	-	-	-	-	-
IV' (down)	2	17	17	-	11	-	20	7	-	-	4	-	-
IV' (down)	3	21	41	2	15	-	-	-	-	-	-	-	-
IV' (down)	4	28	12	-	28	7	4	-	-	-	-	-	-
IV' (down)	5	21	37	4	7	3	8	-	-	-	2	-	-
IV' (down)	6	18	18	2	11	-	29	-	-	-	2	-	2
IV' (down)	7	39	16	7	-	-	4	-	-	4	-	6	-
IV' (down)	8	43	19	-	12	-	7	-	-	2	-	-	-
IV' (down)	9	33	22	5	15	-	4	-	-	-	2	-	-
IV' (down)	10	15	21	7	-	4	27	-	-	-	5	-	3
IV' (down)	11	18	26	-	22	-	3	-	-	-	15	-	-
IV' (down)	12	29	23	7	4	-	-	-	-	-	13	-	5
IV' (down)	13	40	15	5	10	7	7	-	-	-	-	-	-
IV' (down)	14	41	22	5	7	-	2	-	-	-	-	-	-
IV' (down)	15	35	24	-	6	6	9	-	-	-	-	-	-
IV' (down)	16	23	19	4	5	-	17	-	-	9	3	-	-
IV' (down)	17	27	24	3	21	-	-	-	-	3	-	-	-
IV' (down)	18	27	22	-	11	8	-	8	-	-	-	8	-
IV' (down)	19	24	5	18	-	11	8	3	4	-	2	-	-
IV' (down)	20	43	12	2	2	-	6	-	-	6	8	-	-
IV' (down)	21	43	13	-	10	6	-	-	5	-	3	2	-

Table S9 describes the composition and location of the pockets detected in WT CK1, also illustrated in Figure S16. Not surprisingly, the top-scored pockets recapitulate the substrate binding cleft + active site (CS0), the ATP binding site (CS1) and the Mg^{+2} binding site (CS2), showing that FTMap is able to identify these functional pockets even in apo structures. Interestingly, the ATP and the Mg^{2+} binding sites are more likely to be closed or disassembled when the activation loop is up in the WT CK1 (compare scores and frequency of occurrence in 'up' and 'down' states). The next pocket detected in WT CK1 (CS3) is formed at the bottom of the kinase, between helices aG and aH.

Table S9. Composition and location of consensus sites identified in WT CK1.

CS	Description	Residues	$\langle score \rangle^{Up}$	$\langle score \rangle_{Down}$	$Occurrence^{Up}$	$Occurrence_{Down}$
0	Substrate binding cleft + active site	16 17 18 19 20 21 22 23 38 48 52 88 90 93 98 125 126 127 128 129 130 131 132 133 134 147 148 149 150 151 152 153 154 171 172 173 174 175 176 177 178 179 180 181 184 185 190 194 195 198 205 206 213 214 216 217 221 222 224 225 228 229	44.7	27.3	100%	100%
1	ATP binding site	13 15 16 17 23 24 25 36 37 38 52 56 66 80 81 82 83 84 85 86 87 88 89 90 91 130 132 133 134 135 138 148 149 150 151	5.3	27.2	50%	80%
2	Mg^{2+} binding site	16 17 18 19 20 21 22 23 38 39 40 44 45 46 47 48 49 52 55 56 130 132 148 149 150 151 152 153	2.8	18.1	83%	80%
3	FASP binding pocket	180 182 185 197 201 213 232 233 234 235 236 237 240 249 253 256	8.5	10.4	33%	33%
4	Back of the N-lobe	34 60 31 62 63 64 65 66 67 69 83 84 85 137 138 141 144 146	12.4	6.2	83%	57%
5	C-terminal pocket	99 100 101 102 105 204 206 207 208 210 211 212 240 242 243 244 245 248	2	6.6	17%	24%
6	Hinge pocket	13 84 85 86 87 92 136 137 138 139 142 143 286 287 288 291	10	5	67%	62%
7	2nd anion binding site	125 165 171 189 190	---	3	---	5%
8	'Activation' pocket	48 51 52 55 58 122 123 124 127 150 151 152 153 154 155	4.2	6.7	83%	29%
9	Back of C-lobe	63 106 107 110 111 114 115 139 140 141 142 143	8	16	100%	76%
10	Back of C-lobe	63 65 114 115 118 270	4	2.3	17%	14%

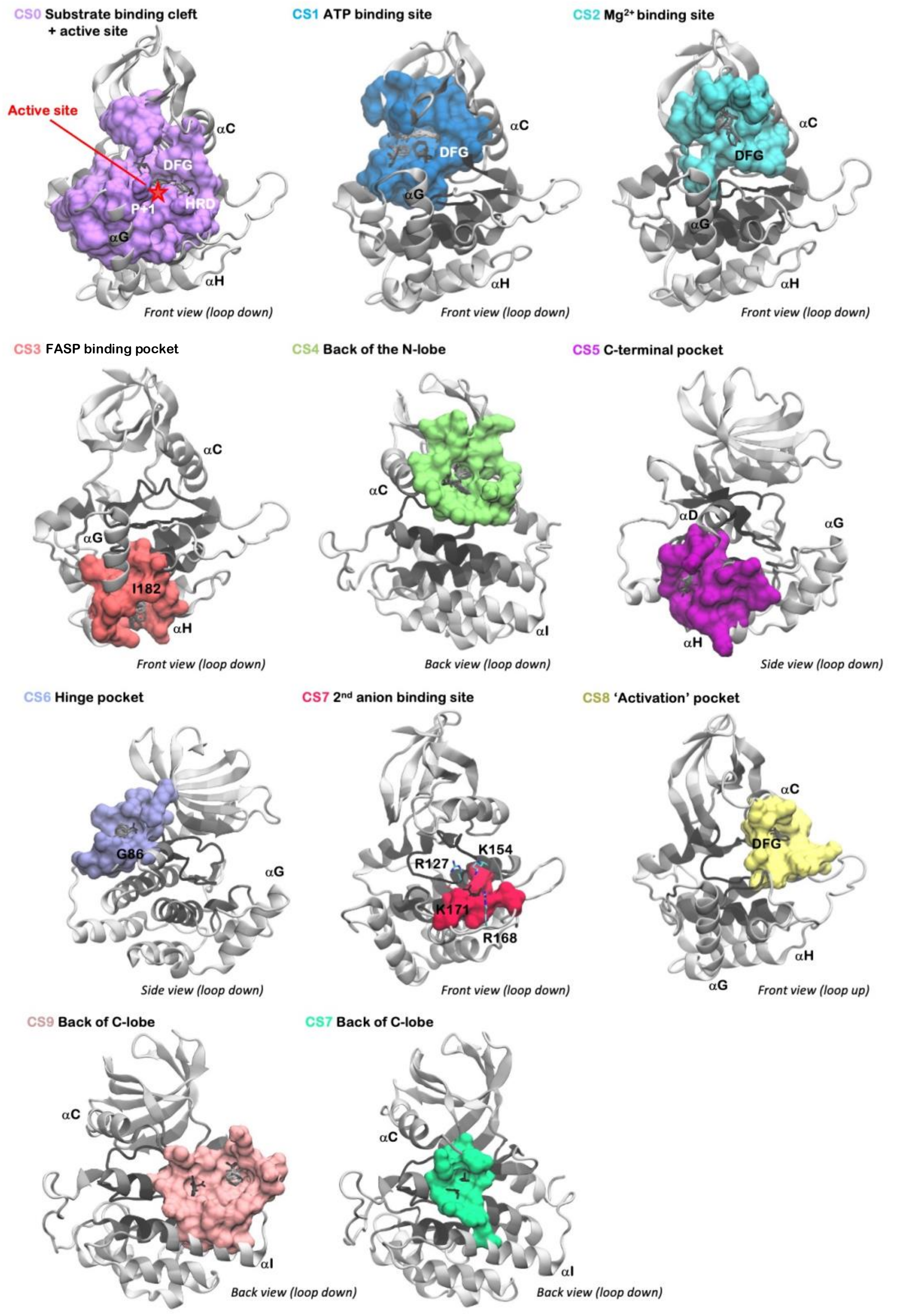

Figure S17. Consensus sites identified by FTMap on WT CK1.

Finally, Table S10 describes the composition and location of the pockets detected in *tau* CK1, also illustrated in Figure S17. As with WT CK1, the top scored pockets correspond to functional pockets including the ATP binding site (CS0) and the Mg^{2+} binding site (CS1). However, we observe a fragmentation of the active site and substrate binding cleft, which are split into three pockets according to FTMap: the active site is absorbed into CS1 (Mg^{2+} binding site), while the substrate binding cleft splits into CS3 (P+1 region) and CS5. The pocket located in the bottom between helices aG and aH is also detected and well scored in the *tau* mutant (CS2). Apart from these functional sites, the next pocket detected in *tau* (CS4) is located between the aC helix and the activation loop. It is noticeable that this pocket is more prominent when the activation loop is up (compare scores and frequency of occurrence in 'up' and 'down' states). An analogous pocket is also detected in the WT enzyme, but with poorer scores (see CS8 in Table S9), suggesting that this pocket is stabilized in the *tau* mutant.

Table S10. Composition and location of consensus sites identified in *tau* CK1.

CS	Description	Residues	<score>^{Up}	<score>$_{Down}$	Occurrence^{Up}	Occurrence$_{Down}$
0	ATP binding site	13 15 16 17 23 24 25 36 38 52 56 66 82 83 84 85 86 87 88 89 90 91 130 131 132 133 134 135 138 148 149	24.8	30.0	92%	100%
1	Mg^{2+} binding site + active site	15 16 17 18 19 20 21 22 23 36 37 38 39 40 41 44 45 46 47 48 49 52 56 80 81 82 126 127 128 129 130 132 133 148 149 150 151 152 153 171 173 174 175 176 177 179 180 221 222 225	25.7	20.2	100%	100%
2	FASP binding pocket	182 197 201 204 213 232 233 234 235 236 237 240 252 253 256	7.8	5.6	31%	67%
3	Substrate cleft (P+1 region)	90 98 130 131 132 174 175 176 177 178 179 180 205 210 211 213 214 216 217 221 222 224 225 228	10.2	11.2	81%	86%
4	'Activation' pocket	20 46 47 48 51 52 54 55 58 124 127 150 151 152 153 154 155 171 172 173	13.5	6.2	73%	43%
5	Active site + part of the substrate cleft	127 128 129 130 152 168 170 171 172 173 174 175 176 177 178 179 180 181 182 183 184 185 187 188 189 190 194 195 198 221 222 224 225 226 229 232	9.3	10.3	50%	71%
6	2nd anion binding site	125 127 154 155 156 157 164 165 167 168 173 189 190 191 192 193 259 260 262	2.0	6.0	4%	14%
7	Back of C-lobe	61 62 63 64 65 66 67 83 114 115 117 118 141 146	6.8	4.5	19%	10%
8	Back of N-lobe	8 9 28 33 34 35 67 68 69 81 82 83 84	5.0	4.8	15%	24%
9	Back of C-lobe	106 107 108 109 110 114 115 140 141 142 143 144 270 273 274 277 278 284 286 288	6.8	5.4	46%	52%
10	Hinge pocket	86 87 92 136 137 139 140 142 143 290 291	3.0	5.3	15%	14%

11	C-terminal pocket	93 98 99 100 102 105 206 207 244	3.3	3.3	12%	14%

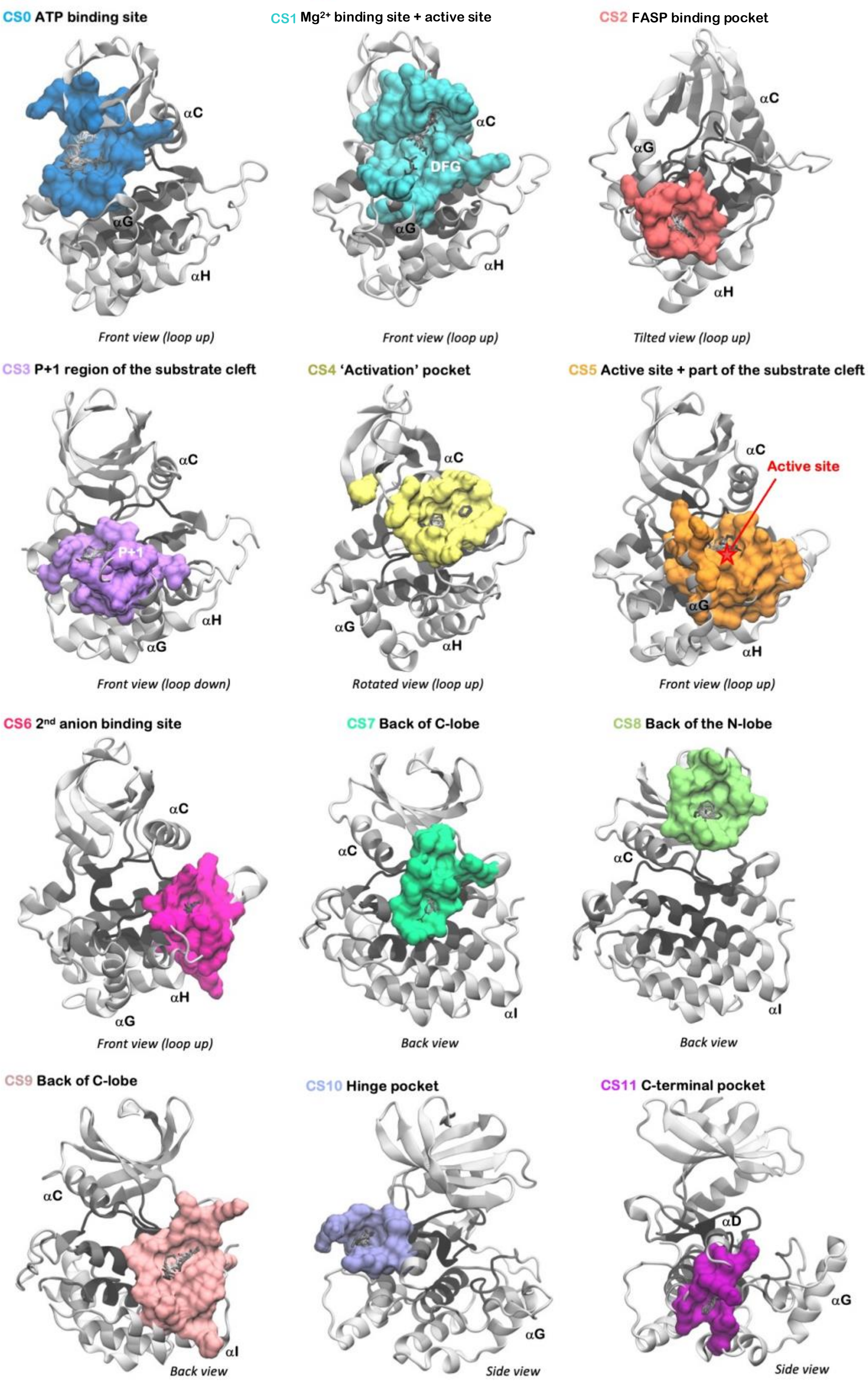

Figure S18. Consensus sites identified by FTMap on *tau* CK1.

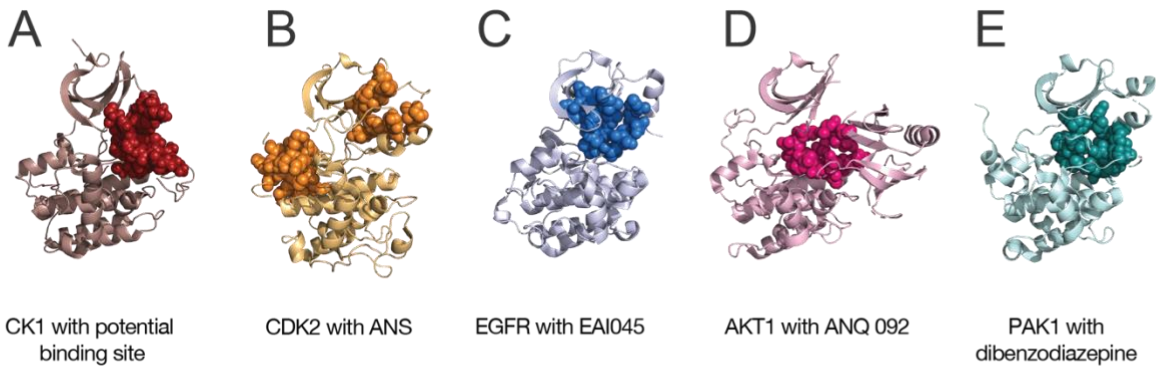

Figure S19. Comparison of binding pockets of kinase domains. A) CK1 'loop up' conformation (PDB 6PXN) [1] with potential binding site residues identified by FTMap represented as spheres (see Figure 7A). B-E) Structures of kinases bound to regulators were analyzed using PyMOL 3.1. Residues within 5Å of the regulator are represented as spheres and all non-protein atoms, including waters, ions, and ligands are removed. Chain A was selected for visualization from structures with multiple protein copies in the asymmetric unit. B) CDK2 in complex with two molecules of 8-anilino-1-naphthalene sulfonate (ANS) (PDB 3PXF)[35, 36]. C) EGFR kinase domain in complex with EAI045 (PDB 5D41)[36, 37]. D) Autoinhibited form of AKT1 in complex with ARQ 092 (PDB 5KCV)[36, 38]. E) PAK1 in complex with 2-chloro-5-ethyl-8-fluoro-11-(4-methylpiperazin-1-yl)(dibenzodiazepine)(PDB 4ZJI)[39].

F. Data availability and reproducibility

All the initial structures and scripts used to launch the simulations that were used to create the MSM models, as shown in Figure S1B, are provided at github.com/cpartch/CK1.

In addition to the initial structures, several representative structures with different degrees of membership to each of the metastable states described in the MSM model (states I, II and III (WT) and I', III' and IV (Tau) in Figure 2) are provided. These structures can be used to reproduce the structural characterization of the metastable states, as shown in Figure 2 and Figure S6. Unfortunately, we do not have the original volumetric maps shown in Figure 6, because they were generated "on the fly" for visualization purposes only. One should be able to recreate them based on the representative structures from states III (WT, straight) and I' (Tau, bent), using VMD plugins such as the "volmap" tool. These are available at github.com/cpartch/CK1.

In addition to the Jupyter notebooks, the files that support the MSM models shown in the paper are provided. These PyEmma files are available at github.com/cpartch/CK1.

For instance:

"**WT_5distances_lag30steps_k200_clustering_kmeans.pyemma**" → this is binary pickle file, containing the serialized PyEmma object describing the cluster centers shown in Figure 2A (top panel), which were used to build the coarsed-grained MSM model shown in Figure 2C (WT model).

"**WT_5distances_lag30steps_k200_HMSMlag3ns_3states.pyemma**"→ this is another pickle file, containing the Markov State Model fitted to the discretized (clustered) MD data, which is shown in Figure 2C (WT model).

Similar files are provided for the other models in the paper. They can be loaded on the Jupyter notebooks previously provided to regenerate all the plots shown in the paper and SI, as long as the user has some knowledge of Python and access to the PyEmma documentation: http://emma-project.org/latest/

Alternatively, here is an explanation on to how to load these files and extract the MSM information required by the reviewer (example, transition matrices).

To load these files on a Jupyter notebook:

import pyemma

cluster_wt = pyemma.load(' WT_5distances_lag30steps_k200_clustering_kmeans.pyemma')

msm_wt = pyemma.load(' WT_5distances_lag30steps_k200_HMSMlag3ns_3states.pyemma')

→ cluster_wt is a PyEMMA clustering data source, i.e. it stores: cluster centers (the k-means centroids in feature/TICA space), assignments of each frame to a discrete cluster (.dtrajs), properties for the clustering result (like .n_clusters, .clustercenters, .assignment for individual points). Key attributes:

cluster_wt.dtrajs → list of NumPy arrays, one per trajectory, each with integer cluster labels.

cluster_wt.clustercenters → NumPy array of shape (k, n_features).

cluster_wt.n_clusters → number of clusters (here 200).

→ msm_wt is a BayesanMSM PyEmma object, since we are using the Bayesian method to derive the final MSM models. It contains Transition matrix samples, stationary distributions (populations in equilibrium), implied timescales, and metastable assignments (if you run PCCA on it). Key attributes:

msm_wt.sampled_models → list of transition matrices sampled from the posterior matrix

msm_wt.stationary_distribution → mean stationary distribution (equilibrium populations)

msm_wt.sample_stationary_distributions() → posterior samples of equilibrium populations

msm_wt.metastable_assignments → PCCA-based metastable state memberships

Supporting References

1. Philpott, J.M., et al., *Casein kinase 1 dynamics underlie substrate selectivity and the PER2 circadian phosphoswitch.* Elife, 2020. **9**.
2. Jorgensen, W.L., et al., *Comparison of simple potential functions for simulating liquid water.* The Journal of Chemical Physics, 1983. **79**(2): p. 926-935.
3. Maier, J.A., et al., *ff14SB: Improving the Accuracy of Protein Side Chain and Backbone Parameters from ff99SB.* Journal of Chemical Theory and Computation, 2015. **11**(8): p. 3696-3713.
4. Wang, J., et al., *Development and testing of a general amber force field.* J Comput Chem, 2004. **25**(9): p. 1157-74.

5. Kashefolgheta, S. and A. Vila Verde, *Developing force fields when experimental data is sparse: AMBER/GAFF-compatible parameters for inorganic and alkyl oxoanions.* Phys Chem Chem Phys, 2017. **19**(31): p. 20593-20607.
6. Case, D.A., Betz, R.M., Cerutti, D.S., Cheatham III, T.E., Darden, T.A., Duke, R.E., Giese, T.J., Gohlke, H., Goetz, A.W., Homeyer, N., Izadi, S., Janowski, P., Kaus, J., Kovalenko, A., Lee, T.S., LeGrand, S., Li, P., Lin, C., Luchko, T., Luo, R., Madej, B., Mermelstein, D., Merz, K.M., Monard, G., Nguyen, H., Nguyen, H.T., Omelyan, I., Onufriev, A., Roe, D.R., Roitberg, A., Sagui, C., Simmerling, C.L., Botello-Smith, W.M., Swails, J., Walker, R.C., Wang, J., Wolf, R.M., Wu, X., Xiao, L., Kollman, P.A., *AMBER 2016*. 2016: University of California, San Francisco.
7. Darden, T., D. York, and L. Pedersen, *Particle mesh Ewald: An <i>N</i>·log(<i>N</i>) method for Ewald sums in large systems.* The Journal of Chemical Physics, 1993. **98**(12): p. 10089-10092.
8. Pérez-Hernández, G., et al., *Identification of slow molecular order parameters for Markov model construction.* Journal of Chemical Physics, 2013. **139**(1).
9. Scherer, M.K., et al., *PyEMMA 2: A Software Package for Estimation, Validation, and Analysis of Markov Models.* Journal of Chemical Theory and Computation, 2015. **11**(11): p. 5525-5542.
10. Wehmeyer, C., et al., *Introduction to Markov state modeling with the PyEMMA software [Article v1.0].* Living Journal of Computational Molecular Science, 2019. **1**(1): p. 5965.
11. Prinz, J.H., J.D. Chodera, and F. Noé, *Spectral Rate Theory for Two-State Kinetics.* Physical Review X, 2014. **4**(1).
12. Röblitz, S. and M. Weber, *Fuzzy spectral clustering by PCCA plus : application to Markov state models and data classification.* Advances in Data Analysis and Classification, 2013. **7**(2): p. 147-179.
13. Deuflhard, P. and M. Weber, *Robust Perron cluster analysis in conformation dynamics.* Linear Algebra and Its Applications, 2005. **398**: p. 161-184.
14. Kube, S. and M. Weber, *A coarse graining method for the identification of transition rates between molecular conformations.* Journal of Chemical Physics, 2007. **126**(2).
15. Noé, F., et al., *Projected and hidden Markov models for calculating kinetics and metastable states of complex molecules.* Journal of Chemical Physics, 2013. **139**(18).
16. Chodera, J.D., et al., *Bayesian hidden Markov model analysis of single-molecule force spectroscopy: Characterizing kinetics under measurement uncertainty.* arXiv preprint arXiv:1108.1430, 2011.
17. Ho, B.K. and R. Brasseur, *The Ramachandran plots of glycine and pre-proline.* Bmc Structural Biology, 2005. **5**.
18. Gebel, J., et al., *p63 uses a switch-like mechanism to set the threshold for induction of apoptosis.* Nat Chem Biol, 2020. **16**(10): p. 1078-1086.
19. Martínez, L., R. Andreani, and J.M. Martínez, *Convergent algorithms for protein structural alignment.* Bmc Bioinformatics, 2007. **8**.
20. Sondergaard, C.R., et al., *Improved Treatment of Ligands and Coupling Effects in Empirical Calculation and Rationalization of pKa Values.* Journal of Chemical Theory and Computation, 2011. **7**(7): p. 2284-2295.
21. Olsson, M.H.M., et al., *PROPKA3: Consistent Treatment of Internal and Surface Residues in Empirical Predictions.* Journal of Chemical Theory and Computation, 2011. **7**(2): p. 525-537.
22. Meagher, K.L., L.T. Redman, and H.A. Carlson, *Development of polyphosphate parameters for use with the AMBER force field.* Journal of Computational Chemistry, 2003. **24**(9): p. 1016-1025.
23. Miao, Y., V.A. Feher, and J.A. McCammon, *Gaussian Accelerated Molecular Dynamics: Unconstrained Enhanced Sampling and Free Energy Calculation.* J Chem Theory Comput, 2015. **11**(8): p. 3584-3595.

24. Case, D.A., Betz, R.M., Cerutti, D.S., Cheatham III, D.E., Darden, T.A., Duke, R.E., Giese, T.J., Gohlke, H., Goetz, A.W., Homeyer, N., et al., *AMBER 2017*. 2017: (University of California, San Francisco).
25. Philpott, J.M., et al., *PERIOD phosphorylation leads to feedback inhibition of CK1 activity to control circadian period.* Mol Cell, 2023. **83**(10): p. 1677-1692 e8.
26. Toh, K.L., et al., *An hPer2 phosphorylation site mutation in familial advanced sleep phase syndrome.* Science, 2001. **291**(5506): p. 1040-3.
27. Doi, M., et al., *Negative control of circadian clock regulator E4BP4 by casein kinase Iepsilon-mediated phosphorylation.* Curr Biol, 2004. **14**(11): p. 975-80.
28. Tuppi, M., et al., *Oocyte DNA damage quality control requires consecutive interplay of CHK2 and CK1 to activate p63.* Nat Struct Mol Biol, 2018. **25**(3): p. 261-269.
29. Marin, E.P., A.G. Krishna, and T.P. Sakmar, *Disruption of the alpha5 helix of transducin impairs rhodopsin-catalyzed nucleotide exchange.* Biochemistry, 2002. **41**(22): p. 6988-94.
30. Takano, Y., et al., *The RING finger protein, RNF8, interacts with retinoid X receptor alpha and enhances its transcription-stimulating activity.* J Biol Chem, 2004. **279**(18): p. 18926-34.
31. Ohba, Y. and H. Tei, *Phosphorylation of N-terminal regions of REV-ERBs regulates their intracellular localization.* Genes to Cells, 2018. **23**(4): p. 285-293.
32. Okamura, H., et al., *A conserved docking motif for CK1 binding controls the nuclear localization of NFAT1.* Mol Cell Biol, 2004. **24**(10): p. 4184-95.
33. Kozakov, D., et al., *The FTMap family of web servers for determining and characterizing ligand-binding hot spots of proteins.* Nat Protoc, 2015. **10**(5): p. 733-55.
34. Votapka, L. and R.E. Amaro, *Multistructural hot spot characterization with FTProd.* Bioinformatics, 2013. **29**(3): p. 393-394.
35. Betzi, S., et al., *Discovery of a potential allosteric ligand binding site in CDK2.* ACS Chem Biol, 2011. **6**(5): p. 492-501.
36. Mingione, V.R., et al., *Allosteric regulation and inhibition of protein kinases.* Biochem Soc Trans, 2023. **51**(1): p. 373-385.
37. Jia, Y., et al., *Overcoming EGFR(T790M) and EGFR(C797S) resistance with mutant-selective allosteric inhibitors.* Nature, 2016. **534**(7605): p. 129-32.
38. Lapierre, J.M., et al., *Discovery of 3-(3-(4-(1-Aminocyclobutyl)phenyl)-5-phenyl-3H-imidazo[4,5-b]pyridin-2-yl)pyridin-2-amine (ARQ 092): An Orally Bioavailable, Selective, and Potent Allosteric AKT Inhibitor.* J Med Chem, 2016. **59**(13): p. 6455-69.
39. Karpov, A.S., et al., *Optimization of a Dibenzodiazepine Hit to a Potent and Selective Allosteric PAK1 Inhibitor.* ACS Med Chem Lett, 2015. **6**(7): p. 776-81.
